# Supplementary material for: General practitioners’ everyday clinical decision-making on psychosocial problems of children and youth in the Netherlands
Source: PLoS One. 2022 Dec 28;17(12):e0278314. doi: 10.1371/journal.pone.0278314 (PMC9797081; doi:10.1371/journal.pone.0278314)
Supplement: S1 Box — (DOCX) [file pone.0278314.s001.docx]

| **Box 1. Vignettes - A ‘(suspected) psychiatry’, B ‘multidimensional problems’ and C ‘safety’.** |
| --- |
| Vignette A ‘(suspected) psychiatry’ (Dave, 6 years old) 1. *Mother visits the GP, together with Dave* School has advised mother to talk to her GP about Dave. School is wondering whether Dave has autism or ADHD because of concentration problems, difficulties in dealing with frustrations and bad moods in situations where Dave does not get his way.  2. *(some time later) – Dave’s mother and father visits the GP, without Dave* Dave’s behaviour at school has been problematic since the beginning of second grade. He often refuses to do his work and his behaviour annoys his classmates. Dave is easily distracted. He also looks for distractions during work activities. However, at home, he finishes his homework in a couple of minutes. Currently Dave is undergoing a clinical examination by a paediatric neurologist because he is thought to have epileptic attacks. Dave’s parents ask their GP what could be causing their son’s behaviour (‘ADHD?’) and how they should handle his upbringing. The GP discovers that a local youth and family team is already involved, without his knowledge. Dave’s parents want to ask the neurologist whether their son’s bedwetting is ‘normal’ or ‘abnormal’ at his age.  At home, during dinner, the parents often notice that they have to call Dave back to the table. He often refuses to tidy up. When Dave is away from home, he doesn’t show any problematic behaviour. Therefore, father thinks that his son’s ears are fine. Dave has an inquisitive nature and there are many subjects he would like to know more about. For example, he is looking forward to conversations with the GP. Mother has a family history of ADHD. Mother’s brothers have been diagnosed with motor control deficits. Dave’s parents want to know what’s wrong with Dave and how to cope with his behaviour.   3. *(some time later) Dave’s mother visits the GP, together with Dave* The situation at school is deteriorating. Dave’s mother is very concerned. She is talking to a special education generalist at school, who is affiliated to the local youth and family team. Diagnostic questionnaires have been conducted to explore the symptoms of his concentration problems, social difficulties and autistic behaviour.  Vignette B - ‘multidimensional problems’ (Sanne, 14 years old) 1. *Sanne’s mother visits the GP, alone* Sanne, a girl with below-average intelligence, attends training college. She frequently has temper tantrums at home when she doesn’t get her way. She is the oldest of five children. Her youngest sister is 1.5 years old. Her younger brother also has below-average intelligence. Sanne’s other brother and sister are tired of all her tantrums.  2. *(some time later) Sanne’s mother visits the GP, together with Sanne* Sanne’s mother has physical issues (‘osteopenia’) and is therefore not able to work. She is angry with school because they aren’t keeping the promises they made: Sanne doesn’t have to attend gym class because of her painful knees. Sanne often refuses to go to school because of bellyaches. She is also being bullied by her classmates. Sanne shows very structured behaviour at home. She always puts her things on the same places and gets angry if people move them. She has set times for her morning ritual. If things go differently, she immediately thinks she will be too late for school. Sanne has difficulties falling asleep at night because of restlessness. Sanne’s father works full-time and her mother has the responsibility for a large part of the childrens’ upbringing. Sanne’s mother and father often disagree with each other when it comes to raising their children. Sanne’s father is less patient with Sanne’s moods.  3. *(some time later) Sanne’s mother visits the GP, together with Sanne* Sanne’s family is cramped for space. Her parents have financial problems but no debts. Sanne is expected to run the household and to take care of her sisters’ upbringing, which she enjoys. Sanne’s mother is not able to do this herself because of her painful hands. Sanne’s mother complains that school makes a big deal out of Sanne’s help in the household. She says that school is threatening to inform the GP about Sanne’s situation.  Vignette C - ‘safety’ (Melany, 15 years old) 1. *Melany’s mother visits the GP, alone* Melany’s mother describes her daughter’s behaviour, which is causing tension at home. Melany won’t follow the rules and her performance at school is poor. Melany frequently drinks alcohol at a place called ‘the Shed’. She often comes home too late.  2. *(some time later) Melany’s mother visits the GP, without Melany* Melany’s parents got divorced 7 years ago. Melany lives with her father part of the week and with her mother the other part. Melany’s mother has a new partner. They are not living together. Melany and her father often have conflicts. When Melany misbehaves, her father gets very angry. Melany’s mother doesn’t want to talk about these conflicts, because father’s angry moods were one of the reasons for their divorce. School feels that Melany’s parents are giving their daughter too much freedom and too little support in her homework. Melany doesn’t want to be controlled by her parents while doing her homework. She says that she only studies if it’s really necessary.  3. *(some time later) Both parents visit the GP, with Melany* Melany is having difficulties finding rest. She is always contacting her classmates on her phone. She feels depressed regularly and worries a lot. She cannot come up with things she is good at or things that her parents are proud of. She isn’t motivated to study and doesn’t even know why she does any work. She often forgets to do her homework. However, when she does study, she gets good grades. She often gets angry at teachers who treat her unjustly. Some people say she doesn’t have any feelings. |
