# Supplementary material for: General practitioners’ everyday clinical decision-making on psychosocial problems of children and youth in the Netherlands
Source: PLoS One. 2022 Dec 28;17(12):e0278314. doi: 10.1371/journal.pone.0278314 (PMC9797081; doi:10.1371/journal.pone.0278314)
Supplement: S2 Box — (DOCX) [file pone.0278314.s002.docx]

| **Box 2. Interview guide.** |
| --- |
| Interviews *General questions*: 1a. Name?  b. Age? 2a. Number of years working as a GP?  b. Number of years working in current general practice? 3. Frequency of encountering psychosocial problems in children and youths during office hours? 4. What do you know about…  ⚫ …the Youth Care Act (2015)?   ⚫ …local youth and family teams?   ⚫ …regional specialized child mental health care?  *Questions regarding vignettes* 1. What did you think when you read this vignette?  ⚫ First impression?  ⚫ What do you think is the problem here?  ◌ What else do you need to investigate in this problem (e.g. anamnesis? diagnostic   tests?)  ◌ Did you make a diagnosis? If so, what was your diagnosis? Why?  ⚫ Do you recognise this vignette from your own clinical practice?  ◌ If so, what did you think back then? How did you explore the problem?  ⚫ What is your plan for management or referral?   ◌ What do you need in order to come up with a plan for management or referral (e.g.  anamnesis? diagnostic tests?)  ◌ What plan would you usually follow when you encounter such cases?  2. How did you come up with this plan for management or referral?   ⚫ To what extent would the following information influence your plan for management or referral?  ◌ child’s/parents’/sisters’ or brothers’/school’s/... preferences?  ◌ child’s/parents’/sisters’ or brothers’/school’s/... norms and values?  ◌ feasibility of the plan for the child/parents/… (e.g. treatment compliance)  ◌ conflicting opinions child vs. parents, child/parents vs. GP?  ◌ biomedical factors: medical history, (psycho)medication  ◌ social factors: illness behaviour, request for help, advice given by   others, dependency on social environment, loss of social contacts, loss of privacy  ◌ psychological factors: child’s own ideas on the problem, knowledge,   behaviour, (mental) handicaps  ◌ social economic status  3. If you chose to ‘solve’ the problem yourself:  ⚫ why do you want to try to solve the problem yourself?  ⚫ how are you planning to solve the problem?  ⚫ when would you decide to refer the child to another youth care provider?  4. If you chose to refer the child:  ⚫ why did you refer the child?  ⚫ to which youth care provider did you refer it to  ◌ youth care physician?  ◌ paediatrician?  ◌ youth and family team?  ◌ specialized child mental health care (child- and youth psychiatrist)  ◌ mental health nurse practitioner (MHNP)  ◌ other youth care provider?  ⚫ how are you planning to refer this child (e.g. using internet, by telephone)  ⚫ why did you refer to this youth care provider in particular  ◌ rational reasons?  ◌ (social)emotional reasons?  ◌ previous collaboration experiences with this youth care provider?  ⚫ current collaboration experiences with this youth care provider:  ◌ what processes run smoothly (e.g. speed of communication, quality   of written feedback?)  ◌ which points could be improved (e.g. communication)? *Additional questions* 1. What is your definition of ‘collaboration’ (consulting/referring/physical meetings/…?)  2. What is your definition of youth mental health care, youth and family teams, …? 3. Do you consider psychosocial problems in children and youths to be mainly a problem of the individual or a   problem of the child’s social system? |
