# Supplementary material for: General practitioners’ everyday clinical decision-making on psychosocial problems of children and youth in the Netherlands
Source: PLoS One. 2022 Dec 28;17(12):e0278314. doi: 10.1371/journal.pone.0278314 (PMC9797081; doi:10.1371/journal.pone.0278314)
Supplement: S2 File — (DOCX) [file pone.0278314.s004.docx]

**INTERVIEWS GEPSEUDONIMISEERDE DATA**
**Interview Hsdj9**Algemeen:
***Hoe lang bent u al huisarts?***20 jaar. Ik heb 20 jaar deze praktijk, ik ben officiëel 21 jaar huisarts. In <naam plaats> is dit mijn vijfde locatie, inmiddels in die 20 jaar maar ik heb 20 jaar mijn eigen praktijk. Aanvankelijk als solist, nu ook weer als solist maar nu in een HOED.
***Alvast een beetje toespitstend op het onderwerp waar we het zometeen over gaan hebben, hoe vaak komt het voor dat u op uw spreekuur gezinnen met psychosociale problematiek tegenkomt?***
Dat is een ruim begrip: ''gezinnen met psychosociale problematiek'' - betreft het vader, moeder, kinderen, combinatie van dat alles? Het kan van alles zijn, maar ik kan toch wel zeggen dat er dagelijks zich een probleem voordoet. Een psychosociaal probleem, laten we het zo zeggen. Dat kan zijn: echtscheidingsproblematiek, dat kan relatieproblematiek zijn, opvoedingsproblemen, gedragsproblemen dus noem maar op. Dagelijks. De ene dag heb je misschien één probleem en soms heb je dagen dat het er wat meer zijn, je hebt zo van die periodes in het jaar dat het wat allemaal wat moeilijker lijkt te gaan (in de winter bijvoorbeeld). In de zomer lijken een heleboel psychosociale problemen in één keer als sneeuw voor de zon verschenen. Wie weet is dat ook wel zo, maar dagelijks doet er zich dus wel een probleem voor. We hebben ook dagelijks werk voor de praktijkverpleegkundige (GGZ) die overvolle agenda's heeft dus dat geeft al aan dat er regelmatig mensen komen met psychosociale problematiek.
***Jullie hebben ook regelmatig contact over die patiënten (huisarts en POH-GGZ)? Hoe gaat dat dan in de praktijk?***We hebben een praktijkverpleegkundige, die is psychologe. Die ziet patiënten die ik verwezen heb en zij noteert alles in het patiëntendossier, dus ik zie wat er besproken wordt en wat er met de patiënt overlegd wordt en als het nodig is hebben wij samen overleg daarover. Zij kan zelf doorverwijzen en als dat niet kan, dan doe ik het. Of dat nou naar GGZ is of naar andere 2e- of 3elijns instellingen, dan doe ik dat. Doorverwijzen naar praktijken in omgeving doet zij vaak of zij neemt ook contact op met Centrum voor Jeugd en Gezin.
***In het kader van onze onderzoeksvraag is dat ook een belangrijk aspect, het JGT, ik zou daar graag zo nog wat meer over willen doorvragen. Omdat u al aangeeft dat u veel samenwerkt met de POH-GGZ in de praktijk is het misschien ook goed om in de casuïstiek een punt aan te geven waarop u zou zeggen: ''nu zou ik doorverwijzen naar de POH-GGZ''.***Dat is goed.

Casus 1 (Dave):
1. Moeder komt alleen, school denkt aan autisme of ADHD vanwege concentratieproblematiek, moeilijk met frustratie kunnen omgaan, kwaad worden...
***Het is vrij weinig informatie die nu wordt gegeven, maar wat is de eerste indruk die u nu krijgt?***In ieder geval heeft de school er al naar gekeken, er zal dus of een leerkracht of misschien wel een schooladviesdienst zich mee bemoeid hebben en wat duidelijk is dat gedacht wordt aan een diagnose die verder uitgezocht moet worden. Dat zal wel verwijzen worden.
***En u zou dan verwijzen naar...?***Hangt een beetje van de problematiek af, als het echt aanleiding geeft tot gedragsproblemen of schoolprestaties die achter lopen. Ik zie hier al staan ''moeilijk met frustratie kunnen omgaan, kwaad worden''. Dat geeft toch wel aan dat er gedragsproblematiek is, dan zal ik over het algemeen verwijzen om dit verder uit te laten zoeken. Dat is vaak ook wel de wens van de ouders, die worden gewoon gestuurd door school. Er zijn ook vaak gesprekken al geweest op school dat het niet goed gaat met, in dit geval, Dave en daar moet je wat mee. Als huisarts kom je daar, vind ik dan, niet heel veel verder mee, dus dan moet je verder onderzoek doen en mensen begeleiden.
***Zijn er op dit moment nog bepaalde dingen die u zou willen vragen aan moeder?***Hoe de ontwikkeling tot op heden is geweest. Hij is zes jaar dus ik mag er vanuit gaan, ik denk altijd in klassen, maar dan zou 'ie in groep 3 moeten zitten - zo'n beetje, hoe zeg maar zijn peuterperiode is gegaan omdat ik daar zelf geen inzicht in heb. En wat de aard van de problematiek is, of hij vriendjes heeft, hoe hij thuis is en hoe hij op school is. Ik moet een beetje een indruk krijgen van: wat is de ernst van de problematiek?

2. Ja, dat is nogal wat. In ieder geval problematisch gedrag, dat vermoedde ik al. Ook omdat er al bij één wordt gesproken van het feit dat hij kwaad wordt, dat hij moeilijk met frustratie kan omgaan en dat er aan autisme gedacht wordt. Als daar aan gedacht wordt, dan heb ik zelf al zoiets van: dat ligt één graadje hoger dan de reguliere psychologe, eigenlijk, dus dan zit ik al snel aan - als ik daar ook aanwijzingen voor kan vinden - aan het <naam specialistische GGZ-instelling> te denken, om daar verder te kijken maar goed dat is voor ouders misschien een stap te ver. Thuis gaat het ook niet helemaal zoals zou moeten, dus we hebben een paar problemen: we hebben gedragsproblematiek, zoals op school als thuis, ADHD wat aan moeders kant voorkomt. Toch ook wel mogelijk wat ontwikkelingsproblemen, broers van moeder hebben motorische problemen - vraag is of dat relevant is. En, ja de vraag van school: ''zou er sprake kunnen zijn van autisme of ADHD'' is ook iets wat verder uitgezocht moet worden. Dit maakt het wel wat complexer, ik zie dus ook dat hij terug moet naar de kinderneuroloog dus daar is op zich ook wel een insteek. Hij is dus kennelijk al van de kinderarts naar de kinderneuroloog en dan verbaast het me wel dat hij daar in de ontwikkeling - dat het daar kennelijk niet opgepakt is, of zo (dat daar verder niets mee gebeurd is); dat ouders met deze vragen bij mij komen.
***U had het zojuist ook al over wat de vraag vanuit school is en dat dat verder uitgezocht moet worden. Wat is uw plan van aanpak daarin, is dat met name: houdt u de ouders leidend in het verkrijgen van informatie of probeert u via andere kanalen nog informatie te krijgen over de situatie omtrent Dave?***Als je geluk hebt heeft de school een brief opgemaakt waaruit blijkt wat de problematiek is. Maar ja, op deze leeftijd zijn de ouders wat mij betreft leidend, inderdaad. Zij moeten met de informatie komen en zij moeten ook met een hulpvraag komen, vind ik. Het is niet alleen de onderwijzer/onderwijzeres die het hele project aanzwengelt. Je komt 't nog wel 's tegen dat ouders min of meer gestuurd worden en dat ze zelf zoiets hebben van: ik zie die problemen niet of dat heeft Jantje, Pietje, Klaasje ook of vader ook en dat is allemaal goed gekomen. Zolang de schoolresultaten en het gedrag daar geen aanleiding toe geeft, sta ik niet te springen om alle kinderen een etiketje op te plakken. Er moeten wel echt problemen zijn, het probleem moet bij kind of bij ouders of bij beiden zijn.
***Interessante punten, in ieder geval. Dan stel ik voor om een stapje verder te gaan. Daar komt opeens het JGT om de hoek kijken.***

3. Onhoudbaar geworden, moeder maakt zich ernstige zorgen - kan me voorstellen. Zijn gesprekken op school met orthopedagoog, er is een vragenlijst afgenomen op school, autistisch gedrag en sociaal probleemgedrag. Nou, daar moet je wat mee.
***Doet dit uw beleid veranderen?***Nou ja, ik gaf al aan dat het nogal multiproblematiek is als ik dat bij item 2 lees, dus dat een psycholoog daar niet 1 2 3 uit komt. Dus, ik denk dat ik inderdaad naar de kinderpsychiatrie danwel centrum voor autisme zou verwijzen om te testen.
***We zien het woordje al staan: JGT. Hoe vaak komt het voor dat u daarmee samenwerkt?***Nooit, ik hoor nooit wat. Als ik mensen daar naar toe verwijs moet ik van patiënten of van de ouders horen wat daar gebeurt. Voor mij is het een black box, dat was in het verleden al zo en dat is nu niet veranderd - vind ik.
***En een black box, hoe moet ik dat voor me zien?***Nou, ze nemen contact op met het JGT, en worden al dan niet gezien. Ze volgen natuurlijk wel vervolggesprekken, maar ik krijg nooit terugkoppeling eigenlijk dus ik weet niet wat daar besproken en gedaan wordt. Als ik er niet gericht naar vraag bij de ouders, weet ik niet wat daar gebeurt. Voor mij is het een beetje wazig. Als ik bijvoorbeeld verwijs, we hebben hier in <naam plaats> <vrijgevestigde GGZ-praktijk> (kinderpsychologie). Daar krijg je gewoon een keurig verslag van, die adviseren dan soms om door te verwijzen naar de tweede lijn. Maar goed, dat gebeurt allemaal gemotiveerd dus ik weet wat daar gebeurt. Van JGT hoor ik nooit wat, ik zou niet eens weten wie daar werken - bij wijze van spreken. Het kan aan mij liggen, hoor. Ik verdiep me misschien daar niet genoeg in, dat is de waan van de dag. Maar als je me vraagt naar een mening, van ''wat zegt het je, het JGT?''. Nou, op dit moment weinig.
***En als ik het goed begrijp, zit hem dat met name in het stukje communicatie - die terugkoppeling.***Ja, ik ken de mensen daar niet en ik hoor niks terug van ze.
***En dan bijvoorbeeld de samenwerking met de kinderpsycholoog, waar u het zojuist over had. Naast de communicatie die dan als positief ervaart, zijn er nog andere aspecten die u prettig vindt aan die samenwerking?***Korte lijnen en er is telefonisch overleg, wel 's. Ook daar ken ik de personen niet de kinderen zien, maar ik weet wel dat er korte lijnen zijn. Er is makkelijk overleg, er is snelle toegang, er is adequate schriftelijke communicatie en ze signaleren, ze behandelen en ik hoor ook van ouders dat het goed gaat, daar. Ik hoor zelden problemen. Dat schept vertrouwen van: ''als ik daar naar toe stuur'', wat je vroeger ook bij kinderartsen had met ADHD bijvoorbeeld, nou als ze daar zijn dan wordt het allemaal van je overgenomen en dan loopt het. Dat is bij andere instanties wel anders.
***Wij focussen ons binnen dit onderzoek ook op de samenwerking met specialistische GGZ, bijvoorbeeld <naam specialistische GGZ-instelling> in <naam plaats>. Hoe gaat dat dan, bijvoorbeeld qua communicatie, korte lijnen e.d.?***Het gaat wat digitaler, allemaal, dus je verwijst een kind. Ook daar heb ik wel de indruk, afgezien van de wachttijden, dat ouders en kinderen daar wel goed geholpen worden, verder. Maar dat is een indruk, dat vraag ik dan: ''hoe gaat het daar?'' en dan ''ja, we zijn daar en daar geweest en we zijn daar geweest'' en op zich loopt dat over het algemeen wel.
***Wederom interesante punten die u aansnijdt. Ik stel voor dat we naar de volgende casus gaan.***

Casus 2 (Sanne):
1. 2. en 3.
De praktijkschool, een soort VMBO of zo?
***Ja..***..ik denk het. Afgezien van de gedragsproblemen zijn hier ook wat meer sociale problemen, heb ik de indruk. Sanne zou wel een kind zijn waar van ik zeg: neem contact op met het Centrum voor Jeugd en Gezin. In eerste instantie kan daar 's gekeken worden van: ''wat is de problematiek wat daar rondom het kind speelt en is daar misschien wat in te structureren?'' en ik denk, in eerste instantie als ik dit zo lees, niet dat dit een kind is dat nou direct bij een psycholoog thuishoort maar het kan wel een uitkomst zijn dat het kind alsnog daar naar toe gaat wat betreft de aanpak van het kind. Ouders zitten niet altijd op één lijn in de opvoeding, vader heeft minder geduld: dus ook echt een interactie tussen ouders en kind dat niet helemaal optimaal loopt, misschien wat ze wat meer behoefte hebben aan structuur in het gezin. Het lijkt me dat dit een kind dat ik naar Centrum voor Jeugd en Gezin zou sturen, in eerste instantie.
***Als ik het goed begrijp, u beschouwt deze casus echt als een systeemprobleem?***Ja.
***Is het een herkenbare casus, die u in de dagelijkse praktijk zou kunnen tegenkomen?***Het is in zoverre herkenbaar dat ik wel 's vermoed dat zoiets speelt, maar dat het geen hulpvraag van de ouders is. Dus je signaleert het wel: wat een chaos hier, of op huisbezoek bij mensen waarvan je denkt: ''oh oh oh, dat dit allemaal goed gaat hier.'' dat gaat het misschien ook niet. Dus in die zin herken ik het wel, maar ouders komen daar niet mee, of het kind komt er niet mee of er zijn geen typische problemen. Maar het is wel bekend van huisbezoeken die je aflegt dat je soms in situaties terecht komt waarvan je denkt: ''nou, dat loopt hier niet helemaal zoals het in een standaard gezin gaat.''
***Dus u bent degene die het signaleert?***
Ja, ik signaleer het dan, maar het komt niet altijd ter sprake. Maar ik herken dit soort situaties wel en ja, opvoedingsproblemen en gedragsproblematiek op school: dat zijn natuurlijk wel dingen die je gewoon in de maatschappij hoort, maar daar hoeft niet altijd - dat gaf ik net ook al aan - een etiketje op geplakt te worden van een psychiatrische stoornis of een andere stoornis die behandeld moet worden door een psycholoog of iemand die zich daar mee bezig houdt. Ik kan me voorstellen dat sommige gezinnen gewoon zo functioneren en als het een probleem is, en in dit geval is het dat, dan schakel ik wel hulp in.
***En misschien is dit een open deur, maar vanaf wanneer wordt het voor u een probleem?***
Als je merkt dat kinderen daaronder lijden, dat er toch in de relatie met ouders, broertjes, zusjes, vriendjes, vriendinnetjes of op school problemen ontstaan; dan wordt het over het algemeen ook wel elders gesignaleerd, of dat je merkt dat tussen ouders onderling of in de relatie ouders-kind een probleem ontstaat, waar het kind danwel de ouders soms de dupe van zijn. Ik zie dat dan tegenwoordig vaak met excessief gebruik van computer, games of drugsgebruik, alcoholgebruik - dat zijn ook grote problemen die regelmatig aan de orde komen, hier. Ouders komen dan met: ''we hebben problemen met het gedrag van..'' - typisch pubergedrag, misschien. Maar soms kan dat ook tot ernstige problemen leiden in een gezin, waar andere kinderen ook weer de dupe van zijn. Dus dan is het voor het kind zelf niet een probleem, want die doet het zichzelf aan, maar ouders of broertjes/zusjes signaleren dat en hebben er wel last van; dat is een andere ingang dan dit.
***Komt het dan bijvoorbeeld ook voor dat u zegt van: ''ik zou graag met broertjes en zusjes apart willen spreken?''***Nee, dat komt eigenlijk niet voor. Die worden er door ouders bijna nooit in betrokken, maar ze vertellen het wel maar ik hoor eigenlijk nooit dat een broertje komt praten over zijn zusje - bij wijze van spreken.
***Dan nog een laatste vraag voordat we doorgaan naar de derde casus, alweer. We hadden het zojuist over dat - dat waren misschien eerder mijn woorden - maar dat het een systeemprobleem was. Is er ook iets wat u dan voor ouders zou willen organiseren (in de zin van hulp)?***Ik denk dat als ze gehoord worden en als ze begrepen worden in hun problematiek, dat je dan al een heel stuk verder bent. Het zijn ook ouders, dat als ze dat zouden willen - je moet je afvragen of dat het geval is, maar als je hulp biedt dat ze daarmee ook geholpen zijn. Als ze maar het idee hebben dat ze gehoord worden en dat ze aan de hand genomen worden, eigenlijk een soort consultatiebureau voor ouders. Vandaar in dit geval het Centrum voor Jeugd en Gezin, lijkt mij.
***Duidelijk verhaal, dan stel ik voor dat we naar de laatste casus - alweer - gaan***.

Casus (Melany):
1. 2. en 3.
Oh, nou, daar had ik het net al over. Dit is heel herkenbaar, kind dat toch tussen twee ouders leeft - eigenlijk - haar eigen gang gaat, ik denk aan het puberen is; haar geluk elders zoekt, misschien zelfs in alcohol. Ja, heel herkenbaar. Dit soort situaties zien we veel. Dit is iets wat bij wijze van spreken vanmiddag kan voorkomen.
***U noemde zojuist al de hulpvraag: wat willen ouders precies? Ik kan me voorstellen, in deze casus is dat wat lastiger omdat ouders gescheiden zijn en ieder hun eigen belang hebben. Wat doet u in zo'n situatie?***Toch met beide ouders praten, want je kunt niet met één ouder afspraken maken met betrekking tot hulp dus ik zou beide ouders uitnodigen. In eerste instantie, maar in tweede instantie met Melany. Misschien zelfs Melany alleen een keer, met één van de twee ouders. Misschien zelfs de ouders apart. Ik heb een eerder casus meegemaakt met een kind dat, zeg maar, door de week bij moeder was en in het weekend bij vader. Dat kind had astma en vader vond dat eigenlijk maar een onzindiagnose, dus die stopte elk keer met de medicatie in het weekend waardoor het kind op zondagavond weer benauwd terugkwam omdat vader ook rookte. Daarbij heb ik wel geleerd dat het verstandig is om in ieder geval beide ouders bij de behandeling te betrekken, ook bij spirometrie en dergelijke, om te voorkomen dat de ene dit doet en de ander dat of bepaalde vervolgstappen in twijfel trekt of anders handelt. Je kunt het niet altijd voorkomen maar je betrekt wel beide ouders, in dit geval. Dat zie ik nu trouwens ook staan, ouders komen samen - zonder Melany. Dus, ik denk dat dat aan de orde is en dan sondeer (?) ik, kijk ik waar de problematiek ligt: wie er problemen heeft en wat voor hulp er nodig is. Je kunt je helemaal richten op Melany, maar dat is ook niet helemaal de bedoeling. Ook ouders zijn debet zijn aan de problemen die op dit moment spelen. ***En stel: het komt voor dat vader iets compleet anders wil dan moeder en u merkt dat op het spreekuur. Wat doet u om dan toch die gemeenschappelijke hulpvraag te realiseren?***In dit geval hangt het sterk samen met wat Melany zelf wil, wat ze zelf vindt. Als ze een echte puber is, dan wil ze helemaal niets -natuurlijk. Maar, je moet toch een ingang zien te vinden omdat ze op school kennelijk ook niet gemotiveerd dus zo'n kind strandt gewoon op één of andere manier. En ik denk dat dat voorop staat, dat zij zich weer gelukkig gaat voelen, dat ze zich beter in haar vel voelt, dat ze op school weer beter presteert en dat het drankgebruik in ieder geval strikt beperkt wordt, waarbij ik me dan ook altijd afvraag: ''hoe komt het dat een kind van 15 al zo aan de drank zit?'' We hebben toch afgesproken voor je achttiende niet te drinken, dus wat doen de ouders daar aan om dat te voorkomen? Het blijft in dit geval een lastig probleem, waarbij de interactie tussen ouders en kind heel belangrijk is. Wordt het kind niet opgezet tussen de ouders, dat zie je ook nog wel regelmatig - dat het kind een soort speelbal is en dat de ene ouder het kind veel vrijer laat dan de ander en dat daardoor weer spanningen ontstaan. Dus dit is echt wat wat met alledrie, in dit geval, besproken moet worden. Daar heb je niet een panklare oplossing voor. Het is niet zo van (?) ''ik verwijs dit kind naar Jeugd en Gezin'' - dat het dan allemaal op z'n pootje terecht komt, dat is een lange adem. Maar moeilijk, ik denk dat ik ook hier Centrum voor Jeugd en Gezin zou inschakelen, om te kijken wat ze kunnen bereiken - in de hoop dat het daarbij blijft, maar het zou me niets verbazen dat ook met betrekking tot het gedrag er nog wel 's een psycholoog aan te pas zou kunnen komen voor het kind en misschien zelf voor één van de ouders: ''hoe om te gaan met?''
***Ik denk in ieder geval dat u een aantal belangrijke punten heeft gezegd, dus ik ben wel veel wijzer geworden in dit half uur. Ja, wat natuurlijk ook de insteek is van dit onderzoek is dat er een wetenschappelijk artikel van verschijnt en hopelijk ook internationaal gepubliceerd wordt: tenminste, het is de insteek om de Nederlandse situatie als illustratie te gebruiken vanuit internationaal perspectief. Ik wil ook graag in dat artikel komen tot een aantal aanbevelingspunten: bijvoorbeeld voor JGT's, maar ook voor huisartsen en voor specialistische zorg om toch die doorverwijzing te versoepelen, om die communicatie te versoepelen. Zijn er nog dingen waarvan u zegt: ''die mogen daar niet in missen, dat zijn heel belangrijke punten om mee te nemen?''***Nou, ik vind het altijd prettig als je degene naar wie je verwijst kent en dat er ook een soort terugkoppeling is. Het is te lastig, en dat heb je ook in de ouderenzorg, is het lastig om met iedereen gesprekken te hebben om met iedereen gesprekken te hebben en met iedereen overleg te hebben - dat is heel moeilijk. Maar ik merk wel dat als je bijvoorbeelt naar de case manager overlegt dat je weet: ''oh, dat is die case manager en die heeft dat stel of die betrokkene in behandeling en ik weet wie het is en ik kan er makkelijk mee overleggen'', dat je een soort vertrouwensband, net als met de praktijkverpleegkundige, dat je weet wat je aan iemand hebt, dat je ook weet wat het vervolgtraject is - dat je weet wat er gebeurt. Dus ik zou wat dat betreft wel inzicht willen hebben in het vervolg in zo'n Centrum voor Jeugd en Gezin, als ik iemand daar aanmeldt: ''wat gebeurt er dan feitelijk? met wie komen ze te spreken en wat zijn hun mogelijkheden om verder te verwijzen en daar ook een terugkoppeling van te krijgen?'' met name: het hoeven geen ellelange pagina's te zijn, maar even een terugkoppeling van: ''we hebben die en die gesproken, we hebben het daar over gehad, we gaan dat en dat doen - zodat je weet dat er actie ondernomen is.'' Ik gaf al aan, het is soms een black box, je stuurt iemand daar naar toe en als je dat niet zelf bijhoudt, kan het zijn dat ouders blijven zwemmen in een geheel waarvan je zegt: ''nja, worden ze eigenlijk wel adequaat geholpen?'' Dat is lastig.
***Waar zou dat aan liggen, denkt u, dat het zo'n black box is en dat de terugkoppeling..?***.. het is misschien een nieuwe situatie, voor iedereen. Niet alleen voor Jeugd en Gezin maar voor huisartsen ook en ik denk nog steeds de onbekendheid met het instituut dat de oorzaak is daarvan. Het speelt allemaal nog niet zo lang, natuurlijk. Ik heb ook niet altijd ervaring met dat instituut, meer met de praktijkverpleegkundige (de POH-GGZ), met de psychologe waarmee je samenwerkt, met de kinderpsychologe waarmee je samenwerkt, de tweede lijn waar je mee samenwerkt: dat is allemaal bekend terrein en dit is voor mij relatief onbekend - dus dat moet groeien.
***Zou het dan bijvoorbeeld helpen dat ze zich persoonlijk voorstellen of dat er een soort bijeenkomst wordt georganiseerd waarin mensen vanuit het JGT zich voorstellen aan huisartsen vanuit de regio, of - ik noem maar een initatief, dat kan natuurlijk van alles zijn.***Ja, dan moet je ook maar net kunnen - bij wijze van spreken, maar je moet natuurlijk altijd een eerste stap zetten, dus dat zou misschien nuttig zijn om toch wat meer bekendheid te creëren.
***Dat waren wat mij betreft alle vragen die ik wilde stellen. Zijn er nog dingen die u wilde toevoegen aan de hand van de dingen die we besproken hebben?***
Nee, boeiend. Ik ben benieuwd wat de uitkomst van het artikel is.

EINDE INTERVIEW
**Interview Hu5dm**Algemeen:
***Hoe lang ben je al huisarts?***Ik moet me in september registreren dus ik ben 10 jaar huisarts.
 ***En hoe lang al in deze praktijk specifiek?***In 2010 ben ik hier gekomen, in 2011 heb ik deze praktijk overgenomen, dus 6 jaar praktijkhouder.

***En alvast een beetje toespitsend op het onderwerp waar we het zometeen over gaan hebben: hoe vaak komt het nou voor dat hier kinderen en jongeren op het spreekuur komen die psychosociale problematiek hebben of waar een vermoeden van psychosociale problematiek is?***
En bedoel je nieuwe problematiek of bedoel je bestaande problematiek?
***Beide***.
Er zijn natuurlijk een aantal dingen die je zelf behandeld en die je zelf controleert en een aantal dingen die nieuw zijn. 2-3x per week? Waarvan 1-2x weken iets nieuws, zoiets? Denk ik.

***Nog redelijk frequent dus?***Ja.

***En werkt hier voor de rest een POH-GGZ?***Ja maar die doet geen kinderen.
***Dus daar hebben jullie ook geen overleg over?***Nee, we hebben op zich een kinderpsycholoog met wie we intens samenwerken. Dus daar heb je wel 's overleg, dus zeker over crisissituaties met jongeren- dan heb je daar wel overleg mee. Dus die weten we vrij makkelijk te vinden, als het nodig is. We hebben ook wel een goed CJG, dus daar redden we het ook wel mee (?)

***Daar komen we ook zo zeker over te spreken. Stel ik voor dat we alvast naar de casuïstiek gaan.***

Casus 1 (Dave):
1. En dan?

**Ja, dit is natuurlijk redelijk weinig informatie. Wat is een beetje het eerste beeld wat je krijgt als je dit zo op je spreekuur ziet?**
Dit wekt frustratie bij mij, omdat ik heel veel van dit soort dingen te zien en te horen krijg waarvan ik denk: waarom moeten we iedereen een stempel geven? Dus dat irriteert mij mateloos. Weet je, is het kind mee of is moeder zonder kind?
***Dit is samen met Dave, inderdaad***
Nou, dus Dave is erbij. En als ik Dave een beetje ken dan kan ik dat vaak een beetje inschatten, als ik Dave niet ken ben ik geneigd om Dave vragen te stellen omdat ik nieuwsgierig ben hoe hij reageert. Dat is het eerste. Weet je, ik denk een deel van mijn irritatie ontstaat uit het feit dat je hier niet onderuit komt. School heeft een bom neergelegd bij ouders, dan moet je van goede huize komen als je als huisarts zegt: hier doen we niks aan. Weet je, dus het is een beetje al irriterend omdat je weet dat je een verwijsbrief moet gaan schrijven waar je misschien helemaal niet achter staat, maar waar je niet onderuit gaat komen.
***Dus de eerste indruk is dat het irritatie oproept?***Bij mij wel. Weet je, bij mij hebben ze ook heel vaak het stempeltje ADHD willen plaatsen en ik heb nooit met een onderzoek meegedaan, dat weiger ik ook gewoon omdat ik denk dat enige onrust bij jezelf een goede eigenschap als huisarts is omdat je makkelijk out of the box kunt denken. Ja, dus dat is denk jeugdfrustratie van mijn kant. Dus ik denk dat het bij mij meer irritatie opwekt dan bij andere mensen omdat ik het onzin vind dat we al die kinderen maar een stempeltje moeten geven.
***Voordat we naar het tweede stukje gaan: wat zijn de dingen die je met name aan moeder zou vragen - bijvoorbeeld?***Wat je altijd wilt weten, want weet je: kinderen met concentratieproblemen, als die wel 2,5 uur achter een spelcomputer kunnen zitten dan moet jij je afvragen hoe ernstig de concentratieproblemen zijn. Weet je, als een kind concentratieproblemen heeft met dingen die hij niet leuk vindt, dat vind ik iets heel anders dan.. Weet je, je hebt kinderen met concentratieproblemen die wel een boek kunnen lezen. Weet je, ik kan geen krant lezen en ik kan geen boek lezen - dat houd ik ongeveer 1 minuut vol en dan ben ik alweer afgeleid. Dus ik geloof daar niet in. Autisme is iets heel anders dan ADHD, totaal ander ziektebeeld. Ik heb zelf in de verstandelijk gehandicaptenzorg gewerkt. Als je nou kinderen neemt onder de 4 en je neemt autisten en ADHD'ers - je weet wel, de echte - dan zie je overeenkomst. Maar daarna gaat zich dat uitsplitsen. Echte autisten haal je er ook wel uit, maar mensen die in de vergaarbak van Asperger en PDD-NOS zitten ja dat zijn de moeilijke maar dat zijn dezelfde als de mensen met ADD-stoornissen, die zitten ook helemaal in de vergaarbak van ellende want de klassieke ADHD'er die haal je er ook wel uit. Maar die ADD'ers is dezelfde vergaarbak als PDD-NOS en als je die twee over elkaar legt dan vind je echt wel raakvlakken. De vraag is een beetje: wat moeten we met al die mensen? Moeten we ze bestempelen, want medicatie hebben we niet. Die ADD'ers die krijgen dan wel 's Ritalin en dat soort dingen maar grosso modo werkt dat niet - het doet niks. Nou ja, dan moet je richting de stracturomedikinet (?) om te kijken of dat wat doet. Nou, ik heb daar een hard hoofd in dus nee - ik vind het moeilijk.

***Duidelijk verhaal tot dusver. Dan stel ik voor om in ieder geval meer informatie te geven door het tweede stukje...***2. *Leest* Ja zie je daar gaan we al... *leest*
***Wat is het probleem als je dit zo leest?***Er is helemaal geen probleem. Ouders hebben een probleem. Dave heeft in principe geen probleem. Het eerste wat mij irriteert is het feit dat het op school niet goed gaat. Ik kan me mateloos irriteren aan een Nederlands schoolsysteem dat een vrouwelijk systeem is en op één of andere manier zijn alle jongetjes het Sjakie. *richt zich op mij*: even voor jou, waar zet je een ADHD'er in de klas? Voor of achterin?
***Je zou in eerste instantie zeggen, voorin...***
Ja, maar het is achterin hè? Dan hoeft 'ie niet de hele tijd achterom te kijken om afleiding te zoeken. Dus, het feit dat je hier al hoort dat hij zich de hele tijd omdraait wil al zeggen dat Dave verkeerd in de klas zit. Dat ik denk: hoe moet ik als huisarts aan een onderwijzer moet uitleggen waar 'ie de kinderen in de klas moet zetten, dan gaat het al niet goed - zeg maar. Dan gaat er al een hoop niet goed op school. In de interactie tussen de leraren en Dave. Ja, je zou 's met die kinderneuroloog kunnen overleggen: hoe ernstig zitten we nou met die toevallen? Bedplassen, dan denk ik, als je genoeg problemen hebt... hoe oud is ze? Groep 3, leeftijd is 6. Ja, kan nog / mag nog. Vervelend. Vaak wel een uiting dat er toch nog iets van psychische nood is, zeg maar. Het gaat op school ook niet goed. Dat ik denk, als je het probleem nu niet oplost dan gaat het bedplassen ook niet beter. Het is een kind wat geïnteresseerd is en wat interesses heeft, wat verder waarschijnlijk best goed doet als je die stimuleert - nee, ik zie niet zoveel problemen.
***Ja, dus wat je noemt, het is met name het stukje met school - dat omgaan met ...***
***Is dat nog iets wat je met school zou willen overleggen, of doe je dat via-via?***
Nee, dat moeten ouders vooral doen. Als ik alle scholen hier in de regio moet gaan opvoeden dan word ik helemaal gek, dus daar ga ik niet aan beginnen. Ik vind niet dat het aan mij is om aan een ander uit te leggen hoe hij/zij zijn werk moet gaan doen. Dat is een discussie die ouders met school moeten *onverstaanbaar*. Ik heb zelf drie kinderen thuis, dat vind ik genoeg zeg ik altijd maar.
***Mooi gezegd. En wat betreft zorg voor ouders, want je noemde zojuist dat er ook iets met ouders aan de hand is...***... nou daar ga je het gesprek over aan en dan ga je kijken hoe zij er in staan en wat je daaraan kunt bijsturen. Er moet iets mis zijn met Dave en ze willen weten hoe ze daar mee om moeten gaan. Nou, dan moet je eerst kijken waar de hekele punten voor ouders zitten. Dan kijk je daar of je daar zelf iets mee kunt of dat je ze verwijst naar het CJG waar natuurlijk uitleg wordt gegeven over hoe om te gaan met gedragsproblemen bij kinderen. Of stuur ze naar <naam vrijgevestigde kinderpsycholoog> om daar hulp in te bieden. Er zijn meerdere wegen die mogelijk zijn.
***En als ik het goed begrijp, afhankelijk van de hulpvraag van ouders sla je een bepaald pad in?***Ja, en waar ik denk dat ouders het beste... hangt er een beetje van af hoeveel aandacht ze nodig hebben, hoe makkelijk ze het zelf oppakken. Hoe ernstig ik het inschat, dat bepaalt een beetje welke kan ik opga, zeg maar. <Naam vrijgevestigde kinderpsycholoog> is hier een psycholoog dus dat is een beetje 1 op 1, dus dat moet niet te complex zijn. Bij complexe problematiek kan je beter verwijzen naar het CJG want daar zitten veel meer loketten bij elkaar, zeker als het kind ook echt problematiek heeft - dan zit je bij het CJG veel beter omdat je daar veel meer faceten hebt - zeg maar - dan alleen één psycholoog. Dus dat is een beetje de keuze.
***Want hoe is dat contact voor de rest met het CJG? Hadden we het net natuurlijk even over.***
Op zich wel goed, kijk er is op zich natuurlijk heel veel geïnvesteerd sinds de Jeugdwet. We hebben een ''tentie(?)''-overeenkomst moeten tekenen als huisarts met de gemeente, daarna is er een plan gemaakt en omdat ik Hagro-voorzitter ben met gemeente in gesprek te gaan. Er gaat binnenkort een evaluatie komen van de werkafspraken zoals ze nu liggen. Het CJG wordt hier best wel goed aangestuurd dus dat loopt echt wel goed. Weet je, diep in mijn hart zou ik zeggen: er moet meer overleg zijn, maar ik heb daar zelf eigenlijk de tijd niet voor. Dan vraag ik eigenlijk iets wat ik niet kan bieden, zelf, dus dat is een beetje jammer. Maar als we het hebben over goede zorg, dan denk ik dat als we als huisartsen wat meer tijd zouden hebben en wat meer tijd hebben voor het overleg - maar ik ben ook misschien een controlfreak en een neuroot, dat ik misschien teveel wil weten. Maar dat zou mij iets meer rust geven. Ik heb wel bedongen dat wij de verslagen krijgen, van <integraal gezinsplan>. Dan ben je een beetje op de hoogte van welk hulpverleners betrokken zijn bij probleemgezinnen - zeg maar.
***Vanuit het JGT?***Ja, dus stel als er iets vastloopt dan kun je in dat plan terugvinden wie je kunt bellen. Dus dat vind ik al een meerwaarde wat we bewerkstelligd hebben. Maar over het algemeen gaat het wel goed, vind ik.
***Dat is goed om te horen, in ieder geval. Maar een verbeterpunt, als ik het zo beluister, zit met name in het stukje tijd.***Tijd aan onze kant, ja.
***Hoe zou dat opgelost kunnen worden? Hoe zou dat verbeterd kunnen worden?***Daar gaat die hele discussie van de laatste tijd over. Als wij naar een praktijk van 1800 patiënten gaan, dan zou dat een stuk makkelijker zijn - denk ik. Maar, ik denk dat er ook een boel huisartsen zijn die er geen behoefte aan hebben.
***En waar ligt dat dan aan, denk je?***
Dat ligt bij een stukje: waar ligt de eigen verantwoordelijkheid van de patiënt? Waar is het mijn verantwoordelijkheid en waar is het de verantwoordelijkheid van de patiënt? Als je bij alle huisartsen gaat toetsen: dan ligt die grens bij huisartsen verschillend. En ik denk dat ik 'm veel meer bij mezelf leg en wat minder bij de patiënt, dat maakt dat ik wat meer controle en het overzicht wil hebben. Ik denk dat heel veel collega's de grens bij de patiënt leggen en dan zeggen: ja, als de patiënt een probleem heeft dan komt 'ie wel langs. Als ze een probleem hebben, dan bellen ze wel.
***Ja en ik kan me zo voorstellen. Bij vrijwel alles in de huisartsengeneeskunde ben je als huisarts de poortwachter. En bij dit stukje alleen is de JGT ook een poortwachter, die kan ook doorverwijzen naar de specialistische GGZ.***Ja, maar soms verwijs je ook naar de JGT, juist zodat zij 'm dan weer doorverwijzen. Dus het kan allebei, maar als het JGT verwijst dan krijgen we ook een brief terug. Meestal staan wij in de correspondentie. Dus je blijft altijd wel op de hoogte van wat er gespeeld en wat er reilt en zeil, zeg maar. Alleen als we het in huis houden, dan denk ik dat je het niet altijd ziet. Als er echt van die <integraal gezinsplan>-verslagen komen, dan zie je het wel dus.
***Dus, die communicatie loopt wel goed als ik het zo beluister?***Ja.

***Gaan we alweer naar het derde punt..***

3. ***Zijn er op dit moment nou acties die door je hoofd schieten waarvan je zegt: dat heeft deze jongen nu nodig? Dat zou ik op dit moment inzetten of willen weten?***Nou ik denk dat 'ie psychologische zorg nodig heeft. Ik denk dat het belangrijkste is dat eerst een goede diagnose gesteld wordt. Het eerste wat ik me afvraag is waarom het JGT hem niet verwezen heeft, naar het <naam specialistische GGZ-instelling> - dat er diagnostiek gedaan wordt. Ik denk dat in dit geval je duidelijkheid moet hebben: als dat kind vastloopt dan moet je duidelijkheid hebben: een diagnose en een behandel plan. Nou, dan heb je kinder-GGZ nodig.

***Dat is het plan de campagne?***
Ja.

Casus 2 (Sanne):
1. 2. en 3. tezamen.
***Als je dit zo leest, waarin verschilt de problematiek van deze casus ten opzichte van die eerste casus?*** ***Wat is nou essentieel voor jou om een plan te bedenken?***Hier is het meer een welzijnsprobleem. Dit is een gezin wat net het hoofd boven het water kan houden, alle zeilen moeten bijgetrokken worden, iedereen moet bijspringen om dit ? te houden. Er zijn ook nog wat, moeder heeft nog wat klachten die haar ook weer beperken in haar draagkracht. Dus zit hier veel meer een welzijnsprobleem dan een zorgprobleem. En tuurlijk kun je zeggen dat Sanne wat OCD-achtige kenmerken heeft (kan ook bij autisme passen). Er kan ook nog wel een zorgprobleem rondom Sanne zijn, alhoewel ze toch 14 is en op school zit. Ik zie niet dat ze heel vaak is blijven zitten. Ik zie ook niet zo snel dat er op school problemen zijn behalve dan dat er in de interactie met medeleerlingen problemen zijn. Wat je natuurlijk wel vaker ziet, bij meisjes met laag sociaal-economische klasse en lagere intelligentie.
***Zie je dan ook meer pesten?***Ja, dat geloof ik wel, ja. Ja, dus ik denk dat -zeker vanwege financiële zorgen -. Laten we eerlijk zijn, ze zal niet de nieuwste kleren aanhebben, nou dan val je al snel op in de klas. Dus, dat zijn allemaal dingen. Misschien is het een meisje dat niet echt 'r best doet om er leuk uit te zien, misschien wat praktisch ingesteld is. Dat is natuurlijk ook een welzijnsprobleem.
***Want je noemt dit een welzijnsprobleem t.o.v. een zorgprobleem (wat die eerste casus was). Wat zou je plan nu zijn?***Hier moet veel meer maatschappelijk werk gaan komen, ondersteuning ipv. dat je hier een psychiater nodig hebt - in gradaties, zeg maar. Dus een welzijnsprobleem, dus veel meer begeleiden om alles op rit te houden dan dat je diagnostiek gaat bedrijven. Want qua diagnostiek zou je zeggen: nou, moet je Sanne diagnosticeren. Ja, omdat Sanne opzich niet vastloopt op school, dan moet je Sanne assertiviteit geven en omgaan met de ellende die ze moet meemaken dan dat ik Sanne zou willen diagnosticeren. Tenzij Sanne op school daadwerkelijk leerproblemen heeft, dan is het een ander verhaal. Maar als dat niet het geval is, dan heeft ze meer assertiviteitsbegeleiding nodig op psychisch vlak. En je moet een maatschappelijk werkster hebben om dit gezin helemaal goed in beeld te krijgen en daar waar nodig is bij te sturen. Daarom noem ik het een welzijnsprobleem en dit meer een zorgprobleem.
***Zou je het ook zo mogen zeggen dat dit meer systeemproblematiek is t.o.v. een individueel probleem?***Ja, alhoewel ik niet wil zeggen dat het bij casus 1 geen systeemproblematiek is want als je twee werkende ouders hebt, en een kind wat nog bedplast, dan zit daar ook een systeemprobleem aan. Dus ik wil niet zeggen dat het ene systeem... alleen... staat het systeemprobleem hier wat meer op de voorgrond dan bij de andere casus. Maar ik denk dat je bij casus 1 niet mag vergeten dat daar ook systeemproblematiek is. Hier staat het meer op de voorgrond, daarom noem ik het meer een welzijnsprobleem en dat je daarop gaat focussen - niet op de diagnostiekvoering en de zorg rondom het kind.
***Heel duidelijk, inderdaad***.

Casus 3 (Melany):
1.2. en 3. tezamen
Ja, leuk..
***Dit is inderdaad weer een heel ander ding. Is het iets wat je overigens vaak ziet, deze problematiek?***Ik zit in <naam plaats>, wat denk je zelf? Veel hoogopgeleiden, veel echtscheidingen... ja.
***En waarom zijn er dan hier meer echtscheidingen? Ben ik persoonlijk benieuwd naar.***Nou, het is misschien elders ook zo. Maar het is een heel bekend beeld waarbij financieel het over het algemeen wel goed geregeld is, die kinderen hebben misschien net iets meer te besteden dan ze nodig hebben. En dan zie je dit soort gedragingen wel is. En dan is het na zo'n scheiding dat de kinderen gekocht worden met cadeautjes en dingetjes en pa en ma hebben allebei een baan dus als ze thuis komen zijn ze moe. En dan die kinderen, als pa en ma moe zijn, dan krijgt het kind z'n zin.
***Een soort speelbal wordt het dan, dus?***Ja, maar ook dat het kind z'n zin krijgt. Dus het kind wordt niet opgevoed, omdat pa en ma te moe zijn voor die opvoeding. Opvoeding kost tijd en energie, dat is gewoon zo. Je moet consequent zijn in regels stellen en als je een hele dag gewerkt hebt dan ben je niet altijd in staat om dat goed te doen. En ja, dan kan dat zo escaleren.
***Eigenlijk dezelfde vraag als die ik bij casus 2 stelde: waarin verschilt deze casus nou ten opzichte van die andere twee?***
Ik denk sowieso: je hebt te maken met een 15-jarig kind, wat redelijk verantwoordelijkheid kan nemen voor zijn eigen gedrag, dus je kunt hier veel meer spreken over dat je met 3 volwassenen te maken hebt (alhoewel 15 jaar nog niet helemaal volwassen is), maar ik vind dat je een kind van 15 wel op z'n gedrag mag aanspreken. En ik denk niet dat Melany helemaal een goede keuze kan maken in hoe ze haar leven zou willen leiden, maar ik denk dat het goed is... hier zou ik dan <naam vrijgevestigde kinderpsycholoog> dan weer op afsturen. Die kan én het kind begeleiden én de ouders begeleiden in het vormen van een beeld: hoe gaan we samen met elkaar om? Aan de ene kant: Melany moet 'r verantwoordelijkheid nemen van school: die wil je daarin ondersteunen dat ze daarin een keuze gaat maken. Daarnaast wil je dat ouders leren samen een kind op te voeden en ik denk dat als je dat allemaal op rit hebt, dan wordt het gedrag van Melany vanzelf beter. Op dit gebied is het dan weer niet alleen maar een welzijnsprobleem. Dit is meer een relationeel systeemprobleem, zeg maar, wat je moet aanpakken en gaandeweg ook Melany moet helpen volwassen worden. Verantwoording te nemen voor haar daden.
***Waarbij je dus eigenlijk een gemeenschappelijk uitgangspunt met ouders en Melany creeërt?***Ja, je hebt dus een soort individueel traject voor Melany in hoe word ik volwassen en hoe neem ik verantwoordelijkheid voor mijn daden? Want dan pak je ook het drinkgedrag en jongensgedrag, die pak je daarin mee. En daarnaast is er een soort systeem-relationeel probleem, dat je zegt: hoe gaan we met elkaar om? Hoe maken we afspraken die we nakomen en hoe zorgen we ervoor dat Melany een leuke jonge meid wordt? Dat is denk ik het traject dat je dan wilt oppakken.
***Dus dat zou dan via <naam vrijgevestigde kinderpsycholoog> gaan?***Ja, dat is een kinderpsycholoog hier, die heel veel van dit soort trajecten van mij doet.
***En zou dan voor de rest het JGT nog in je hoofd opploppen? Het is natuurlijk ook systeemproblematiek.***Ja, voordat het CJG, voordat de nieuwe wet er was, hadden we een relatief goed werkend systeem hier - met <naam vrijgevestigde kinderpsycholoog>. Dan heb je ook nog <naam vrijgevestigde kinderpsycholoog> die heel veel doet met systeemtherapieën. Dus we hebben al heel veel op dat gebied, omdat deze problematiek eigenlijk al jaren bestaat - ook met één ouder-gezinnen enzo waar je ook dit soort problemen ziet. Dus ja, op dat gebied denk ik dat er best wel bij het CJG binnenkomt, wat wij niet zien maar dit soort dingen - nee, dit soort dingen, dat vind ik zo'n duidelijk verhaal. Want dit ken ik, dit snap ik en dit weet ik te verwijzen (?) want als je naar casus 2 kijkt, daar zitten veel meer facetten aan vast waar ik geen kaas van gegeten heb: ondersteuning bij financiën, ondersteuning bij hoe moet je je gezin nou runnen, dat zijn dingen: dat vind ik veel meer een casus voor het CJG dan casus 3.
***Dus eigenlijk afhankelijk van in hoeverre je zelf denkt die zorg te kunnen leveren, dus het pad denkt te weten...***... dat ik weet hoe het gaat lopen, daar kan ik dan voor kiezen. Terwijl in casus 3, dat is een casus die voor ons veel bekender is, dus waarvan we al een werkende oplossing hebben. Dat is al vrij makkelijk. In casus 2 daar zitten veel meer facetten aan vast die we NIET weten en daar om... En ik denk dat casus 3 ook prima via het CJG zou kunnen gaan. Weet je, als het omgaat via het CJG, dan komt heel veel van deze ook bij <naam vrijgevestigde kinderpsycholoog> uit - die werken ook samen-, maar dat zou binnen het CJG waarschijnlijk opgelost kunnen worden. We hebben hier nog ééntje samen, die ook heel veel met het CJG samenwerkt (<naam vrijgevestigde kinderpsycholoog> is dat). Dus als ?, dan gaat het ook goed, daar heb ik geen twijfel over. Als het bij mij komt, dan kies ik voor de bekende weg en dat zou <naam vrijgevestigde kinderpsycholoog> zijn.
***Een aantal belangrijke punten die je dan aansnijdt. De casus zijn afgelopen, maar zoals ik net al vertelde (uitleg streven naar aanbevelingspunten). Heb je daar nog aanbevelingen of tips voor, dingen die ik mee moet nemen in mijn verslag?***Nee, want ik vind niet dat het zo slecht gaat. Ik heb het gevoel dat het bij ons in <naam gemeente> dat het echt wel redelijk op de rit is. Ik heb wel gehoord dat het CJG hier ook weer een aanbeveling van de inspectie heeft gekregen. We hebben hier zelf een casus gehad van een mevrouw die voor een trein gesprongen is en die heeft twee kinderen achtergelaten en daar is door de inspectie een heel onderzoek naar geweest waar ik aan meegewerkt heb. We hebben daar een bijeenkomst over gehad, dat vond ik heel nuttig. Ik heb het idee dat we echt wel op dat gebied heel goed bezig zijn. Dus ik zie niet, en dat houdt bij de gemeente ook op (?) en dat is wat we ook nu gaan krijgen dat we die evaluatie hebben waarvan ik heb gezegd dat het wel geëvalueerd moet worden. Als je niet evalueert, op vaste tijden (1 keer per jaar of 1 keer per 2 jaar) op vaste tijden om de tafel gaat zitten, dan verwateren dingen en dan raak je het kwijt. Dus afspraken die je maakt, die moet je na blijven komen en die moet je toetsen dat je het nakomt. Dus ik denk dat de essentie is dat je zorg dat je faciliteert dat er een jaarlijkse of een tweejaarlijks overleg is met casuïstiek. Dat je van allebei de partijen casuïstiek waarvan je denkt: daar loopt het vast of daar loopt het niet goed. Dat je die erbij pakt en dan met elkaar gaat praten over hoe je het kunt verbeteren. Als je dat blijft doen en de communicatie openhoudt, dan werk je structureel naar verbetering van de toekomst. Dat is bij de gemeente ook aangegeven, dat is soms moeilijk want een wethouder heeft ook hele andere belangen. Maar ik heb het idee dat er vanuit het CJG welwillendheid is om daar aan mee te werken. En dan blijft de eeuwige discussie: wie moet dat gaan faciliteren. Laten we eerlijk zijn: een lunch met een paar broodjes, dat kost een godsvermogen maar dat is het. Meer moet je het ook niet maken.
***Nou het grappige is dat we in het kader van dit onderzoek dat ook gaan doen (uitleg focusgroepen)***
Maar weet je, ik denk dat daar de essentie zit. Focusgroepen dat klinkt weer zo zwaar, vind ik. Ik denk als je één keer per jaar met een aantal huisartsen en het CJG aan tafel gaat zitten. Je nodigt iedereen uit, en dan mogen mensen zelf kiezen of ze daar behoefte aan hebben of niet. Ik denk een beetje casuïstiekbespreking en je bespreekt de vernieuwing: vanuit het CJG vernieuwt er nogal wat. Dan zijn wij als huisartsen weer op de hoogte van wat zij weer doen, bij ons verandert er niet zoveel over het algemeen (tenzij we opeens een POH jeugd gaan krijgen, dan zou je dat moeten melden.) en je brengt wat casuïstiek in en je bespreekt: waar zitten onze raakvlakken en waar zitten onze knelpunten? Dan werk je structureel aan verbetering. Als je dat één of twee keer per jaar doet, dan denk ik dat je een heel eind komt.
***Dat is een waardevolle tip. Dus eigenlijk: wat wij nu een focusgroep noemen dat zou een soort therapeutisch effect kunnen hebben?***Nou kijk, een focusgroep dat zet je op om iets van de grond te krijgen. Wat ik zeg, als het goed is hebben al die gemeentes waar je heen gaat die hebben ook werkafspraken gemaakt - want dat moesten ze -. Dus in feite is er al heel veel overleg geweest, want die afspraken zijn echt niet zomaar van de grond gekomen. En dan denk ik: we kunnen blijven overleggen tot we een ons wegen. Maar dat is niet de oplossing, de oplossing is namelijk dat de praktijk beter moet werken. Dat ga je met praten alleen niet doen, alleen als je het doet. Dus tuurlijk, ik geloof echt in casuïstiekbespreking, want dan kom je concreet voor een probleem en dan kun je knooppunten waarvan je zegt: oke, dat gaan we verbeteren en dan na een jaar of evt. een half jaar evalueren. Maar alsjeblieft niet te vaak, ga niet om de 6 weken met elkaar aan tafel zitten. Daar ga je niet beter van worden. Het punt is, na 2 jaar is dat weer een probleem. -KORTE TUSSENSTOP- We waren bij de aanbevelingen. Ik denk: omdat we die voorbespreking al gehad hebben rondom de afspraken moeten we nu vooruit gaan werken. Dat lijkt me op deze manier een goede manier.
***Dat is ook zeker een aanbeveling... per gemeente merk ik ook wel verschillen natuurlijk - hoe die samenwerking verloopt, maar dat zou een goede eerste stap kunnen zijn natuurlijk.***Nou, misschien is het ook wel zo: het CJG werkt bij ons gewoon heel erg goed. Het is goed opgezet, het is een heldere structuur. Dat scheelt denk ook wel. Ik denk dat als je een minder goed functionerend structurerend CJG hebt, dat dat dan anders is.
***Dat waren de belangrijkste dingen die ik wilde bespreken.***EINDE INTERVIEW

**Interview Hpo3m**
Algemeen:
***Hoe lang bent u al huisarts?***22,5 jaar. Niet hier, maar vanaf mijn afstuderen.

***Hoe lang werkt u al in deze praktijk?***Dat is een lastige vraag. Ik ben in 1995 in <naam plaats> begonnen, ik ben officieel op 1 januari 1996 geassocieerd met een collega. In 2002 zijn we gedissocieerd want zij hield er mee op. En toen heb ik een HidHA genomen, toen ben ik samen met de HidHa verhuisd van de flat hiernaast naar het ziekenhuis, naar een ruimte. Toen ben ik een kostenmaatschap begonnen, eerst met een collega die daar al zat en later met de HidHA die derde maat is geworden. En toen zijn we nog even 8 maanden gekampeerd op de huisartsenpost en op 1 april 2007 (dus over 1,5 week bestaan wij exact 10 jaar).
***Oh? Gefeliciteerd!***Dank je. Toen zijn we begonnen met 7 maten. 10 jaar werk ik dus nu in deze praktijk.
***Dat is een geschiedenis, zeg maar.***
Ja, da's een geschiedenis. En we hebben in totaal 17.000 patiënten, waarbij patiënten opnamen van de huisartsen staan, althans voor de patiënt. Voor de verzekering zijn we één grote club, maar voor de patiënt staan voor opname (?) ingeschreven.
***En zijn dat alleen patiënten uit <naam plaats>?***
Nee, <naam plaats>. We hebben een enkele die maar niet weg wil, eentje in <naam plaats> en weet ik veel waar, die persé hier wil blijven en ik maar niet weg krijg. In principe hebben we alleen patiënten uit gemeente <naam gemeente>.
***Alvast een beetje toespitstend op het onderwerp waar we het zo over gaan hebben: hoe vaak komt het nou voor dat u op uw spreekuur gezinnen ziet die zich presenteren met psychosociale problematiek?***Een paar keer per maand.
***En wat voor problematiek is dat dan, bijvoorbeeld? Wat ziet u zoals?***Heel veel vragen omtrent gedragsproblematiek: ADHD, autisme, ''heeft mijn kind autisme'', ''heeft mijn kind ADHD''. ''Hij komt niet mee op school''; kinderen die gepest worden, kinderen die somber zijn, vaak wat oudere kinderen. Van alles, eigenlijk. Kinderen die moe zijn, waar psyche als probleem achter blijkt te zitten; kinderen die gedragsproblemen vertonen door andere problemen thuis, financiële problemen, relatieproblemen tussen de ouders - dat soort zaken. Heel af en toe uiteraard huiselijk geweld, danwel kindermishandeling maar dat komt een paar keer per jaar zou ik zeggen dat je dat meemaakt. Of dat je een melding krijgt vanuit een andere hoek. Nou, dat is het wel zo'n beetje. Heel divers.
***U had het al een beetje verteld per mail, dat u vaak ook contact heeft met de POH-GGZ als dit soort problematiek zich op het spreekuur presenteert.***
Ik verwijs vaak vrij snel door naar de POH-GGZ.

Casus 1 (Dave):
1. ***Wat is de eerste indruk die u krijgt als u deze casus leest?***Dat is een bekende vraag, vaak omdat school er dan aan denkt. Het eerste wat er in me opkomt is: ''is het alleen school die er aan denkt of denken ouders er ook aan?'' M.a.w. zijn er problemen op meerdere sociale domeinen?
***Dus de opinie van school en ouders, dat zijn de dingen die u graag...?***Ja, herkennen ouders het of niet? Thuis is 'ie prima, dan heeft die juf waarschijnlijk een probleem. Herkennen ouders het wel, dan gaan we verder kijken.
***Als ik het goed begrijp verheldert u de hulpvraag van ouders?***Ja, zijn ze gestuurd of kennen ze het probleem zelf ook?
***En komt dat vaak voor, dat school iets anders denkt dan ouders?***Dat komt wel eens voor, ja. Dat ouders zeggen, van mwja: nee hoor, thuis zit 'ie rustig te spelen. Hij heeft vriendjes waar hij prima mee kan spelen.

2. ***Als u dit zo leest: wat denkt u dat de oorzaak is van het probleem waarmee Dave nu komt?***Het grappige is dat hij thuis zijn schoolwerk binnen een paar minuten afmaakt, maar ze merken toch dat hij thuis ook wel wat problemen vertoont. In deze fase gaat iemand bij mij dan naar de POH-GGZ voor verdere inventarisatie of beoordeling.
***Wat van deze informatie is de trigger om te zeggen: dit stuur ik door?***Puur omdat ik zeg: nou, er is toch wel wat aan de hand. Ik denk dat het goed is dat de POH-GGZ kijkt of er mogelijk sprake is van ADHD en dan gaan ze het ADHD-trajectje in wat we hier hebben en dat betekent dat ze voor een diagnose naar een psycholoog gaan, en dan komen ze weer terug en dan doen wij de medicamenteuze behandeling. De POH-GGZ blijft ze dan begeleiden.

***Dus als ik het goed begrijp doet de POH-GGZ zowel diagnostiek als een stuk behandeling?***De echte diagnostiek wordt extern gedaan, dat moet echt door een psycholoog vinden we - dat doet de POH-GGZ niet. Maar als de diagnose gesteld is komen ze weer terug en dan doet de POH-GGZ verdere begeleiding. Ik ben er dan voor de medicamenteuze steun.

***We hadden het er in de mail over dat de POH-GGZ m.n. de contacten onderhoudt met JGT's, krijgt u van dat contact nog iets mee?***Ja, in die zin van dat we elke 6 weken overleg hebben met de POH-GGZ om patiënten door te spreken. En tussendoor krijg ik dan wat meer informatie om dat ik in het jeugdtraject zit, zeg maar. Ik praat in de gemeente over jeugdzorg. En één van de POH-GGZ ook, dus daar praten we wat meer over de JGT's en wat ik dan vaak hoor is dat als er contact is dat het dan vaak goed loopt, maar dat het contact er vaak niet is. Dat er wel contact is als ze vanuit hier naar het JGT verwijzen, maar wordt iemand rechtstreeks naar een JGT verwezen dan horen wij er eigenlijk nooit meer wat van.
***Dat is een interessant punt.***Dan zeg je het netjes, ik vind het bizar dat het zo is. Er wordt dan vaak geroepen: ''privacy''. En dan zeggen wij: privacy, allemaal leuk, maar vraag dan even aan de ouders of ze het goed vinden dat jullie informatie met ons delen, want misschien hebben wij ook wel informatie die nuttig voor jullie is. De ervaring leert dat 99% van de ouders het prima vindt. Als ouders geen toestemming geven, moet je je achter de oren krabben waarom dat zo is - denk ik dan.

***Want hoe zit dat voor de rest met de inhoudelijke terugkoppeling? Ik hoor u over een stukje privacy, maar stel er is terugkoppeling: hoe verloopt die dan (volledigheid van informatie e.d.)?***Niet. Het enige wat we af en toe te horen krijgen is - dat is nu twee keer gebeurt, in ieder geval als een soort trofee rondgedragen die e-mail, dat er iemand zich aangemeld had bij het JGT en of wij nog verdere informatie hadden. Dat is denk ik twee of drie keer gebeurd, daarna horen wij er nooit meer wat van.

***Wat vindt u daar van?***
Ik vind dat buitengewoon slechte zaak. Wij zijn als huisartsen voor alles zo'n beetje de poortwachter en de centrale dossierhouder. Op één of andere manier is de jeugdzorg daar een rare uitzondering op - en dat vind ik gewoon slecht. Wij kennen het gezin, al heel lang vaak, we hebben veel meer achtergrondinformatie en andersom: ik vind dat wij veel meer informatie moeten hebben omdat als die ouders dan weer hier komen en zeggen: ''we hebben een probleem'' - dan weer ik van niks. Ik moet van ouders soms horen dat kinderen in een heel ver traject zijn, terwijl wij nog niks weten. Dat vind ik gewoon niet goed.
***Misschien ook met het oog op dat medicamenteuze aspect.***Bijvoorbeeld, of als ouders problemen hebben die ik niet goed kan duiden maar die ik veel makkelijker had kunnen duiden als ik geweten had dat er met de kinderen dit en dit speelt. Ouders gaan er vanuit dat wij op de hoogte zijn, dus die zullen niet spontaan melden van: ''dit of dit is er aan de hand''. Iedereen gaat er altijd maar vanuit dat de huisarts alles weet.

***U noemt een aantal punten waarop het af en toe stroef loopt. Zijn er nou nog aanbevelingen of tips waarvan u zegt: zo zou het beter kunnen, bijvoorbeeld?***Wat mij betreft heel simpel: als iemand zich aanmeldt bij het JGT dan is één van de vragen die u stelt: vindt u het goed als wij informatie opvragen bij de huisarts en dat wij de huisarts op de hoogte houden van het vervolg? Punt. Het gros van de huisartsen zal dan ja zeggen en dan verwacht ik ook dat bij aanmelding en bij en ontslag een brief krijg - net als bij de specialist - en tussentijd als er bijzondere dingen zijn. Dat stelt mij in de gelegenheid, en die brief van: ''er is iemand bij ons aangekomen'' bevat dan ook de vraag: ''heeft u nog informatie waarvan u denkt dat het relevant voor u kan zijn? En laat het ons weten.'' Dat doen verloskundigen bijvoorbeeld ook, dat mensen rechtstreeks bij de verloskundigen komen en dat wij dan een brief krijgen: ''mevrouw is zwanger, als u nog informatie voor ons heeft: laat het ons even weten, omdat dat belangrijk is voor de zwangerschap.'' En ik krijg aan het eind van een brief als ze bevallen is, zo zou ik het van de JGT's net zo willen hebben.

3. ***Interessante punten, in ieder geval. Ik stel voor dat we naar het volgende punt (van de casus) gaan. Vóór dit punt zou een patiënt volgens u al bij de POH-GGZ zijn, maar leest u het maar.***Ik zie het. Waarom komt mevrouw bij mij, is de eerste vraag? Ze is onder behandeling toch? Dat is mijn eerste vraag, hier: wat wil ze van me?
***Klaarblijkelijk is er toch een reden om niet verder te gaan met het JGT, of ten dele verder te gaan met het JGT en dan toch uw hulp in te schakelen.***Ja, en dat is raar. Want in principe is het zo dat het JGT toegang heeft tot alle andere vormen van hulpverlening dus daar hebben ze mij niet voor nodig. Dan zal moeder wel iets willen wat het JGT niet kan bieden of moeder verwacht iets van het JGT dat niet komt, of er zijn communicatieproblemen of - whatever.
***Zijn er nog dingen die u graag zou willen toevoegen aan de hand van deze casus? Dingen m.b.t. tot het JGT waarvan u zegt: dat is onbesproken gelaten?***Het meeste heb ik wel besproken. Ik vind het JGT an sich prima, het is een goede manier om te zorgen dat niet 6 verschillende hulpverleners op een gezin zitten die niet weten van elkaar wat ze... Die gedachte is uitstekend, maar a) vind ik dus dat de huisarts daar bij hoort, wat ik lastig weer van het JGT vindt is dat er ook mensen zitten die er belang hebben bij een vervolg. Ik zou heel graag intake en behandeling scheiden en daar gaat ook onze propositie over: in principe dat elk kind dat zich aanmeldt eerst door ons beoordeeld wordt en dan pas wordt voorgeleid in de rest: dat kan naar het JGT zijn als er sprake is van multiproblematiek maar dat kan ook naar een eerstelijns psycholoog zijn, dat kan rechtstreeks naar de tweedelijns GGZ zijn als er echt ernstige problematiek is. Kortom: dat wij weer net als voor alle andere dingen in gezondheidsland de poortwachter worden.
***In die zin zouden jullie wel graag de spin in het web willen zijn, zeg maar?***Ja, dat wil niet zeggen dat ik me nou overal mee zou moeten bemoeien, he. Het kan ook zijn dat ik zeg: nou dit is een gezin waarin kinderen gedragsproblemen hebben, ouders financiele problemen hebben, er zijn relatieproblemen - dus weet ik veel wat allemaal. Ja, dan pretendeer ik niet dat ik dat allemaal in de klauwen kan houden, maar dan ben ik wel degene die iemand naar het JGT verwijst met: ''dit is voor jullie''. Ik blijf er wel graag bij betrokken, maar niet in de zin van: de coördinator. Deze problematiek is zo divers, er moeten zoveel domeinen in - dat laat ik graag aan een ander over. Of de POH-GGZ zegt: ''dat pak ik op''. Hangt ook een beetje van de problematiek af en wat er voorop staat.
***Wat ik me verder nog afvroeg, aan de hand van wat u verteld hebt, dat bepaalde binnen het JGT er voordeel.***Ja, voordeel klinkt wat onaardig - net alsof ze er voor hun eigen broodwinning zitten - maar het is gewoon een gegeven dat als jij daar zit en je moet beoordelen of iemand een behandeling behoeft en jij kunt zelf die behandeling bieden... Ja, dat geldt voor ons ook hè, net zo goed. Als wij er belang bij zouden hebben, dan speelt dat toch een rol. Niet zozeer bewust, ''ik moet volgende week op vakantie, dus ik moet zorgen dat ik flink wat behandelingen doe'', zo werkt het niet. Maar, het zou gezonder zijn om dat te scheiden.

***Interessante punten, wederom. Dan stel ik voor om naar de tweede casus toe te gaan.***
Casus 2 (Sanne):
1. 2. en 3.
***Is dit een herkenbare casus?***Jawel, het eerste wat bij me opkomt is een kind met een stoornis in het autisme-spectrum, zoals dat heet. En verder: multiproblematiek, zieke moeder, financiële problemen, zorgen, geen schulden - het komt wel vaker voor dat er schulden zijn, in dit soort situaties -. Ja, ook hiervoor geldt dat ik al na de eerste ontmoeting de POH-GGZ ingeschakeld zou hebben en, gelet op het vervolg, zal die ongetwijfeld contact met school hebben opgenomen. En, bij de derde denk ik dat de JGT ingeschakeld zou worden, dat is een beetje het verloop wat ik voor me zou zien op basis van deze casus.
***Is dat ook iets wat u dan bespreekt met de POH-GGZ, het plan van aanpakt wat u nu zelf bedenkt?***Nee, als ik de eerste keer verwijs dan verwacht ik dat bij de eerste bespreking met de POH-GGZ waarschijnlijk al overlegd is met school en dat ik dan te horen krijg: ''ik heb overlegd met school en dit en dit komt er uit, mijn plan is dat en dat''. Ik hoef daar niet eens mijn viat (?) op te geven, onze POH-GGZ is dusdanig geschoold dat zij grote mate van zelfstandigheid hebben.
***Want de POH-GGZ in deze praktijk, hebben die nog aanvullende opleidingen gevolgd?***De één is een oud-SPV'ers van <naam specialistische GGZ-instelling> met heel veel ervaring, de ander is psycholoog en de derde - weet ik even niet, we hebben pas een nieuwe dus ik weet niet wat haar achtergrond is -, maar die werkt bij <naam specialistische GGZ-instelling> (parttime). Dus mensen met zeer brede ervaring en ook interesses. We hebben een buitengewoon goed draaiend POH-GGZteam dat gewoon heel veel zelf doen en ook zelf met initiatieven komen. Heel veel zelf regelen, maar het wel afstemmen met ons.
***U noemt al een kernwoord: multiproblematiek met betrekking tot deze casus. Waarin verschilt volgens u deze casus van de eerste casus?***Met name in de problemen van de anderen, zal ik maar even zeggen. Want de eerste casus zie je gewoon dat het vooral het kind zelf is. Je ziet verder geen dingen - althans die staan niet beschreven - dat er problemen in het gezin zijn, relatieproblemen, financiele problemen etcetera. En dat is hier wel het geval. Het is ook niet het enige kind met problemen in de zin van: ze zijn allemaal wat lager intelligent. Ik lees niet dat andere broers of zussen deze problemen vertonen. Maar wel: financiële zorgen, moeder die ziek is, ouders die hetn iet altijd met elkaar eens zijn.
***Want voordat u door zou verwijzen naar de POH-GGZ, zou u bijvoorbeeld nog die andere kinderen op het spreekuur uitnodigen?***
Nee, dat laat ik aan de POH-GGZ of die daar het nut van inziet. Meestal ga ik er vanuit, dat gezien het verhaal, dit niet de eerste keer is dat ik ze zie. Ik verwacht dat ik al wat meer kennis van het gezin heb.

***Duidelijk verhaal, wederom. Dan stel ik voor dat we naar de derde casus gaan.***
Casus 3 (Melany):
1. 2. en 3.
*lacht* Ik begin een beetje te lachen, je hebt inderdaad wel casussen genomen die redelijk veel voorkomen.
***Daar zijn ze op geselecteerd, inderdaad.***Hmm. Raar. Nou, ik vind het beloop raar in de zin van: eerst komt moeder alleen, dan samen zonder Melany en dan komt Melany met beide ouders. Ik zou na casus 1 eerst gevraagd hebben of moeder met Melany komt. Of vader en moeder met Melany, maar ik zou het volgende gesprek eerst met Melany willen doen.
***Het is dus meerde volgorde van presenteren op het spreekuur...***Ja. Ik vind het onlogisch dat eerst moeder het verhaal komt doen en dan komen beide ouders - die zijn gescheiden. Ik zal waarschijnlijk allang weten dat het niet zo botert tussen die twee. Dan vind ik het meer waarde.. kijk ze zullen allebei zeggenschap hebben (dat vind ik een probleem, hoor, daar lopen we vaak heel erg tegen op dat beide zeggenschap hebben over hun kinderen en dan wil de één iets voor het kind en dan wil de ander dat niet en vaak wordt dan het kind gebruikt als middel om de ander flink dwars te zitten. Ik heb daar wel 's het <naam advies- en meldpunt voor huiselijk geweld en kindermishandeling> voor gebeld, dat heet dan tegenwoordig <naam advies- en meldpunt voor huiselijk geweld en kindermishandeling>, omdat ik dat eignelijk een kind een vorm van kindermishandeling vind als het kind gebruikt wordt om de ruzie tussen ouders nog 's flink op te stoken ofwel te beslechten of wat dan ook.) In dit geval zou ik dus tegen moeder gezegd hebben: ''ik wil die Melany eens spreken, het liefst alleen eigenlijk. Zonder beide ouders.'' Want dan komt het verhaal meestal naar boven, als ze mij ten minste vertrouwt, meestal lukt dat wel. Dan weet ik een beetje hoe zij haar gedrag labelt, zal ik maar even zeggen.
***En wat voor dingen zou u haar willen vragen?***Nou, eigenlijk zou ik zeggen: ''je moeder is hier geweest, die maakt zich zorgen, ik begrijp dat het niet zo goed gaat op school en dat je regelmatig veel drinkt en zelfs dronken bent. Hoe komt dat, denk je zelf?'' Dat is de vraag. Er kunnen een paar dingen gezegd worden als: dat is heel normaal, dat doet iedereen - beetje pubergedrag. Of, en dan vraag je inderdaad wel door, er komt uit dat ze niet zo gelukkig is met de situatie.
***En stel: we hebben het hier over een scheiding die is geweest en dat ouders toch behoorlijk kunnen botsen.***
Ja, moeder vindt de boze buien van vader een reden voor de echtscheiding. Ja, ik zie dat gesprek al helemaal voor me.
***En stel: vader denkt heel anders over een doorverwijzing naar de POH-GGZ dan moeder, bijvoorbeeld. Wat zou je doen op zo'n moment?***Ja, dat is lastig. Ik vind een doorverwijzing naar de POH-GGZ nauwelijks iets waar ik toestemming voor nodig heb, eerlijk gezegd, omdat het nog binnen de huisartsenzorg valt. Ik vind het anders als ik haar naar de psycholoog of waar dan ook naartoe zou verwijzen. Maar goed, als ze daar een punt van maken probeer ik aan beide ouders duidelijk te maken dat ik het erg belangrijk vind en dat ik er vanuit ga dat ze toch het belang van Melany voorop stellen. En dat ik het in haar belang het belangrijkste vind dat ze goed geholpen wordt en dat we het probleem gewoon goed op een rijtje moeten krijgen. En vooral benadrukken dat ik geenszins partij kies of ieman de schuld geef, het gaat mij om het belang van Melany. Ik heb dus echt wel 's ruzie gemaakt met ouders, dat één van de ouders echt dwars blijft liggen terwijl ik echt vind... van nou, ja ''dien maar een klacht in, zij gaat nu daar heen'' - en dan gebeurt er nooit wat. Maar het belang van het kind staat voorop. Dat weeg je altijd af en als er echt iets moet gebeuren waarvan je vindt dat dat belangrijk is en één van de ouders houdt dat tegen, dan vette pech.
***Met betrekking tot zorg voor ouders, denkt u daar nog aan op dit moment?***Hier niet zo, eerlijk gezegd. Het is anders dan in deze casus *laat casus 1 zien*, dan komt al vaak het JGT om de hoek zeilen, want hier moet er ook iets met moeder.. bijvoorbeeld. Maar hier zie ik gewoon twee ouders die.. Nou het hangt natuurlijk een beetje van het verloop af, ligt dit meer in het kind of zit er een stuk opvoedingsproblematiek bij? Dan moeten de ouders daarbij ondersteund worden, maar als ik het zo lees dan denk ik: ''dit is vooral een punt van een kind dat moeite heeft met de scheiding van haar ouders en misschien zit er nog wel wat anders onder - als ik dat laatste verhaaltje lees.'' Nou, dan zal ik toch me voornamelijk op het kind concentreren.

***Ik zit nog even te spieken of er nog dingen zijn van mijn vragenlijst die ik had willen stellen m.b.t. deze casus. Ik geloof het niet. Ja, wat ik me nog wel afvroeg: we hadden het na casus 1 nog over de samenwerking met JGT en de knelpunten die u daarin merkt. Hoe zit dat met specialistische zorg, bijvoorbeeld <naam specialistische GGZ-instelling> in <naam plaats>?***Dat liep over het algemeen wel goed, maar doordat de aanbesteding is gaan spelen is er heel veel onrust onstaan bij de specialistische GGZ van: krijgen wij wel een contract en daar zijn dus heel veel goeie mensen vertrokken. Er zijn nog wel een aantal goeie mensen over. Maar een wachttijd van een half jaar bij de specialistische GGZ, dat is eigenlijk niet acceptabel. Dus wat gebeurt er dan? Dan ga je altijd bellen of het eerder mag, nou dat kan dan altijd wel en zeker als je goeie motivatie hebt van waarom dat eerder zou moeten. Maar die samenwerking is beter (als meer informatie-uitwisseling) dan met het JGT. Hetzelfde geldt voor, waar we heel veel mee samenwerken, is eerstelijnspsychologen en orthopedagogen. Dat werkt ook prima, dat is een clubje waarin wij vertrouwen hebben - waarvan we weten dat die goed werken en we weten ook precies van: voor dat probleem kun je beter hier heen en voor dat probleem kun je beter daar heen. Het vervelende is: die zaten dus niet in de aanbesteding, dus wij zouden weer met hele nieuwe zorgverleners moeten gaan werken. En als er iets vaags, je zou die aanbesteding 's moeten bestuderen - dat is een kunststukje op zich,- maar ze wilden dus één partij maar er was dan nog een *vrije schil*, zoals dat dan heette, ik weet niet hoeveel procent van het budget. En daarin zou die zorgaanbieder, dus niet wij, maar die zorgaanbieder mogen bepalen om ook nog anderen een stukje te gunnen. Wij hoopten natuurlijk dat dat het clubje was waar wij regelmatig mee samenwerken, maar ik zou het vreselijk vinden om met een compleet nieuwe organisatie te gaan samenwerken. Je zit met een stuk continuïteit, want vaak zit je met trajecten van jaren. En ze hebben aangegeven van: hooguit nog een jaar dat je nog die andere club contracteert en dan is het op. Dan denk ik van: ''dat is rampzalig voor iemand bij wie de hulpverlener nou goed loopt, om dan weer met hele nieuwe zorgverleners te komen.'' Ik snap wel dat je niet tot in het oneindige een club iets kunt gunnen zonder dat je kunt ingrijpen op kwaliteit of kosten, of wat dan ook. Maar dit werkt ook niet echt.
Maar goed, je zei iets over samenwerking.
Over het algemeen loopt dat dus wel goed, zeker als je de mensen al wat langer kent en weet wat je aan mekaar hebt.
***Wat zou uw voorkeur nou hebben, als u doorverwijst.. wat zou de ideale samenwerking inhouden, laat ik het zo vragen?***Ideale samenwerking betekent voor mij dat je over en weer laagdrempelig bereikbaar bent. Ik moet weten wie ik kan bellen voor welk gezin. Ik wil weten: wie is de behandelaar? Ik wil bericht hebben van: ze zijn hier aangekomen en we hebben dit en dit geconstateerd en we gaan dit en dit doen. Ik wil bericht hebben: we hebben dit en dit allemaal gedaan, dit is het resultaat en we sluiten de casus af. Ik wil er graag bij betrokken worden, danwel mijn POH-GGZ, als er multidisciplinair overleg is waarin wij ook een rol kunnen spelen. Ik hoef echt niet overal bij te zitten, daar heb ik geen tijd voor. Maar soms is het heel zinnig als je 's met een groepje overlegt: hoe gaan we nou met dit gezin verder? Nou, in die zin. En dat ik uiteindelijk vrij ben om te bepalen waar ik mensen naartoe verwijs Ik wil daar niet in geremd worden door een gemeente die - met alle respect - toch geen verstand van zaken heeft, in die zin: geen medisch verstand van zaken. Dat vind ik in gewikkeld om daardoor beperkt te worden. Die beperking heb ik namelijk nergens anders. Ik kan naar elke specialist verwijzen, ook als er geen contract is tussen de zorgverzekeraar en de betreffende specialist, kan ik nog steeds met de patiënt overleggen: ''hoor 's je moet 30% zelf betalen of toch vind ik dit het beste voor je. Kun je het betalen of niet?'' Maar hier is het gewoon van: je kunt daar naartoe verwijzen, of anders moeten ze alles zelf betalen. Ja, er zijn maar weinig mensen die een psycholoogbehandeling van A tot Z kunnen betalen.
***Dat speelt ook een rol. Ik denk dat ik in dit half uur een hele hoop wijzer ben geworden, een aantal interessante punten wijzer ben geworden. Zijn er nog dingen die onbesproken zijn gebleven? De bedoeling van dit onderzoek is dat ik er uiteindelijk een wetenschappelijk artikel van maak en met een beetje geluk dat het ook internationaal gepubliceerd wordt.***
Dat wil ik graag hebben, als het gepubliceerd wordt.
***Ja, dat is inderdaad het volgende wat ik wilde zeggen.***
Nee, volgens mij hebben we de meeste dingen wel besproken eigenlijk. Nog wel een aantekening: wat ik wel merk is dat niet alle huisartsen op dezelfde manier er in zitten: er zijn voldoende huisartsen die het prima vinden dat er een JGT is waar ze gewoon al die problematiek naar toe kunnen schuiven, dat ze er zelf lekker geen last van hebben. Ik denk dat je het zo kunt organiseren dat je er geen last van hebt - ik vind het woord last al verkeerd, want volgens mij is het gewoon onderdeel van je vak - maar dat je POH-GGZ zo positioneert dat die gewoon het werk doet. Het enige wat je als huisarts hoeft te doen is daar af en toe 's mee te overleggen zoals je dat ook - ongeacht met welke andere specialisme ook met je POH (somatiek) doet - of dat je met POH-GGZ over volwassenenzorg. Het is echt niet zo dat ik opper dat huisartsen zich intensief moeten gaan bemoeien met de jeugdzorg, helemaal niet want we hebben daar geen tijd voor (daar zijn we ook niet voor ge-ecipeerd (?)), maar wij zijn wel goed ge-ecipeerd (?) voor eigenlijk een soort triage van: wie kunnen wij zelf behappen en wie moet doorverwezen worden? En daarmee ook een belangrijke kostenbesparende factor kunnen zijn.
***Dat is een punt dat ik zeker mee ga nemen. Het wetenschappelijk artikel zal ik uiteraard doorsturen naar de deelnemende huisartsen.***

EINDE INTERVIEW

**Interview Hxfz6**Algemeen:
***Hoe lang bent u al huisarts in totaal?***Even denken, bijna 8 jaar.
***Hoe lang werkt u specifiek in deze praktijk?***Dat is dan 3 jaar.
***En wat heeft u daarvoor gedaan?***In <naam plaats> heb ik toen gewerkt als HidHa, dus dat is bijna 5 jaar.
***Alvast een beetje toespitsend op het onderwerp waar we het zometeen over gaan hebben: hoe vaak komt het nou voor dat er op het spreekuur een kind of jongere komt, waarvan u zegt: daar zou iets op psychisch of sociaal gebied verbeterd kunnen worden?***Dat vind ik al een lastige vraag, want er komt vaker de vraag vanuit ouders van: wij hebben zorgen of er loopt iets niet helemaal goed rondom het kind (op school) of zorgen rondom het gedrag of zoiets. De keren dat wij denken: er moet meer zorg gebeuren dat is - hoe vaak zou dat zijn? - het is maar bij een beperkt aantal gezinnen dat wij zelf die zorg zo hebben zonder dat de zorgvraag vanuit het gezin komt. Dus dat is.. één keer per maand is al veel denk ik.
***Zijn dat alleen maar nieuwe gevallen of zijn dat dan ook follow-upgesprekken?***Ja dat zijn ook gezinnen waarvan je weet dat er in het verleden al wel zorg geweest is of dat er eerder zorg geweest is en dat er dan terugekerend dat je denkt van: loopt dat wel helemaal lekker?
***Werkt hier voor de rest een POH-GGZ in de praktijk?***Ja, maar die doet geen kinderen.

Casus 1 (Dave):
1. Ja.
***Wat is de eerste indruk die u krijgt als u dit zo leest?***Dit is een vraag die vrij regelmatig voorkomt. Ja, dan heb ik eerst een heleboel vragen over waar de zorg vandaan komt: is dat vanuit school of vanuit ouders en wat er dan precies gaande is. Als ik dit van tevoren al weet, als het goed is er al wat getrieerd en dan zie ik in de agenda waar mensen voor komen. Dan kijk ik wel even in het dossier of er eerder al zorgen zijn geweest of zijn er in het gezin meer kinderen met problemen - dus dat kijk dan wel even na.
***Zo komt u aan achtergrondinformatie en zo gaat u het gesprek in?***Ja.
***U noemde zojuist dat u wat vragen wilde stellen. Wat zou de eerste vraag zijn die nu in uw hoofd opkomt?***Waar ze een probleem mee hebben, want school denkt aan autisme en ADHD -zie ik-, of ouders dat zelf dan ook denken. Of ze thuis zelf ook problemen hebben, of er op school al iets mee gedaan is. Of ze al maatregelen ervoor hebben genomen, of het kind echt slechte prestaties heeft of dat het echt het gedrag is waar ze last van hebben.
***Ophelderende vragen dus?***
Ja.
***Oké, dan stel ik voor dat we naar het tweede gedeelte gaan. Dan komt er ook wat meer informatie beschikbaar.***

2. ***Ze zijn leuk geschreven dit casussen, hè?***Haha, ja.
***Hoe vormt deze informatie uw beeld van wat het probleem zou kunnen zijn?***Nou, een paar dingen.. thuis, wat ze zien en op school wat er gebeurt en dat het in de familie voorkomt en ook dat ouders dus al wel een beetje hebben gekeken of tenminste zelf een mening hebben: het zit hem niet in dat hij niet wil luisteren of dat het gewoon niet lukt. Maar er zijn ook wat tegenstrijdige dingen. Thuis heeft hij zijn schoolwerk in een paar minuten af, dus dat is dan weer gek dat hij dat doet. Ja, dus er komt wel wat informatie naar voren denk ik. Er staat nog niet echt een duidelijke vraag van ouders.
***Ja, en ik proef daaruit dat u dat dan zou willen vragen?***Ja.
***Wat is het eerste dat u zou doen als u deze informatie op tafel heeft?***Nou, eerst de situatie wat helder krijgen - dus met die aanvullende informatie - en dan met ouders verkennen wat ze willen: willen ze diagnostiek en op welke manier zouden ze dat willen? En dus ook: waar komt de vraag vandaan? Is dat echt vanuit school gekomen of is dat echt vanuit ouders zelf? Dus dan verkennen wat voor hulp ze daarvoor zouden willen hebben.
***Dan stel ik voor dat we naar nummertje drie gaan, daar wordt een stukje van het beleid bekend. Het JGT wordt genoemd, is dat een instantie die u bekend in de oren klinkt?***Ja.
***En is dat ook iets waar u nu aan zou denken bij deze jongen?***Jazeker, dat is iets wat ik met ouders zou bespreken. Nu is het JGT er kennelijk al bij betrokken, als dat niet zo is dan is het wel zo dat ik kijk of het eerst naartoe zou kunnen. En dat hangt er dan een beetje van af hoe specifiek de vraag voor diagnostiek is. Is dat heel specifiek, dan is dat een beetje een omweg om eerst naar het JGT te gaan en dan gaan die weer doorverwijzen voor diagnostiek. Ja, we hebben pas een overleg gehad tussen JGT en gemeente. Aan de ene kant wil de gemeente dat alles via het JGT loopt, vanwege de financiële stromen zo beperkt mogelijk blijven. Aan de andere kant zegt het JGT: wij komen om in het werk. Als jullie duidelijk weten wat de vraag is dan kunnen jullie best direct verwijzen. Dus daar zitten we een beetje van: ik verwijs nu wel direct als ik denk dat dit een duidelijke vraag is voor diagnostiek (dat doet het JGT niet zelf, daar verwijzen zij ook voor). We hebben af en toe wel 's contact en dan hoor je via het JGT: je kunt het beste daar naartoe verwijzen want daar zijn de wachttijden het kortst bijvoorbeeld. Dus het wil nog wel 's n beetje in overleg met het JGT.
***Oké, met name diagnostiek zou één van de redenen zijn om het JGT mee te laten kijken in dit geval. Hoe lang zit het JGT al specifiek in deze gemeente?***Ja, ze zijn begonnen met een proeftuin. Dus ik denk dat dit het derde jaar is dat ze bestaan.
***U noemde al dat u dan overlegt van: wat moeten we doen met een bepaald kind? Hoe vindt u eigenlijk dat dat gaat ook wat betreft de samenwerking met het JGT?***
Het overleg gaat altijd wel goed, vind ik. Er is één nummer waar altijd iemand bereikbaar is. En we hebben twee personen voor de huisartsen die we kunnen bellen. Dus dat gaat eigenlijk altijd goed.
***Dus jullie hebben echt vaste contactpersonen, zeg maar?***Ja.
***En die persoon weet dan ook, als u het over een specifiek kind of jongere hebt, dan weet die contactpersoon waar het over gaat? Hoe gaat zoiets?***Volgens mij hebben ze wel hun eigen case load, als ze dat zo noemen, dus ze weten niet meteen wie dat is als het niet diegene is die zij zelf zien. Maar eigenlijk is het heel vaak zo, het is niet zo'n heel groot team. Dus als je het over een kind hebt dan weten ze vaak wel waar het over gaat.
***De mensen die in het JGT werken, kent u die ook persoonlijk? Zijn de namen daarvan bekend?***De contactpersonen ken ik, ik weet wie het zijn. En de anderen kom ik wel 's tegen of zo, maar die ken ik verder niet.
***Oké. Wat dat betreft het JGT. Stel ik voor dat we naar de tweede casus gaan.***
Casus 2 (Sanne):
1. 2. en 3. tezamen.
Hmm, oké.
***Kunt u in één of enkele kernwoorden benoemen waar voor u het grote verschil zitten t.o.v. de eerste casus?***De benedengemiddelde intelligentie is denk ik belangrijk hierin. De sociale context want wat je hier ziet: klein behuisd, financiële zorgen, dat kwam de vorige casus helmaal niet aan bod. Dat wordt hier zo benoemd dat dat wel belangrijk is. En als ik het zo zie is er verder zo zie is er nog geen hulp. Dan denk ik wel: er is een gezin met vijf kinderen waarvan een broertje met lagere intelligentie is - dat ik me afvraag: wat rommelt daar allemaal in dat gezin?
***Dus, de familie-anamnese?***Ja.
***Oké, want u noemde al zojuist een laag IQ. Zijn er nog andere diagnosen die in uw hoofd opploppen als u dit zo leest?***Oh, diagnoses heb ik nog niet echt zo... maar Sanne is erg gestructureerd dus dat heeft iets van autisme weg als het allemaal volgens bepaalde rituelen moet. Ja, dat denk ik. Er is niet een andere diagnose die ik er op zou plakken.
***Haha, nee dat kan. Wat redelijk bijzonder is aan deze casus - die last heeft van osteopenie -. Hoe gaat u mee om in de spreekkamer? Moeder komt met Sanne en legt het probleem uit. Hoe zou u daarmee omgaan?***Nou, ze komen omdat school zegt: moeder houdt zich niet aan de afspraken. Het gaat nu niet over de ziekte van moeder denk ik, maar wel misschien over haar draagkracht. Dat komt later in het verhaal want ik zou eerst ingaan op het gesprek: wat speelt er allemaal en wat gaat er allemaal niet goed? En dan van hoe het voor haar is om daar mee om te gaan. Zeker als ik dit zie, met vijf kinderen, en er zijn ook wel meer kinderen met wat problemen. Of ze hulp hebben en hoe zij dat allemaal in haar eentje doet met haar lichamelijke klachten erbij. Dus dat zou ik wel even peilen.
***Oké. En wat is het eerste plan van aanpak dat u zou inzetten?***Dit vind ik ook wel iets voor het JGT, denk ik. Omdat ik denk dat hier juist heel breed in het gezin gekeken moet worden en dat is iets wat het JGT heel goed kan doen. En eventueel zou je nog met school contact kunnen hebben. Maar dat vind ik zelf altijd een beetje lastig, ik heb liever dat dan via het JGT loopt. Die hebben daar ook een contactpersoon, tenminste elk kind heeft één contactpersoon van het JGT dus dan heb ik liever dat dat via die kant loopt.
***Dus als ik het goed begrijp: als huisarts zoekt u niet zelf zo snel contact met school?***
Soms wel hoor, als je informatie vanuit ouders niet duidelijk krijgt. Dan vraag ik aan ouders of het mag dat ik er even overleg over heb. En er zijn ook wel 's leerkrachten die heel graag hun informatie willen geven en die zelf hun zorgen hebben. Dat is ook wel iets wat je dan weer meeneemt, van: is het gedragsmatig of is het sociaal of dat je iets breder kan kijken waar het in zit.
***Oké, en misschien ook wel vanwege het multidisciplinaire aspect van het JGT?***Hmm-hmm.
***Oké, dat waren de belangrijkste vragen die ik over deze casus wilde stellen. Gaan we alweer naar de laatste casus.***

Casus 3 (Melany):
1.2. en 3. tezamen
Hmm, ze is 15. Het zijn al drie contacten, eerst alleen moeder dan de ouders en dan met het meisje samen.
***Een heel andere casus dus, is dit overigens iets wat vaker voorkomt - deze vraag?***Zoals dit, nee.
***Casus 1 dat is...?***Dat komt best frequent voor, casus 2 is - wat ik zei - iets wat je dan één keer per maand ziet: zo'n soort situatie of ouders met verslavingsproblematiek of sociale problematiek of zoiets. Waarvan je denkt: ik weet niet of dat helemaal goed gaat met de kinderen. En dan zijn er ook nog wel 's wat zorgen op school waardoor het allemaal gaat rollen, zeg maar. Echt zo'n situatie. Ja, of wij wonen in een hele brave gemeente - haha.
***Haha, ja dat zou kunnen.***Maar volgens mij is dat helemaal niet zo hoor, in de <naam regio> wordt heel veel gedronken.
***Het is wel grappig inderdaad, ik kom in verschillende gemeenten en je hoort per gemeente verschillende prevalenties: de ene casus komt dan meer voor dan de andere. Dat is leuk om te zien. Eigenlijk had ik hier dezelfde vraag t.o.v. casus 2: wat zijn de kernverschillen t.o.v. de andere twee casus?***Nou, hier speelt scheidingsproblematiek en dit meisje is 15 (die andere waren jonger toch?). Drankgebruik en hier speelt ook weer dat school een mening heeft hierover.
***Wat zou uw plan van aanpak zijn?***Nou dit is wel lastig, want als moeder alleen komt dan heb je het over de zorgen van moeder en dan kan je eigenlijk verder niet zoveel. En met ouders ook: ja een meisje van 15 die moet je zelf daarbij betrekken. Dus eigenlijk pas in het laatste geval kan je samen met haar kijken wat je nodig hebt. En dit is een meisje waarvan ik denk: het zou waardevol kunnen zijn om haar alleen te spreken zodat je met haar kan kijken: wat zijn de problemen rondom die scheiding? Kun je daar iets in doen? En dan het drankgebruik, opstandigheid, die dingen en dat anderen zeggen dat ze geen gevoel heeft. Nou ja, dat eigen beeld van haar wat je dan op dat moment..
***En dat alleen spreken van Melany, is dat iets wat u dan zelf zou doen of gaat dat dan wel via de POH-GGZ?***Dat doe ik dan zelf, inderdaad. Deze leeftijd is.. nou het zou misschien wel kunnen, hoor met de POH-GGZ. Maar degene die we hebben die werkt hier nog niet zo heel lang dus daar zitten we nu nog niet zo van dat kinderen daar naartoe gaan. Maar als ik zou denken van: dat zou ik wel prettig vinden. Dan zou ik dat eerst daar even met hem bespreken en kijken van: goh zie jij dat zitten? En dan zou dat misschien wel kunnen. In eerste instantie zou ik dat zelf doen.
***En wat zijn de dingen die u met Melany zou willen bespreken?***Nou, rondom de scheiding: hoeveel last ze daarvan heeft. Wat er geregeld is en hoe zij dan dat eigenlijk zou willen zien en wat er in haar ogen zou moeten veranderen. Dat drankgebruik zou ik met haar zelf willen bespreken hoe dat in elkaar zit en hoe dat op school gaat en van: hoe depressief is ze? Gaat het die kant op, hoe ver gaat dat?
***En wat zijn de mogelijkheden daarna, bijvoorbeeld. Stel: u denkt aan depressiviteit bij Melany? Wat zou dan een vervolgstap zijn?***Met deze leeftijd (?) vind ik het altijd een beetje lastig, het zou misschien iets zijn om haar naar de GGZ door te verwijzen. Maar dat zit niet hier in <naam gemeente> dus dat is dan iets om met ouders te bespreken om te kijken: wat zou een goede plek zijn? Omdat hier meer speelt, zou ik ook dit met het JGT overleggen. Dus het is niet dat ik ze allemaal daar naartoe verwijs, maar dat ik toch wel eventjes overleg heb.
***Dus als ik het goed begrijp, komt bij alle casussen het JGT als één van de eerste opties in het hoofd op?***
Ja, alleen die eerste als het echt specifiek om diagnostiek gaat, dan verwijs ik niet naar JGT omdat ik weet dat zij zelf ook toch doorverwijzen dus dan doe ik het zelf.
***U snijdt interessante punten aan, in ieder geval, dus het is allemaal hartstikke bruikbaar. We zijn in principe door de casuïstiek heen. Ik had nog wel een aantal aanvullende vraagjes. -uitleg wetenschappelijk artikel en streven naar aanbevelingen en vraag om tips-.***
Nou, waar wij vooral tegenaan lopen, ik vind het JGT best goed bereikbaar. Je kunt met ze overleggen en de wachttijden zijn nu, geloof ik, wel weer wat minder - het liep even op maar ik geloof dat het nu wat minder is. Wat we wel merken, echt die zorgelijke situaties, dat er niet zoveel actie dan ondernomen wordt. Waarvan je vroeger eigenlijk <naam advies- en meldpunt voor huiselijk geweld en kindermishandeling> zou inschakelen, dat <naam advies- en meldpunt voor huiselijk geweld en kindermishandeling> nu dan vaak zegt: het JGT is erbij betrokken, dus wij doen niets. Maar het JGT doet eigenlijk ook niet zoveel, dus dan gebeurt er niet zoveel. Dat hebben wij nu twee keer gehad, dat wij dachten: hier moet nu wel iets gebeuren en dan zijn ze niet zo doortastend daarin. Dus, daar zitten we dan een beetje: het ligt wel daar, maar het is niet heel duidelijk dat ze dan de regie nemen.
***Dus de snelheid en duidelijkheid van handelen in acute situaties, daar schort het dan nog wel aan?***Ja, maar ik denk dat het een persoonlijk ding is van degenen die daar werken. Dat zij heel geneigd zijn om mee te gaan met ouders en te laveren en niet in te grijpen, en op een gegeven moment moet je echt ingrijpen.
***En dat is iets wat <naam advies- en meldpunt voor huiselijk geweld en kindermishandeling> dan wel zou doen?***Die doen dat wel wat sneller, ja. Maar je merkt nu, dat hoe het nu geregeld is, dat zolang JGT daarbij betrokken is en ouders daarin meewerken doen we niets.
***Dat is inderdaad een goed punt. Ik vraag me dan af: hoe zou dat verbeterd kunnen worden? Heeft u daar ideeën over? Wat zou het JGT anders kunnen doen om dan toch die actiesnelheid te verbeteren?***Ja, ik vraag me af of het niet ook tussen JGT en <naam advies- en meldpunt voor huiselijk geweld en kindermishandeling>.. of daar niet iets meer verbinding zou moeten zijn. Of het dan toch overdragen of van hun iets meer informatie krijgen of inzichten in hoe ze dat dan beter aan kunnen pakken. Ik weet ook niet zo goed hoe dat allemaal in z'n werk gaat hoor, als <naam advies- en meldpunt voor huiselijk geweld en kindermishandeling> dat doet maar het is wel zo dat voorheen - als je daar contact mee had dan werd je gebeld en dan kwamen ze bij ons informatie vragen - en van het JGT wordt er weinig naar ons teruggekoppeld, dus dat blijft altijd een beetje aan de oppervlakte heb ik het idee. Dus je moet er zelf echt achteraan om iets actievere houding..
***Oké, dus als ik het goed begrijp is er een wat gebrekkige terugkoppeling?***Ja, dat sowieso maar dat is vanaf het begin al zo.
***En waarin uit zich dat? Bijvoorbeeld in schriftelijke communicatie?***Ja, die krijgen we eigenlijk niet. Ze zijn nu wel begonnen met als iemand aangemeld is, om dan een terugkoppeling te doen. Die afspraak is er, maar in de praktijk zie ik ze eigenlijk niet dus dan weet ik niet of de mensen er naar toe gaan of dat we die terugkoppeling niet krijgen. Maar dat schijnt voor hen heel lastig te zijn om dat te regelen, we hebben het meerdere keren met ze erover gehad dat wij dat wel graag willen weten of mensen daar dan aankomen. En uiteindelijk, als iets is afgesloten dan horen we eigenlijk ook niets, dus dat horen we weinig terug.
***Heeft u ideeën over waar dat aan zou kunnen liggen?***Nee, eigenlijk niet. Naar mijn idee zouden ze dat gewoon wel moeten doen en ze zeggen ook wel: dat zou eigenlijk wel goed zijn en we gaan er nog eens over nadenken ofzoiets, en dan gebeurt er niet zoveel.
***Dat is wel interessant dat u dat zegt - uitleg ander interview: privacy werd genoemd -. Is dat iets wat u herkent?***Ja, daar hebben ze het in het begin heel erg over gehad, maar goed. Mensen kunnen natuurlijk daar bezwaar tegen hebben, dat kan, maar je zou actiever moeten zijn om te zeggen: vind je het goed als we de huisarts informeren want ik denk zelf dat weinig mensen daar echt bezwaar tegen zullen hebben. En dat zeiden ze zelf ook, hoor, dat dat eigenlijk niet het probleem is dat zoveel mensen bezwaar hebben. Maar eigenlijk blijft het daar gewoon liggen.
***Belangrijke aanknopingspunten. Ik ben alvast aan het denken hoe ik het ga verwerken. Heeft u nog belangrijke aanvullingen had of vragen/opmerkingen n.a.v. de casussen of onderzoek?***Nee hoor.
***Oké, dan ga ik in ieder geval de opname stoppen.***

EINDE INTERVIEW

**Interview H7ytb**Algemeen:
***Naam?***
<Naam huisarts>.
***Hoe lang werkt u al als huisarts?***Sinds 1992, dus bijna 25 jaar.
***Hoe lang bent u al werkzaam in deze praktijk?***Sinds 1999 ben ik hier begonnen als HitHA, in dienst van mijn voormalig collega. Die is 6-7 jaar geleden overleden, daarna heb ik de praktijk overgenomen samen met iemand. We zijn een duopraktijk. De laatste tijd ben ik ''de baas'' hier, ook opleider (onder andere).
***Hoe vaak ziet u op uw spreekuur gezinnen waarbinnen problematiek speelt van psychosociale aard?***Ik doe 3 dagen patiëntenzorg in de week en 1.5 dag administratie. In die drie dagen zie ik het het meeste van de praktijk omdat ik ook bekend sta als de dokter waar je naar toe moet als je problemen hebt. Mijn collega is meer van het snijden en het hakken en breken, en ik ben meer van de communicatie - dat is een hele goede samenwerking. Je krijgt dan dus wel dat patiënten denken van: ''ik heb een lastig kind, ik ga naar <naam huisarts>'' en anders gaan ze naar een andere. Ik zie 7 à 8 keer peer maand gezinnen of een kind met een probleem in een gezin (ongeveer 2 keer per week). Patiënten weten dat ze bij mij moeten zijn omdat ik hier al heel lang zit.
***Hebben jullie een POH-GGZ?***
Nee, daarom doe ik sowieso veel psychische dingen. Ik doe ook andere dingen, maar dat is wel een beetje mijn speerpuntje. Daarom heb ik ook ''ja'' gezegd tegen dat onderzoek omdat ik het interessant vind.

Casus 1 (Dave):
1. ***Wat zijn uw eerste gedachten als u zo'n patiënt op uw spreekuur krijgt?***Één van de eerste dingen die ik zou vragen is: Hoe is het thuis? Heeft hij het dan ook? Ervaar je dat daar ook zo, als moeder zijnde? Het kan namelijk heel verschillend zijn: kinderen op school kunnen stierlijk vervelend zijn en omgekeerd. Dat vind ik wel heel belangrijke informatie. Als er thuis geen enkel probleem is kun je je afvragen of je er een diagnose op moet gaan plakken. Ik probeer nader te exploreren: wat is dat precies? wat zijn het voor problemen? maakt hij dingen kapot? slaat hij anderen? Zijn er nog andere kinderen in het gezin waar ook wat mee is, of juist niet? Ik probeer een beetje een context uit te vinden. Meestal plan ik daar 20 min. voor in, dat laat ik ook aan mijn assistent weten. Ik wil even de tijd hebben, dus dan doe ik wat aan beeldvorming. Ik kijk of er al hulpverlening is geweest, of school al iets heeft gedaan (remedial teaching?). Ik krijg dan iets meer beeld, afhankelijk van hoe stringent of hoe heftig het verhaal is. Het verhaal kan namelijk heel erg wisselend. Iedereen denkt tegenwoordig meteen aan ADHD of autisme - dat is heel erg hip. Ik doe dus: of meteen actie, of ik zeg ''ik wil je nog een keer terugzien, zonder het kind of in een andere situatie. Of juist met kind, want vaak komen ouders eerst zonder kind om even rustig te kunnen praten. Dat doen mensen vaak uit zichzelf en dan wil ik zo'n kind ook zien, niet omdat ik dan heel veel met zo'n kind ga praten maar ik wil wel kijken hoe hij het doet (gedragsobservatie). Ik kijk hoe een kind zich gedraagt in de spreekkamer, maar ook in de wachtkamer als hij weer weggaat, dat kan ook nog wel eens verschillen. Dus in eerste instantie: inventarisatie, maar als ik onmiddelijk al denk dat het kind voor geen meter spoort dan zal ik al snel een vervolgactie doen.
***Ik hoorde u de opinie van het kind en van de ouders benoemen, zijn er nog andere personen waarvan u zegt: ''daar wil ik de mening ook van horen?''***Het liefst wel van school, maar er zijn hier een aantal scholen in het dorp. Mijn ervaring is dat dat niet handig werkt, dat is niet omdat iemand het niet wil maar gewoonweg omdat het niet gebeurt. Als ik school bel, dan zeggen ze niks omdat het een privacyding is (misschien hebben ze daar nog gelijk in ook). Als school mij belt dan zeg ik sowieso niets omdat het vertrouwelijke informatie is. Het moet wel heel extreem zijn als wij contact hebben met school. Heel soms krijgen we wel eens een signaal van de schoolarts.
***Wat in het verhaal van deze casus triggert u om school te benaderen?***
Ik ga alleen een juf bellen als ik het gevoel heb dat het totaal uit de hand loopt, dat doe ik niet meteen.

**2. *Heeft u al een beleid in het hoofd als u dit zo leest?***
Het is in ieder geval duidelijk dat het niet alleen op school is maar ook thuis, dat geeft dus iets meer beeld. Als ik het zo zie klinkt het erg ongeconcentreerd. De kinderneuroloog kan ik niet zo goed plaatsen, want dat bedplassen kan nu misschien een rol spelen maar dat zegt me niet zo veel. Het is dus blijkbaar begonnen vanaf groep 3, als er dus wat verwacht wordt van die kinderen (in groep 1 en 2 hoef je alleen nog maar met blokken te bouwen). Ik denk: als ik dit zo nu erbij lees, dan zou ik nog steeds niet weten of het ADHD of autisme is, maar ik zou wel denken dat er iets niet klopt omdat ouders ook een probleem signaleren. Als ik die actie al niet in de vorige consult had genomen, had ik het nu wel gedaan. Wat wij hier in <naam plaats> hebben is dat jeugdzorg in principe geregeld is via de gemeente, dus daar zou ik wel contact mee opnemen (Centrum voor Jeugd en Gezin). Dat is uiteindelijk opgedragen uit overheidswege dat gemeentes nu zelf hun jeugdzorg moeten vormgeven. Ik heb goede contacten met CJG via de mail en alles. Wij hebben hier in het dorp best wel redelijke samenwerking. Toch gaat het niet altijd even makkelijk omdat ook zij heel terughoudend zijn met het geven van informatie. Ik denk dat dat het sleutelwoord is in alles, dat je wel als hulpverleners ongeremd over kinderen (zeker als er problemen is) moet kunnen overleggen: wat gaan we doen? Ik zie wel vaak dat als ik één keer tegen een gezin met problemen heb gezegd dat ze contact moeten opnemen met Jeugd en Gezin dat ze verdwijnen in dat circuit en dat ik er dan nooit meer iets van terughoor. Het maximu wat wij nu hebben afgedwongen als huisartsen is dat wij terughoren dat iemand in behandeling is genomen of dat er contact is gelegd -that's it. Als patiënten explicitiet toestemming geven dat er met elkaar mag worden overlegd, dan kan dat gebeuren. Dat gebeurt vaker van onze kant dan van de hulpverlener- (JGZ-)kant, maar als patiënten dat niet doen dan zal nooit iemand ons iets vragen. Ik vind dat heel ongemakkelijk.
***Als ik het goed begrijp noem je hier een verbeterpunt?***Ja. De wet zegt: als het in het belang is van de patiënt (het kind) dat hulpverleners met elkaar overleggen dan is het absolute onzin om toestemming te krijgen van een patiënt. Het leeft echter, zeker in de psychologie dat dat niet mag. Misschien komt dat omdat iedereen bang is om een stempel opgedrukt te krijgen (de stempel schizofrenie roept meer negatieve gevoelens op dan bijv. een appendicitis). De zorg voor een probleemgezin kan daardoor opeens een beetje buiten ons gezichtsveld komen omdat ze verdwijnen in dat circuit en wij niet meer weten wat er gebeurt. Ter vergelijking: bij iedere oorontsteking worden wij wel geïnformeerd. Het is heel jammer dat er vanuit de psychologie/psychiatrie een beeld bestaat dat er niet overlegd mag worden als mensen aangeven dat ze dat niet willen. Ik denk zelfs dat je het überhaupt niet moet vragen, maar dat is mijn mening. Die mag ik zeggen, toch?
***Uiteraard, daar zit ik hier voor!
Je noemt een interessant punt: je hebt het erover dat je het CJG graag zou willen laten meekijken, moet ik dan ook denken aan diagnostiek?***We hebben in de regio een aantal instanties, voorbeelden zijn <namen specialistische GGZ-instellingen> en ook is er het <naam specialistische GGZ-instelling>. Dat is een organisatie waar wij van oudsher mee werken. Alleen is het nu in principe de bedoeling dat het Centrum voor Jeugd en Gezin de port 'd entree wordt voor verdere verwijzing. Er zit iemand van <naam specialistische GGZ-instelling> in het CJG, vanuit het GGZ, maatschappelijk werk e.d. en die zouden de zorg moeten kanaliseren, alleen ik kan niet weten of dat ooit gebeurt omdat de terugkoppeling niet plaatsvindt. Ik vind het verbijsterend dat de terugkoppeling zo slecht gaat.
***Maar het contact dat je wel hebt, gaat dat dan via telefoon/per mail...?***Ik geef de patiënten een verwijzing mee met het verhaal van wat ik in de spreekkamer heb gehoord. Ik zeg dan dat ze een bepaald nummer moeten bellen. Laatst heb ik afgesproken met iemand van het CJG dat ik wil weten dat iemand is gekomen. We krijgen nu dus wel een terugkoppeling dat iemand contact heeft genomen?
***En als iemand dat niet doet?***Dan weet ik dat dus niet, dan moet maar blijken of ze weer eens bij mij terugkomen. Als ze terugkomen zie ik in het dossier staan dat ze de vorige keer ook al zijn geweest (of voor een ander kind), dan vraag ik of ouders/verzorgers er al wat aan hebben gedaan. Doorverwijzen zegt weinig over het feit of iemand al dan niet er heen gaat (dat is ook zo als je naar het ziekenhuis doorverwijst).
***Spreek je de patiënt er dan op aan?***Ik vraag het, je kunt natuurlijk niet er iemand met opgeheven vingertje op aanspreken. Ik vind het vervelend als patiënten laat zeggen dat ze eigenlijk niet doorverwezen hadden willen worden. Dan denk ik ''zeg dat dan eerder''.
***Wat zijn veelvoorkomende redenen om niet te gaan?***Dat weet ik niet precies omdat ik het niet altijd hoor, maar ik denk dat veel mensen het genant vinden om een kind te hebben met een probleem. Sommige mensen denken dat het een tijdelijk iets is (bijv. de overschakeling van groep 2 naar 3), of dat het probleem zichzelf oplost. Een andere verklaring is dat ze opnieuw zwanger zijn en het probleem van het andere kind vergeten, dat gebeurt natuurlijk ook. Ik denk dat de soep niet altijd zo heet wordt gegeten als dat deze wordt opgediend. Wat ik al zei: ADHD wordt al snel gediagnosticeerd, bij wijze van spreken als je als kind één keer een grote mond hebt dan krijg je dit etiket al. Het moet tegenwoordig allemaal perfect zijn. Dat zeg ik ook wel eens tegen ouders: stel zometeen heeft hij ADHD- en dan? wat ga je dan doen? Voeder je hem dan Ritalin (dat zeg ik natuurlijk heel anders) of accepteer je hoe hij is?
***Dus eigenlijk vraag je dan de hulpvraag uit?***Ja tuurlijk: wil je het weten of zijn jullie dan bereid om B te zeggen als je A hebt gezegd. Als je de diagnose krijgt, wat ga je doen?

3. ***Is hier informatie gegeven die je beleid doet veranderen?***Nee, soms als ik niet precies weet wat ze daar nou aan het doen zijn dan kan ik er niet zoveel mee. Ik zou die ouders toch willen aanbevelen om nadere diagnostiek te gaan doen en misschien dan zelf te verwijzen naar <namen specialistische GGZ-instellingen>. Soms bel ik, heeft niet altijd veel zin maar ik probeer het. Ik denk dat dat het belangrijkste is.
Casus 2 (Sanne):
1. 2. en 3. tezamen.
***Zou je kunnen benoemen in welk opzicht deze casus nou verschilt van de vorige? Wat zijn de belangrijkste dingen die opvallen?***In ieder geval dat het een laagbegaafd iemand is uit een groot gezin. 5 kinderen, dat vind ik nogal wat in deze tijd. Die moeder is ziek, zwak en misselijk. Ik denk dat Sanne een ouderrol krijgt omdat ze ook voor die kinderen moet gaan zorgen, terwijl anderen er last van schijnen te hebben. Dat vind ik dan wel apart. Dat kind zit in een hele lastige situatie, als oudste zit zij in een soort ouderrol. Die vader zal denken: ''help ik heb vijf kinderen, ik ga gauw naar m'n werk - wat ik heel erg goed begrijp (haha)''. School dreigt de huisarts te bellen - alsof ik een soort boeman ben. ***Wat is je plan van aanpak?***Ik weet niet zo goed wat ouders willen.
***Dus je zou de hulpvraag verder uitdiepen?***Ja, wie heeft er nou een probleem? Sanne wil misschien niet graag naar school. Ik vind het wel een hele lastige casus. In ieder geval is het zo dat zij weinig naar school gaat/thuis wordt gehouden om moeder te onlasten. Dat is een rol waar zij helemaal niet op uitgerust is, want dat kind is 14. Hier moet wel hulpverlener op. Ik weet niet of ik persé diagnostiek moet, want ze is al bekend met lage intelligentie. Ze zal wel ergens bekend zijn, dat kan haast niet anders. Als ik dit zo lees, zou ik denken dat dat hele gezin ergens in de hulpverlening bekend zou moeten zijn. Ze hebben nog een kind dat zwak begaafd is, moeder die het niet meer aan kan etc.
***Hoe zou je prioritering zijn? Je stelt voor om het gezin als geheel zorg te bieden. Zou je Sanne eerder zorg aanbieden dan moeder of...?***Nee ik denk dat het een systeemding moet zijn. Ik denk ook dat die vader bij z'n haren getrokken moet worden. Ze hebben ook geld tekort, die vader werkt dag en nacht. Ze hebben ook financiële zorgen. Het gezin wordt een beetje aan z'n lot overgelaten. Ik denk dat dit typisch iets is voor een systeemaanpak. Als ik dit op mijn spreekuur zou zien dan zou ik geneigd te zijn om met veel mensen om de tafel te gaan zitten. Ik nodig ze dan uit, ik ga dat trouwens over 2 weken ook doen, en dan wil iemand hebben van school, de huisarts, iemand van JGT of wat dan ook en ouders en misschien kind om te inventariseren wat er is en waar het misgaat en wat gaan we doen? Als het kind niet naar school gaat, dan moet er hulp in huis komen, een SPV-er o.i.d. Ik denk dat die andere kinderen ook niet veilig zijn (er zitten er nog immers 4).
***Zou je die dan allemaal ook aan tafel willen hebben?***
Nee, die zijn nog te jong. Ik weet ook niet of ik die Sanne erbij wil hebben. Ik denk dat ouders het probleem hebben, ik denk dat Sanne er slachtoffer van is. In eerste instantie moeten die mensen er achter staan dat er een interventie komt. Er wordt nu een 1.5 jarige gedeeltelijk opgevoed door een beneden gemiddeld slim iemand, dat kan nooit goed gaan - denk ik.
***Stel, ouders komen hier met iemand van het JGT aan tafel. Ouders zeggen bijvoorbeeld: wat ons betreft is er geen probleem (althans niet bij onze opvoeding). Stel ze zijn het niet met je aanpak eens, wat dan?***Dan zou ik een melding doen bij <naam advies- en meldpunt voor huiselijk geweld en kindermishandeling>. Als ik me serieus zorgen maak, dan doe ik dat en dan doe ik dat ook niet anoniem.
***We hebben in deze casus natuurlijk een beetje vals gespeeld doordat ik alle informatie meteen heb gegeven, maar waar in het verhaal zou je zeggen: ''tot zo ver zou ik gaan met behandelen en vanaf dit punt zou ik doorverwijzen?''***In deze casus zou ik dat doen na informatie 2. Het is toch een beetje een gedragding wat anders is, moeder die niet goed functioneert. Een kind dat vaak niet naar school gaat, dan weet je al dat daar wat zit.

Casus 3 (Melany):
1.2. en 3. tezamen
***Wat is je eerste indruk van deze casus?***
Ik denk dat Melany aan het puberen is en dat die ouders niet op één lijn zitten, wat maakt dat zij ouders goed uit elkaar kan spelen. Ze valt een beetje tussen wal en schip. Ik vind het wel erg goed dat ouders wel samen komen. Het ligt aan vader en niet aan Melany, omdat Melany niet wordt begrensd. Dat zou mijn eerste ding zijn, daarna zou ik de focus leggen op Melany. Ik denk dat ze een laag zelfbeeld heeft.
***Wat zou je plan zijn?***Dit zou een casus kunnen zijn waar ik mezelf op stort (in z'n geheel), omdat je niet elke puber waar stress thuis is moet doorverwijzen. Je moet eerst inventariseren en duidelijk krijgen van hoe het bij de een is en hoe het bij de ander is. Het is niet alleen bij vader thuis, maar ook bij moeder dus die kunnen blijkbaar ook wel botsen. Ik zou van ouders willen horen hoe die mensen er in staan, blijkbaar zit er nog wel wat wrijving, daar valt nog wel winst te behalen. En dan zou ik ouders er heel erg op wijzen dat zij de opvoeder zijn. Het is misschien leuk dat je 15 bent en dronken bent, maar dat is gemiddeld genomen niet de bedoeling voor iemand van 15. Vinden ouders dat goed? Hoe gaan ze daarmee om? Hoe houden ze dit gedrag in de hand of laten ze dit kind gewoon vliegen? Ik denk dat ze zo druk met hun eigen gedoetje zijn dat zij te weinig begrensing krijgt. Ze moet structuur aangeboden krijgen. Als je thuis de rust niet kunt vinden dan moet je op huiswerkbegeleiding. Ik zou ook graag met Melany willen spreken alleen.
***Dat vraag ik me dan inderdaad af, wil je ze afzonderlijk spreken?***
Ik zou ze eerst afzonderlijk willen spreken. Dat meisje is nog niet alleen gekomen. Voor hetzelfde geld zegt ze ''ik haat het bij m'n vader want dat ''stomme'' wijf zit er altijd''. Je moet nog maar zien of je zo'n kind bereid krijgt om te praten want dat doen ze niet standaard. Aan de andere kant zou ik bij deze casus zelf willen kijken hoe ver ik kom. Dat vind ik altijd wel leuk om te doen.
***Een vraag die grenst aan de vraag die ik bij de vorige casus stelde. Stel Melany is het niet met je beleid eens, ze vindt dat er geen probleem is, wat zou je dan doen?***
Ik zou in ieder geval met die ouders praten om ze heel erg duidelijk te maken dat zij de verantwoordelijkheid hebben over dat kind. Er zijn allerlei manieren in de opvoeding waarmee je kunt zorgen dat je een kind een beetje op het rechte pad probeert te houden. Je weet niet hoe ze zijn, voor hetzelfde geld zijn ze reuze christelijk en mag ze überhaupt nooit wat. Daar moet je wel over praten en je kunt ze altijd de tip geven om te gaan praten met een opvoedtherapeut: wat moet ik doen om een puber in de hand te houden? Dit klinkt veel minder dan psychiatrie dan bij de vorige casus voor mij.
***Dat is dan ook de reden om de behandeling zoveel mogelijk in eigen hand te houden in deze casus?***Ja. Je kunt ook niet alles en iedereen verwijzen. Ik ben oud en ervaren en heb een vorm van overwicht (haha). Meestal lukt het me om een heel eind te komen. Als het me niet lukt zeg ik dat ze hulp moeten gaan zoeken

Algemeen:
***Van de drie casus die ik zo voor heb gelegd, zitten er nou casus tussen die u veel ziet?***Casus 3 sowieso, casus 1 (je ziet veel kinderen waarbij gedacht wordt aan ADHD of een autisme-spectrumstoornis) en casus 2 zie ik minder. In mijn praktijk kan ik zo de mensen opnoemen waarbij een ''niet OK''-situatie speelt en dat zijn misschien 3 gezinnen van 3800 à 3900 patiënten die ik heb. Dit zie ik wat minder, omdat dit <naam plaats> is. Mensen hebben hier geen financiële problemen, dan moeten ze het wel heel bont gemaakt hebben. Die andere 2 zie ik wel veel.

EINDE INTERVIEW

**Interview H6icv**Algemeen:
***Hoe lang bent u al huisarts?***Ik ben huisarts sinds 2006, dus dan ben ik aan m'n 12e jaar begonnen. 11 jaar als huisarts dus, werk ik.
***Hoe lang al in deze praktijk?***Even lang, het is de praktijk van mijn vader. We hebben het eerst samen gedaan en daarna heb ik het overgenomen. In 2006, toen ik klaar was als huisarts ben ik hier dan 2 dagen in de week, 3 dagen in de week, toen overgenomen. Dus hij afbouwen en ik opbouwen.
***Het is echt een soort familiebedrijf?***Ja, bijna wel. Alleen we hebben dus niet heel lang samengewerkt omdat hij inmiddels vanaf 2010 met pensioen is, dus ik doe het nu alweer het 7e jaar alleen. Weliswaar niet alleen alleen maar als praktijkeigenaar en toen is hij gestopt.
***Hebben jullie veel patiënten hier?***2500, 2600 - het fluctueert een beetje, soms wat meer soms wat minder maar dus net iets boven de norm.
***En voor mijn begrip: hebben jullie alleen patiënten hier in <naam plaats> of ook nog daarbuiten?***Een enkeling die dan van <naam plaats> die dan naar de buurgemeente <naam gemeente> (net op de grens) die blijven dan nog wel 's hangen, maar ik probeer het wel echt zoveel mogelijk binnen de gemeente te houden. Natuurlijk heb je altijd mensen die zeggen: '' ik werk hier '' of ik woon wel ergens ver weg maar ik ben toch altijd wel hier. Ja, weet je: mensen weg sturen kan je niet, je kunt alleen het benaderen. Dus ja, eigenlijk grotendeels <naam plaats> - een enkeling daarbuiten maar het zijn er maar een paar.
***En alvast toespitstend op het onderwerp waar we het zometeen over gaan hebben: hoe vaak komt het nou voor dat u gezinnen, kinderen, jongeren met psychosociale problematiek op het spreekuur tegenkomt?***Ik vind jongeren of gezinnen wel echt een verschil. Jongeren met psychosociale problematiek natuurlijk vele meer dan echt gezinnen waarbinnen een probleem speelt. Ik denk gezinnen 5 à 6 per jaar, dat er in een gezin echt iets speelt. Als je zegt jongeren met psychosociale problematiek zou ik bijna willen zeggen: 2-3 per maand of zo. Echt wel meer.
***En wat voor problematiek is dat dan, bijvoorbeeld?***Want jongeren dan heb je het echt over tot 18 jaar, neem ik aan hè?
***Ja***.
Nou ja; laag zelfbeeld, onzekerheid, wat wil ik met mijn leven, beroepskeuzeproblematiek, ook soms het verhaal van: '' ik heb het idee dat ik toch ADHD heb' of ik voel me toch niet helemaal thuis in de groep waarmee ik omga, identiteitsproblematiek - dus niet allemaal gelukkig te zware dingen. Maar dat zie je het meest.
***En komen die jongeren daar dan zelf mee?***Dat is heel wisselend, is mijn ervaring, heel wisselend. Meeste komen met moeder. Nooit vader, altijd moeder. Ik weet niet of dat iets is van <naam plaats>, maar dat is wel opvallend. 1/3e komt alleen, 2/3e komt met moeder en soms vader.

Casus 1 (Dave):
1. ***Wat is de eerste indruk die je krijgt van dit probleem?***Wat dan bij mij altijd gelijk opvalt dat moeder met Dave komt en ''school heeft gezegd'', dus waar ik altijd mee begin is te vragen wat Dave zelf vindt van het verhaal. Of hij ergens last van heeft of dat hij het gevoel heeft dat het op school niet helemaal goed gaat. Nu is Dave pas 6 jaar, dus dat is vrij klein. En dan zou ik ook aan moeder vragen of zij dingen ziet die ze misschien bij andere kinderen - misschien heeft ze meer kinderen, dat weet ik in dit geval niet - maar of zij dingen ziet bij Dave die anders zijn dan bij andere kinderen - van haar of bij neefjes of nichtjes. Dus echt eerst even heel oriënterend van: ''het is gelijk zo ADHD, dat zegt school'' maar ik heb altijd zoiets van: terug naar de basis en informatief van wat zijn er dan voor problemen of waar lopen ze dan tegen aan?
***En eventueel ook de vraagstelling vanuit moeder?***Ja en: school kan dat wel zeggen en vinden maar hoe staan ouders en moeder en Dave zelf er tegenover om daar inderdaad iets mee te doen? Willen ze dat überhaupt of is het echt een wens van school?
***En als ik het goed begrijp, je noemt eerst de opinie van Dave***
... ja...
***En daarna de opinie van moeder. Is dat ook misschien een soort hiërarchie die je qua informatie in je hoofd aanbrengt?*** ***Dat je de opinie van Dave bijvoorbeeld belangrijker vindt dan die van moeder?***Nee, niet een prioriering. Ik vind dat alle informatie daarin welkom is. Nee, alleen vind ik het altijd wel verhelderend om te zien hoe een kind het zelf ervaart - of een jong volwassene - of hoe de omgeving er tegen aan kijkt. Meer dan dat wil ik er niet mee beogen, ofzo.
***Oké, dan gaan we een consultje verder.***
Moeder komt nu met vader, hier is de vader (haha).
***Haha, redelijk bijzonder misschien.***Ja, haha.

2. ***Er wordt nu wat meer informatie vrijgegeven: welk beeld krijg je nu van het probleem?***Wat het is zou ik nog geen oordeel over willen geven, maar ik pik wel een aantal dingen op uit het verhaal die ik wel afwijkend van de norm vind. En natuurlijk kan die norm wat variëren, maar er zijn toch wel een aantal kenmerken die geschetst worden van de aanvallen van wegtrekken, het bedplassen. Alles bij bij mekaar schetsen die dat er toch wel meer met Dave aan de hand is dan bij een gemiddeld kind van 6 jaar. En er speelt wat aan de kant van de familie van moeder. Dus er komen familair toch wel wat dingen voor op gedragsmatig gebied, dus dat triggert je wel om toch te kijken van: zou er wat meer aan de hand kunnen zijn? Ik vind dan wel heel bijzonder dat 'ie wel bij de kinderneuroloog loopt, want ik neem dat ik dat normaal gesproken geweten had dus dan is de introductie anders maar dat is zeker iets wat daarmee besproken zou kunnen worden. Dus dan zou ik me kunnen voorstellen dat ik de neuroloog 's opbel of dat ik ouders vraag om met de neuroloog deze problematiek te bespreken.
***Dus als ik het goed begrijp, je zou eerst met name het somatische willen verhelderen?***Ja, want ik denk niet dat je het gedragsmatige en het somatische los van elkaar zou kunnen zien als iemand bij de kinderneuroloog loopt, denk ik. Kan natuurlijk wel, maar alle partijen moeten wel op de hoogte zijn dat dit speelt.
***Zijn er nog andere acties die je op dit moment zou willen doen?***Nou ja, ouders geven aan dat ze graag zouden willen uitzoeken wat er aan de hand is, dus ik zou wel willen vragen wat ze daarmee willen: willen ze inderdaad gewoon hem onderzocht hebben op een diagnose of willen ze vooral hulp bij de begeleiding. Dus dat je wel de hulpvraag van ouders duidelijker maakt. Sommige willen alleen een etiketje, anderen willen echt hulp van hoe ze met z'n gedrag moeten omgaan. En dan zou ik hem daarvoor wel naar een instantie verwijzen die dat dan kan gaan uitzoeken. Ik kan dat zelf niet, ik ben zelf niet een huisarts die ADHD vaststelt en dat begeleidt of instelt. Maar ik zou dat echt niet doen zonder eerst contact gehad te hebben met de kinderneuroloog.
***En stel: uiteindelijk blijkt dat somatische aspect gering, onvoldoende verklarend voor het gedrag van Dave; aan welke instantie zat je dan te denken?***Gezien toch het vrij specifieke verhaal van Dave, maar toch wel zijn jonge leeftijd. Zou ik eerst - hier in <naam plaats> hebben we een kinderpsycholoog - eerst toch 's willen vragen of zij oriënterend kan kijken in welke hoek zij denkt dat het zit. Want school denkt aan ADHD, als ik het verhaal lees kan het ook heel goed, maar bij jonge kinderen vrees je toch dat er andere dingen een rol kunnen spelen. Dus ik zou eigenlijk eerst beginnen met ofwel <branchevereniging voor organisaties die jeugdhulp, jeugdbescherming en/of jeugdreclassering bieden> of inderdaad de lokale kinderpsycholoog. Dat is per gemeente natuurlijk anders, dat kan allebei. Er zitten bij ons ook allebei dezelfde mensen in, dus dat maakt het wat ingewikkeld. In ieder geval vragen of zij oriënterend en ook een keer op school kunnen gaan kijken wat zij zien, wat zij signaleren - ook misschien wat testen kunnen afnemen van wat er is en dan eventueel (stel de aanwijzingen zijn er dat het hier ADHD betreft) ADHD-gericht verwijzen naar, we hebben hier in <naam stad>, <naam specialistische GGZ-instelling> of <naam specialistische GGZ-instelling>.
***Duidelijk stappenplan, in ieder geval. Dan stel ik voor om naar het derde consult te gaan.***

3. ***Ja, dan komt opeens het JGT om de hoek kijken. Die hadden we nog niet gehad. Is dat iets wat in je gedachtengang nog de revue heeft gepasseerd, het JGT?***Nou, dat is wel wat ik zeg, inderdaad: van altijd in overleg met wat willen ouders of wat wil het JGT. Er zit ook vaak een kostenplaatje aan of willen mensen - ik ervaar toch een hoge drempel van ouders om naar het JGT-team te stappen omdat ze toch het gevoel hebben (dat is misschien ook per gemeente) maar toch: ''zo groot is het probleem nou ook weer niet, dat ik het daar neer moet gaan leggen''. Terwijl als je echt met een psycholoog gericht persoonlijke aandacht, zeg maar, dan voelen ze daar veel meer voor. Ik bespreek het altijd wel, ook omdat ik altijd aangeef van ''die kunnen ook op school bemiddelen en kunnen ook op school extra hulp inzetten''. Hier in <naam gemeente> is het dan, maar dan zit ik ook in een luxe positie ook andersom, dat de psycholoog dat als die ziet dat het op school niet goed gaat dat die dan ook automatisch al met JGT-team en school contact opnemen. Dus er zit al een loop in de gemeente hier, dus dat maakt het voor ons wat makkelijk dan.
***Nog even tussendoor, voor mijn begrip, hebben jullie hier een POH-GGZ?***Nee, wij hebben geen POH-GGZ.
***Zoals je al vertelt: de jeugd/kinderpsycholoog zou dus dan contact kunnen leggen met...OK. Verder noemde je ook net iets interessants, namelijk dat ouders soms denken van: de problematiek is nog niet van die mate dat we naar het JGT moeten. Is dat ook iets wat je herkent: dat patiënten het JGT als een stapje hoger dan vrijgevestigde psychologen zien?***Nee, juist niet. Ze zien het toch als het verlengde consultatiebureautje waar je ook nooit goede informatie krijgt. Ja, dat vind ik lullig om te zeggen want zo denk ik helemaal niet over. Maar ja, dan is de vraag: wat kunnen die dan doen? Ja, ik zou niet weten waarom die daar dan naar moeten gaan kijken. Ik wil eerst weten wat er is. Dan hechten ze toch meer waarde aan de psycholoog, terwijl die ook in het JGT-team zit. En het gevoel de vuile was buiten te moeten hangen. Psycholoog klinkt wat beknopter dan een team.
***Ik snap het. Hoe zijn je eigen ervaringen met het JGT als je daar naar doorverwijst?***
Op zich wel heel positief, altijd wel betrokken mensen maar wel lastig ook omdat als mensen zichzelf aanmelden niet altijd een terugkoppeling naar de huisarts mag omdat mensen daar geen toestemming voor geven. Dat ervaar ik wel echt als een beperking, dan lopen gezinnen bij het JGT-team en dan weet ik dat als huisarts niet. Dat vind ik lastig, maar verder de keren dat ik ouders wel verwezen heb en uitgelegd heb wat ze kunnen bieden, ja positief. Ook altijd netjes afsluitende brief gekregen of wat er aan het gebeuren er is met ook altijd een contactpersoon erbij dus dat werkt wel goed.
***Oké, dus de terugkoppeling loopt wel goed?***Ja, als de toestemming er is, of als het via mij gegaan is gaat het altijd goed. Als ik verwijs mogen ze automatisch wel terugkoppeling of als ouders en kind toestemming geven. En als mensen zichzelf aanmelden buiten mij om, ja dan weet ik dat niet.
***Ja, dat is inderdaad wel een interessant punt. Ik ga natuurlijk verschillende gemeenten langs, in elke gemeente is dat weer anders geregeld. Maar inderdaad dat stukje terugkoppeling, daar hoor ik veel verschil in - inderdaad. Wat ik dan hoor is dat dat bijvoorbeeld zou liggen aan privacy. Dat dat toch een vraagstuk is bij JGT.***
Ja, nou het is gewoon echt informed consent van delen van informatie en als daar geen toestemming voor is..
***Is dat ook vanuit de gemeente ooit een keer bespreken?***Jawel, want toen dat allemaal is opgezet zijn er wel van die formele bijeenkomsten geweest van: hoe willen we dit allemaal gaan opzetten? en wie moet met wie contact hebben en wie moeten daarin? Ik ben daar ook wel een aantal keren bij geweest, van die lunchbijeenkomsten, maar goed dat is altijd wel een discussiepunt geweest waarbij de ene partij zegt van: ''ja dat is wettelijk privacy, dat mogen we niet geven.'' Terwijl wij zeggen van: hallo, wij hebben elke dag met die patiënten te maken en het lijkt me niet meer dan logisch dat als school het weet, dat wij als huisartsen ook weten dat het een probleem is. Maar, dat strookt nog wel 's. Voor zover ik weet is dat dat wel berustend op de Privacywet, maar of dat klopt dat weet ik nu niet want ik heb het nooit uitgezocht. Maar zo is het nu wel. Mensen moeten actief toestemming geven in <naam gemeente> als ze zichzelf hebben aangemeld.
***Voor zover ik weet is dat ook de regel.
Oké, je noemde zojuist dat je ook de beslissing zou maken om naar een vrijgevestigde kinderpsycholoog te verwijzen. Wat zijn nou elementen in die samenwerking die maken dat je dat doet, zeg maar? Dat die samenwerking zo goed loopt?***Nou, altijd telefonische terugkoppeling of als ik vragen heb dat je elkaar laagdrempelig mag bellen voor informatie: ''is dit iemand die je zou kunnen zien of kun je er iets mee?'' Maar ook andersom altijd netjes een afsluitende brief en uitvoerig welke onderzoeken zijn gedaan met ook uitvoerig een advies van: ik zou hem of haar verwijzen naar die en die instantie; het lijkt toch echt een probleem passend bij beginnend of ook soms wel: ''het kind is te jong, over een jaar doen we alle testen opnieuw.'' Dus goede controle erop, een goede feedback.
***Want - het kan nog wel 's per huisarts verschillen: de ene huisarts zegt: de jeugdzorg dat delegeer ik liever en sommige zeggen dat zij liever de spin in het web zijn -, hoe zie jij dat?***Nou, meer het laatste. Ik ben wel iemand die alles binnen het gezin graag volgt omdat ik zelf van mening ben dat het heel erg belangrijk is want je kunt niet elk individu los zien. Je bent allemaal onderdeel van een geheel. Ja, ik vind dat juist belangrijk dat ik daar dan van op de hoogte ben en dat ook individueel elk lid van het gezin bij mij terecht kan over dat probleem, want moeder kan het anders ervaren dan vader en Dave kan misschien zelf ook vragen hebben die hij misschien niet aan zijn vader of moeder kan vertellen - nu is Dave nog wat jong, maar als het kind iets ouder zou zijn. Dus dat is in ieder geval allemaal voor zich ..., dat in ieder geval bekend is dat ze met vragen terecht kunnen en dat is bij een psycholoog dan weer niet zo makkelijk want dat is echt voor één persoon.
***Want komt het dan ook voor dat als ze naar een kinderpsycholoog zijn geweest dat ze dan ook nog 's terugkomen, hier?***Dat vraag ik altijd, dat is ook altijd mijn insteek. Ik wel graag na 4 tot 6 weken horen hoe de gesprekken gaan, of er een klik is en of het naar wens verloopt, ook om de controle er op te houden dat ze gegaan zijn en of het biedt wat ze ervan verwacht want anders wordt het probleem nog niet aangepakt. En over het algemeen doen de mensen dat ook wel netjes.
***Duidelijk verhaal, in ieder geval. Dan gaan we alweer naar casus 2.***

Casus 2 (Sanne):
1. 2. en 3.
Is Sanne nou een jongetje of een meisje, Sanne helpt veel... oh, wacht andere zusje?
***Het is een meisje (haha).***Ja (haha), ik wou al zeggen.
***Heel ander probleem, natuurlijk.***
Ja, maar veel meer omvattender dan alleen probleem bij één enkel persoon, het is wel echt als ik dit lees een gezinsprobleem. Hier zou wel gelijk Jeugd en Gezin in mijn hoofd opkomen.
***Vanwege het systeemaspect?***Ja, een heel duidelijk systeemaspect hierbij, waaribj ook het verhaal van Sanne met een benedengemiddele intelligentie (ik ga ook er vanuit dat dat officieel onderzocht is) ook wel ervoor zorgt dat er heel veel instanties zijn die daardoor ook wel wat extra ondersteuning kunnen bieden richting school en richting Sanne (bijv. <naam van een coöperatieve vereniging van 20 regionale organisaties, die zich inzetten voor mensen met een beperking en hun netwerk>). En die zitten ook allemaal in het CJG-team. Vandaar dat ik denk van: nou daar zou dus wel echt als gezin een hoop te halen vallen.
***Dus als ik het goed begrijp: dus ook zorg voor ouders, niet alleen voor Sanne en zusjes?***Ja. Maatschappelijk werk gezien de financiën misschien, voor zover natuurlijk ouders daarvoor open staan. Maar ik denk wel als ingang Sanne zijn dat wel dingen waarin hulp te bieden is.
***En als we nu kijken naar casus 1 en casus 2: zijn dat nou casussen die bekend in de oren klinken?***Casus 1 heel duidelijk, casus 2 in veel mindere mate. Of je hoort het niet, dat weet ik natuurlijk niet. Dat is een goede vraag en ik denk dat ik heel veel patiënten ook wel goed ken maar je kan natuurlijk missen dat mensen toch hier niet mee komen. Dus ja, dit komt minder vaak voor dat dit echt als hulpvraag op je bordje komt.
***En als ik het goed begrijp: het grote verschil tussen deze casus t.o.v. casus 1 is dus dat systeemaspect?***Ja.
***Dus eigenlijk meteen door naar JGT?***Nou ja, ik zou dat wel voorstellen, ja, en anders zou ik in ieder geval aanbieden aan ouders om een keer met elkaar hier te komen praten en met elkaar 's te inventariseren wat voor vader nou belangrijk is, wat voor moeder en wat voor Sanne. En meer oriënterend, niet zozeer dat je daar zelf iets mee zou kunnen maar wel om de situatie nog wat duidelijker te krijgen hoe 'ie er thuis aan toe gaat.
***En wat betreft zusjes en broertje?***Idem dito, natuurlijk: vragen hoe het met hen gaat, hoe de situatie met hen is op school maar er ook wel op hopend dat een CJG-team (ik ga er even vanuit dat ik ze zover krijg om daar naar toe te gaan) dat oppikt. Het zijn toch wel heel wat zorgen als je dit zo leest, in dit gezin.
***Dan nog een andere vraag: ouders zitten niet altijd op één lijn qua opvoeding, wat zou je nou doen als ouders compleet verschillen in wat zij denken dat het beste is? Hoe zou je daar op inspelen? Bijv. vader wil helemaal niet naar het JGT.***
Dus in het geval als ze helemaal niet zouden willen?
***Ja.***
Nou, wel weer inderdaad beide uitnodigen en 's horen van waar dan de grote verschillen in zitten. Ik zou proberen bij beide partijen te verduidelijken hoe eenieder er dan over denkt. Zoveel mogelijk objectief blijven, alhoewel ik dat wel lastig zou vinden omdat je wel snel geneigd bent om vanuit je eigen kader te zeggen:''dat is goed, dat is niet goed.'' Ja en dan misschien wel te zeggen: misschien een keer een systeemgesprek met een psycholoog voor de ouders aan te bieden, als ze niet naar een CJG zouden willen.
***En dan weer via dezelfde vrijgevestigde psycholoog?***Ja.
***Een aantal interessante punten heb je aangesneden. Samenvattend: het bevat een systeemprobleem waarin deze casus met name verschilt van die eerste casus en dat daarom het JGT in jouw optiek het beste is aangewezen. Dan wel nog (een vraag) over specifiek de zorg voor ouders: zijn er dingen die je zelf nog zou willen meegeven aan ouders, bijvoorbeeld in de opvoeding? - nog voordat ze naar JGT gaan.***Ik zou wel zeggen: het is voor Sanne wel belangrijk dat jullie als ouders één lijn trekken en dan in het midden latend wat dat dan moet zijn, maar dat nog wel benadrukken dat het voor kinderen belangrijk is dat ze één houvast hebben en als de één iets anders zegt dan de ander dat dat dan niet goed werkt - dus dat ze daar wel samen over na moeten denken. En afspraken daarover moeten maken.
***Ja, dus je probeert eigenlijk de verschillende meningen bij elkaar te krijgen? Oké, dan gaan we alweer door naar casus 3*.**

Casus 3 (Melany):
1. 2. en 3.
Ja.
***Is dit een herkenbare casus?***Ook nog steeds wel iets minder dan casus 1 maar wel ook herkenbaar, ja zeker. Maar ook wel minder voorkomend.
***Wat is nou een kernwoord of dat zijn meerdere kernwoorden waarvan je zegt: nou dat kenmerkt het probleem ten opzichte van die andere twee casussen?***Gescheiden ouders met een dochter die problemen heeft op school en eigenlijk geen hulp wil. Dus waar in de andere gevallen wel een gezin is en betrokkenheid is van ouders is dat misschien nu ook wel zo maar verschillend in opvatting omdat ouders zijn gescheiden - er waren al onderlinge verschillen in ideeën - en Melany lijkt daar een lekkere tussenweg in te vinden zelf.
***De vraag die ik dan net stelde is dan ook meer van toepassing op deze casus. Wat zou je doen als ouders verschillen qua opvatting?***Nou ja, wel weer benadrukken dat ondanks de scheiding het heel belangrijk is in de goede zorg van Melany te zorgen dat ze daar in ieder geval uit komen samen. En in dat opzicht ook samen voor Melany hulp te gaan zoeken. Nu is ze nog maar 15, als ze 18 is kunnen ze niks meer voor haar doen. Nu kan het nog enigzins, met medezeggenschap van Melany. Dus voordat het op school echt mis gaat.
***Dus je moet er vroeg bij zijn?***
Ja, in dit geval denk ik wel.
***En hoe zou je dit aanpakken?***Ik zou wel vragen of ze met Melany komen want de eerste twee keer zijn ouders dus samen gekomen en de eerste keer moeder alleen en gezien het feit dat Melany 15 is zou ik zeggen dat we niks kunnen of willen doen voordat Melany ook bij het gesprek is geweest en hoort wat ouders vinden en wat ik ook aan haar wil vragen wat er is en dat is dan een gesprek of met beide ouders of alleen met Melany en dan alleen met ouders - maar ik wil in ieder geval Melany ook even alleen spreken en vragen aan haar hoe het komt wat ouders signaleren en hoe zij het zelf ervaart.
***Hoe je pak je dan zoiets aan? Splits je bijvoorbeeld een consult?***
Ja, ik laat ze of beide binnenkomen en daarna stuur ik ouders weg of andersom.
***En denk je nog aan de psycholoog in dit geval?***Ja, dat zou in mijn geval heel erg afhangen van wat Melany mij zelf te vertellen heeft, want de casus is zo breed te interpreteren: het zou best zo kunnen zijn dat Melany gewoon heel ongelukkig is in de situatie met haar gescheiden ouders, het zich niet prettig voelen bij vader of bij moeder. Dus dat er met Melany nog niet zoveel aan de hand is maar dat ze gewoon echt niet goed in haar vel zit omdat ze gewoon niet steun krijgt die ze nodig heeft, dus dat het meer de ouders zijn die het probleem hebben dan Melany zelf. En dat het meer een reactie erop is. Ik zou vooral van haar willen weten van haar waar zij zich niet goed bij voelt en daar dan echt wat mee doen.
***Dus eerst zelf uitdiepen en vervolgens het vervolgplan schetsen?***Ja, en ook een beetje goodwill bij Melany kweken in de zin van dat ze ook wel open staat voor behandeling en weet dat ze hier dan terecht kan als ik haar huisarts zou zijn. Ook zonder haar ouders, als zij behoefte heeft om iets te spuien of iets te bespreken.
***Het gaat hier dan ook over flink wat alcoholgebruik, zou je nog op somatisch gebied iets doen (als daar aanleidingen voor zijn)?***Nou daar had ik niet in eerste instantie aan gedacht met dit verhaal, in alle eerlijkheid. Kijk, het kan misschien wel zijn - maar dat is meer in het laatste stukje wat ik lees: dat ze somber is en piekert - zou ik wel willen voorstellen om 's een keer een bloedbeeld te doen (schildklierfunctie en vitamines) vragen of ze goed eet, maar niet omdat ik verwacht dat daar wat uit komt.
***Ja precies, het is ook een vraag om het hele plaatje in beeld te krijgen.***Ja tuurlijk, maar vaak hoor je moeder dan ook zeggen: ''het gaat niet goed, ze ziet wit en dan denk je automatisch meer aan...'' en het is gefocust op psychische zorg dus... (haha)
***(Haha), het is een beetje gebiased, ja***Ja, precies. Maar dat zou je dan wel moeten doen.
***Nou, hartstikke duidelijk. Nu is het zo, we zijn aan het einde gekomen van de casus. <uitleg artikel en streven naar aanbevelingspunten> Heb je nog tips voor die aanbevelingspunten, met name met het oog op het JGT?***Ja, in die zin, ja daar kan je niet zoveel aan doen omdat het wetgeving is maar wat wel inderdaad: de communicatie naar de huisarts doe. Wat ik stiekem ook zou hopen, ook al mag het niet omdat ouders geen toestemming geven, dat soms wel eens een collega van het JGT de telefoon zou pakken en zegt ''ik bel nu eigenlijk niet maar ik wil toch laten weten dat dit en dit speelt in een gezin als medewerkers in de zorg naar elkaar toe, zonder dat dat vastgelegd wordt''. Maar goed, maar dat mag je niet als vaste afspraak maken, maar dat zou ik wel als meerwaarde zien, omdat je dan als huisarts ook dat als mensen met lichamelijke klachten komen dat je daar dan ook wat alerter op kan zijn of ook een soort opening kan geven zodat mensen wel met het verhaal gaan komen. Je mag natuurlijk niet dat je iets ter ore is gekomen, dat moet in vertrouwen kunnen. Dus dat is iets wat ik met andere disciplines absoluut al wel heb maar met het CJG, maar dat kan denk ook omdat er heel veel mensen werken. En wat nog meer? Terugkoppeling, als die komt, is gewoon goed - dus daar heb ik niet zoveel aan toe te voegen. Ja, af en toe een updatelijst van wie werken er en wie moet ik daarvoor hebben? Gewoon heel praktisch: wie werken er, dat je gewoon een up to date schema hebt - gewoon heel praktisch.
***Ja, want is dat iets wat je nog merkt: heel veel JGT'ers werken part-time, is dat iets wat je terugmerkt in de communicatie?***Nou, ik werk zelf ook part-time, dus dat is inherent aan het feit dat je zelf part-time wilt werken, dan kost het gewoon wat eigen tijdsinvestering extra om toch op je vrije dag dan iemand proberen te bellen. Dus dan moet je zelf gewoon full-time gaan werken, dus dat zie ik niet als extra waardoor dingen niet goed zouden kunnen lopen. Het vergt wel inderdaad wat meer bellen over en weer, maar als de wil er is dan lukt dat wel. Het zou fijn als je weet wanneer wie er is, dan scheelt je dat belletje.
***Ook daar communicatie over? Ja, ben je van mening dat het JGT voldoende bekend is bij huisartsen?***Ik vind dat altijd zo lastig, omdat je vanuit je eigen perspectief kijkt. Ik kan me gezien de huidige uitnodigingen die wij hebben gehad vanuit de gemeente toentertijd bijna niet voorstellen dat er collega's zijn die het niet weten. Maar goed, ik hoop van niet.
***Ik geloof dat mijn vragen in ieder geval allemaal gesteld zijn, ik weet niet of jij nog aanvullingen had?***Nee, ik wens je veel succes ermee. Ik hoop dat je er wat aan had. Het lijkt me wel een heel breed onderwerp om daar straks een advies over te geven of daar iets over op te schrijven. Het is heel breed opgezet.

EINDE INTERVIEW

**Interview Ha4cv**
Casus 1 (Dave):
1. ***Wat zijn je eerste gedachten?***Wat ik me altijd afvraag: Waarom komt school ermee? Loopt hij vast op school? Eerste vraag die ik nu zou stellen: in welke groep zit hij? Hij is zes, zit hij nog in de kleuterklas? Zit hij al in groep 3 en loopt hij daarom nu vast?
Wat ervaren ouders? Herkennen ze dit? Zo ja: wanneer dan? Dus ik zou uitdiepen: wat is er aan de hand en wie heeft er last van? Dat is denk ik het eerste.
***Dus je zou verschillende opinies in kaart willen brengen?***
Ja. En is er echt een probleem? Ervaart iedereen dat ook?
***Zou je al met deze informatie iets kunnen vertellen over wat het probleem is?***
Met zo weinig informatie kan het nog alle kanten op, variërend van vrij ernstige problematiek waarbij het terecht is dat er ergens aan de bel wordt getrokken tot 'hoe erg is het dat een jongetje van 6 zich niet kan concentreren?' Het ene kind is daar sneller in dan het andere. Ik zou nog niet durven zeggen dat er nu een probleem is. Het feit dat school hem stuurt zal wel betekenen dat er een probleem is, kennelijk gaat het op school niet lekker, maar ik vind het kort door de bocht op basis van wat ik lees om meteen aan autisme of ADHD te denken. Een jongetje van 6 met frustraties en concentratieproblemen, dat hoeft nog niet meteen problematisch te zijn.
***Dus om kort te gaan: om meer informatie te krijgen over deze zaak zou je graag de verschillende opinies in kaart willen brengen?***Ja.
***En hoe zou je dat willen doen? Zou je nog mensen op het consult willen uitnodigen?*** *Nee, in eerste* instantie gaat dat via moeder. Dave zelf kan ik vragen wat hij van de situatie vindt. Verder wil ik vragen naar of er een vader is, hoe de gezinssamenstelling is en of er broertjes of zusjes in het spel zijn. In eerste instantie doe ik het met degene die voor me zit. En daar kom je meestal wel een heel eind mee, zeker als moeder zegt dat er thuis problemen zijn en dat ze echt op zoek naar handvaten is - dan moeten we dat verder uit gaan zoeken.
***Zou je nog diagnostische middelen in willen zetten?***Ik ben zelf niet zo van het zelf vragenlijsten inzetten. Ik kan me wel voorstellen dat school daar al iets mee heeft gedaan dus dat zou ik wel uitvragen. En dan komt meteen ook het stukje van hoe in <naam regio> de zorgstaat ADHD in een vat te gieten (?) dan zouden we kunnen kijken of de praktijkondersteuner daarin een start kan maken, maar dan moet het wel duidelijk zijn dat er enkelvoudige problematiek is. Dus dat het vooral het concenteren is bijvoorbeeld en het druk zijn. En niet als je echt al denkt aan een vorm van autisme, dan zou ik dat overslaan.
***Is er nog een bepaalde hiërarchie die je in de verschillende opinies zou willen aanbrengen?***Het is op zich allemaal belangrijk, want als hij op school vast loopt en ook al erkennen ouders het probleem niet dan erkennen ouders vaak ook wel dat er iets moet gebeuren. Dus als dat heel erg lijnrecht tegenover elkaar staat dan is dat op zich al interessant. Hiërarchie niet, maar ben wel benieuwd: hoe krijg je dat bij elkaar als dat erg uit elkaar ligt? Ik vind ouders wel heel belangrijk daarin, maar het kind uiteraard ook.
**2. *Met de informatie die nu gegeven is, zou je nu het probleem anders definiëren?***Dit is een boel meer informatie, bijv. dat er wel degelijk een probleem lijkt te zijn met schoolwerk, groep 3, hoewel dat thuis wel lukt. Er is een kinderneuroloog in beeld met een vorm van toevallen. Je zou het gedragsproblemen kunnen noemen, dat ouders tegen andere dingen aanlopen dan op school wellicht. Ik denk zeker als de hulpvraag zo helder is dan gaan we verder in kaart brengen wat er aan de hand zou kunnen zijn.
***Begrijp ik goed dat je op dit moment de behandeling zelf in handen zou willen houden?***Nee, ik zou nu denken aan doorverwijzen. Wellicht toch na overleg met de kinderneuroloog om geen dingen dubbel te gaan doen. Ik zou overwegen om eerst te overleggen wat de kinderneuroloog er van vindt en dan zou ik denken aan doorverwijzen.
***Naar wie zou je willen doorverwijzen, stel de kinderneuroloog is akkoord?***
Ik zou willen beginnen bij een basis-GGZ instelling of bij een orthopedagoog om eerst toch het hele verhaal in kaart te kunnen krijgen, dus dat moet wel een goede zijn. Ik heb nu wel iemand voor me die dat goed uit zou kunnen zoeken. Niet de praktijkondersteuner, ik zou wel meteen doorverwijzen met een goede terugkoppeling. Wat ik altijd bespreek met ouders is 'wat willen ze? willen ze een label of willen ze handvaten?' 'Wat is voor hen belangrijk?' En sommige ouders willen heel graag een labeltje, andere zijn er huiverig voor en dan bespreek ik ook met hen om dat eerst met een psycholoog te bespreken.
***Zijn er naast de basis-GGZ voor jou nog andere doorverwijsopties?***Als het meer 'het omgaan met' is en er nog niet zo duidelijk een vermoedelijke stoornis is dan zou je nog kunnen denken aan het kernteam: dus het JGT, voor opvoedbegeleiding.
***Is dat ook iets wat je in de dagelijkse praktijk doet bij een soortgelijke casus: naar het JGT doorverwijzen?***Hangt er een beetje van af, deze casus is wel complex. Als de vraag diagnostiek er ligt zou ik er niet mee beginnen, maar als het meer is: 'hoe zorgen we ervoor dat hij naar ons luistert en bij ons aan tafel blijft zitten?' - dan kunnen zij opvoedondersteuning bieden, maar ik zou het niet tegelijkertijd opstarten. Mijn eerste gevoel zou hier zijn: orthopedagoog of kinderpsycholoog om het eerst beter in kaart te brengen en dan pro memori het JGT. Het kan ook soms andersom zijn, dat wisselt per casus.
***Hoe zijn voor de rest je ervaringen op dit moment met het JGT? Hoe gaat , bijvoorbeeld, het overleg daarmee?***
Erg wisselend, het is een beetje wie je spreekt. Ik weet dat ze erg onderbemand zijn en dat er veel wisselingen zijn en dat er veel te doen is in het kernteam. Er is veel onrust geweest omdat er een aanbestedingsvraagstelling was: de gemeente had een nieuwe aanbesteding gedaan en de kernteammedewerkers zijn gedetacheerd vanuit verschillende instanties en bij die instanties was niet duidelijk of deze nog meegingen in de aanbesteding. Het was heel onrustig afgelopen jaar en vooralsnog ziet het er naar uit dat de hele aanbesteding blijft werken zoals het werkt, maar dat heeft wel mede ervoor gezorgd dat de communicatie moeizaam was hier en daar. Er was daarom veel uitval, veel zieken, veel wegloop was van de mensen van het kernteam. Het contact is erg wisselend, met het ene kernteamlid heb je makkelijk en goed contact en de andere vindt het lastig om een goede terugkoppeling te geven.
***Is dat op zo'n moment doorslaggevend om een patiënt door te verwijzen?***Als er niet echt een diagnostische vraag ligt maar meer gedragsproblemen, dan geef ik ouders de tip om -meestal als er geen acute nood is- zichzelf aan te melden, maar wel altijd met het bericht om het te laten weten als het niet lukt. Het wisselt een beetje hoe hoog de nood is of je er zelf achteraan gaat of dat je het bij ouders neerlegt.
3. ***Zou je het beleid willen wijzigen met deze nieuwe informatie?***Ik had de orthopedagoog van het JGT al gepasseerd; maar als deze orthopedagoog echt uitspreekt dat ze het vermoeden heeft dat er autisme speelt, dan zou ik Dave naar de specialist van het JGZ doorverwijzen. En ook omdat de situatie op school niet meer houdbaar is, dan wordt het wel prangend.

Casus 2 (Sanne):
1. ***Vind je dat er een probleem is? Hoe zou je het probleem willen schetsen?***Kennelijk geven haar driftbuien problemen binnen het gezin. Met name broer en zus hebben er last van. Moeder komt niet voor niets - denk ik - daarover praten, dus kennelijk is er een probleem. De vraag is aan moeder: wat is er al geprobeerd om er aan te doen? Hoe probeert ze daar mee om te gaan? Zijn ze daar al wel eens eerder tegen aan gelopen? Ik zou het weer uitdiepen: vragen wat de hulpvraag is, wat wil ze het liefst van mij?
***...en het in kaart brengen van verschillende opinies?***Ja, ik zou willen weten hoe het op school gaat: hoe zijn de contacten met mentor, of er maatschappelijk werk bij betrokken is of dat de driftbuien daar ook zijn. Ik zou zeker de andere terreinen uitvragen. Moeder komt nu alleen. Wellicht zou het interessant zijn om Sanne eens te horen. Maar dan zou ik wel eerst aan moeder willen vragen waarom ze alleen komt, of dat voor Sanne beter is - vaak zit daar een gedachte achter. Je probeert het plaatje wat helderder te krijgen. *2.* ***Wat zou je op dit moment doen?***Dit zou een meisje zijn om op dit moment voor te leggen aan het kernteam. Daar zitten ook mensen die vanuit de verstandelijke gehandicaptenzorg komen. Ik weet niet hoe benedengemiddeld de intelligentie is, dat speelt wel een rol hier: loopt ze op de tenen op school waardoor ze dit gedrag laat zien? Is het puur pesten? Waar komt dat vandaan en wat doet school er aan?'en het stukje van er mee om gaan: neiging tot schoolverzuim, ouders die niet op één lijn zitten - hier zou ik wel denken: laat iemand vanuit het kernteam dit maar 's goed in kaart brengen en hier ondersteuning aan bieden. Dit is toch wel vrij complex. Dit zou ik wel telefonisch overleggen met iemand van het kernteam om toe te lichten, aan te melden - misschien, als ouders daar in mee gaan.
***Was je dat aan het begin van de casus al van plan?***Ja, wellicht. Het zit wel meteen in je hoofd. Je vraagt toch wel eerst uit. Je kapt nooit het consult af met deze gegevens, maar dat heb je al wel in je achterhoofd. *3.* ***Hoe kijk je nu tegen de situatie aan?***
Ik vind het wel opmerkelijk dat school mij moet informeren over dat dit meisje meehelpt thuis. Is dat verhaal dan anders dan moeder hier vertelt, want waarom maakt school zich hier zorgen om? Moeder komt hier haar verhaal doen, vindt zij het problematisch als school mij gaat bellen? Dat zou ik wel even uitvragen. Ik vind het bijzonder als er heel veel weerstand bij moeder zit.
***Zou er nog een reden zijn om school uit jezelf te contacteren?***
Ik ben er zelf redelijk terughoudend in om dat zelf te doen. Als moeder aangeeft dat school mij wil bellen dan zou ik dat eerst even afwachten denk ik. Ik doe het zelden of nooit, eerlijk gezegd. Dan moet ik me echt zorgen maken over het gezin, maar je zit natuurlijk altijd met het privacystukje. Als het écht moet dan zou ik het bespreken, dus ik zou moeder vragen of zij het goed vindt dat ik met school overleg.
***Stel, school vertelt een heel ander verhaal dan moeder. Wat dan?***
Als er meer zorgen worden geuit over het helpen in huis denk ik wel dat ik even alleen met Sanne wil praten. Dan ga je daar toch weer opnieuw verder over praten, denk ik. Als ze inmiddels bij het JGT loopt, dan ga je daar ook weer mee overleggen.

Casus 3 (Melany):
1. ***Wat is je eerste indruk van deze casus?***Er zit wellicht wat relationeels tussen ouders en Melany, dat lijkt wel aan de orde te zijn. De vraag is wat het primaire probleem is: drinkt ze veel omdat ze drinkt of drinkt ze veel omdat het niet lekker gaat thuis? Een meisje van 15 wat vaak dronken is vind ik wel zorgwekkend. Hier gaan bij mij wel alarmbellen af. Er moet wel iets van begeleiding komen op zijn minst, niet alleen van ouders maar wellicht ook van Melany.
***In welke vorm zou je die begeleiding het liefst zien?***Ook wel weer het kernteam. Moeder komt echter nu alleen, dus je gaat eerst uitvragen sinds wanneer het probleem is, of moeder aanleiding ziet voor dit gedrag. Daarna zou ik Melany willen uitnodigingen. *...* ***Alleen?***
Ja, of soms doe ik het samen. Vaak zeg ik dan: eerst even samen een gesprek en dan alleen met het kind.
**2. *Wat zou je op dit moment besluiten?***Ik zou Melany zelf willen spreken, dus ik zou daar op aandringen. Ik denk nog steeds dat er begeleiding moet komen voor iedereen. Ik zou ook meer willen weten van de boze buien van vader: wat gebeurt er dan (schreeuwen? slaan?). Dit probeer je op een voorzichtige manier, zonder al te beschuldigdend te zijn, uit te vragen.
***Stel, ouders zijn het niet eens met observatie door het kernteam? Wat zou je op zo'n moment gaan doen?***
Je zoekt natuurlijk naar hulp die wel passend is, waar iedereen zich in kan vinden. Maar ik zou wel aandringen op begeleiding, zeker omdat Melany 15 is en dubbele toestemming nodig heeft. Een meisje van 15 dat steeds dronken is omdat het niet lekker gaat , dat vind ik zorgwekkend en dat gaat niet goed. In het ergste geval kan je zeggen dat het jammer is en dat - als Melany er zelf voor open staat - dat er dan hulp bij moet. Dan verwijs ik haar zonder toestemming van vader, desnoods. Dat kan, volgens mij, als je denkt dat het echt nodig is. Het beste blijft het om iedereen mee te krijgen.
***Stel Melany zou niet doorverwezen willen worden, zou dat het besluit nog anders maken?***Soms kost het wat tijd om wat op te bouwen en om iemand ergens naar toe te praten. Als het helemaal niet lukt, dan wordt het lastig. Ik zou toch proberen te forceren om hier hulp in te krijgen.
**3. *Zou je iets anders besluiten qua beleid met deze nieuwe informatie (t.o.v. 2)?***
De vraag is: waartoe is Melany te motiveren? Ze zit niet lekker in haar vel, dat moge duidelijk zijn. En de vraag is hoe ernstig is het? Ze is geregeld somber en ze piekert. Laag zelfbeeld klinkt hierin door, maar ook iets over erg boos kunnen worden. Anderen zeggen dat ze geen gevoel heeft, wat denkt Melany zelf dat ze daar mee bedoelen? Ik denk ook: is ze echt depressief? Als dat zo is dan gaan we geen contact zoeken met het kernteam, dan gaan we echt GGZ inschakelen. Verder: gebruikt ze nog andere dingen dan alcohol? Is ze aan het blowen? Ik heb nog wel heel veel vragen, als ik het zo lees. Afhankelijk daarvan zou ik haar doorverwijzen, of naar de specialistische GGZ of ik zou kijken of er iets lukt met inzet van het kernteam.

Algemene vragen:
***We hebben het gehad over je ervaringen m.b.t. het kernteam van het afgelopen jaar. Heb je a.d.h.v. deze ervaringen nog aanbevelingen of verbeterpunten voor het contact met het kernteam?***We zijn bezig om het contact met het kernteam te verbeteren, we werken ook al samen als huisartsen <naam regio> - dus daarin proberen we het contact te leggen en afspraken te maken. Zo zijn we bijvoorbeeld op dit moment bezig om een format op te stellen voor de terugkoppeling van het kernteam naar de huisartsen. We willen dit uiteindelijk ook gaan digitaliseren. Daarnaast spreekt een aantal afgevaardigden van de huisartsen <naam regio> op bepaalde momenten met vertegenwoordigers van het kernteam om praktische dingen af te spreken (bijv. zo'n terugkoppelingssysteem).
***Hoe zit dat met de specialistische jeugdzorg?***Op zich loopt dat wel. Er was een rechtzaak over de aanbesteding <naam specialistische GGZ-instelling> (werken we veel mee samen), die was de aanbesteding niet gegund. Dat is vooralsnog weer van de baan want de hele aanbesteding moet over. Daar merk je ook wel in de GGZ dat het allemaal met minder moet, dat past ook in het hele verhaal met die aanbesteding. Je merkt dat mensen weglopen, dus ze zijn wat onderbemand - merk ik. Mijn laatste contact was erg moeizaam, dat ik probeerde een crisisoverleg met de specialistische GGZ te plegen maar dat er niemand te bereiken was in <plaats in de buurt>. Uiteindelijk moest ik het via iemand in <stad in de buurt> doen. Het is hier en daar wel wat stroef. Dat mag wel verbeterd worden.
***Hoe vond je het interview? Heb je nog op- en aanmerkingen?***Ik vond het grappig om het zo even door te lopen. Het blijft altijd lastig om van papier een goede inschatting te maken. Ik denk wel dat je op zich een eindje komt met het helder krijgen wat je overwegingen zijn m.b.t. bepaalde besluitvorming. Je kan heel veel kanten op met zo'n casus dus het is wel belangrijk dat je als onderzoeker de vragen helder hebt - maar ik neem aan dat je dat ook hebt.
***Ja, haha (*laat vragenlijst zien*)***EINDE INTERVIEW

**Interview H2asi**Algemeen:
***Hoe lang bent u al huisarts in totaal?***Ik ben sinds 28 jaar zelfstandig huisarts, maar voor die tijd heb ik nog twee jaar in loondienst gewerkt, dus samen 30 jaar.
***Ervaren kracht, dus? En hoe lang werkt u dan hier in deze praktijk?***Ik zit hier 30 jaar in de praktijk. Niet in dit gebouw, maar in <naam plaats>.
***Alvast een beetje toespitsend op waar we het zometeen over gaan hebben: hoe vaak komt het nou waar dat u per week, per maand voor dat u een kind of jongere op het spreekuur ziet waarvan u denkt: nou, daar valt op psychisch of sociaal gebied het e.e.a. te verbeteren?***Welke leeftijdsgroep, wie is voor jou een kind?
***Tot 18, inderdaad.***
Tot 18. Ik denk zelf dat het twee keer per maand is. Als ik een persoon tot 18 jaar krijg, dan denk ik dat er iets meer aan de hand is of dat het mis kan lopen of het loopt niet helemaal zoals het voor mijn gevoel zou kunnen lopen.
***En die twee keer per maand, zijn dat dan nieuwe gevallen of ...***Ik bedoel meer dat er dan iemand met een ontstoken nagel komt en dan praat ik met zo iemand, dan kijk hij hoe hij komt, hoe hij gaat zitten, hoe hij eruit ziet, of hij verzorgd of niet verzorgd is/gekleed is. Dus ik praat meer over non-verbale communicatie.
***Oké, dus de hulpvraag is op dat moment anders?***Ja.
***En als u dan in gesprek gaat met zo'n kind of jongere, welke problematiek komt er dan naar voren. Waar denkt u dan aan?***Dan wordt normaal alles ontkend, alleen een concreet probleem wordt naar voren gebracht (ontstoken nagel).
***Dus niet dat ze daar dan veel over doorvertellen wat er achter zit?***Nee.
***En als ik het goed begrijp, komt het niet voor dat patiënten actief naar u toekomen om dit soort problemen te bespreken?***Misschien één keer per twee maanden.
***En wat voor problematiek is dat dan?***Verslaving, bijvoorbeeld, zich ongelukkig voelen.
***Somberheidsklachten?***Ja. Maar dan praten we over net geen jonge volwassenen, dus dan is de leeftijd ongeveer tussen 16 en 18.
***Dus een beetje de adolescentiefase?***Ja.
***Ik heb zojuist al kort met uw vrouw gesproken, die vertelde dat jullie wel een POH-GGZ hebben maar die ziet geen kinderen of jeugd?***Nee. Wij hebben in wezen 3 POH-GGZ, de ene mevrouw is rond de 40, ik heb een stagaire die klaar is maar dat verhoogt haar kans op de arbeidsmarkt (die is 30) en ik heb sinds kort een herintreder (van 63) - dat is een manager van een groot internationale kantoor, maar die gaat eerder met pensioen. Hij is van origine psycholoog, maar hij heeft nu weer de opleiding gedaan. Het is een soort droom van hem om later niet alleen nuttig te zijn voor internationale bedrijven maar ook meer voor mensen iets doen. Dat is een grote aanwinst voor deze praktijk.
***Dus dat is echt een soort carrièreswitch?***
Ja, omdat hij drie kinderen had. Hij is een zeer kleurrijk persoon. Door zijn levenservaring en kennis kan hij mensen ontzettend goed helpen, echt voor verschillende leeftijdsgroepen.
***Oké, dus ook over de leeftijdsgroep waar wij het over hebben?***Ja.
***Want hoe vaak komt het voor dat hij vanuit die leeftijdscategorie kinderen en/of jongeren ziet?***De echte kinderen die gaan allemaal richting GGZ, meestal. De echte kinderen zie ik niet zo vaak, dat is meestal via school opgevangen door schoolartsen. Dus als ik er aan te pas kom, dan is dat meestal om door te verwijzen.
***Dus dan ligt de vraag vanuit school eigenlijk al bij u om door te verwijzen?***Of bijvoorbeeld ouders die het niet eens zijn met school, ik zeg altijd ... met school niet vechten (?). Ik zeg altijd: ik wil een diagnose hebben, die wil ik zelf niet stellen dus dan verwijs ik ook. Ik ben van mening dat je met korte lijnen moet werken. Het kan niet zo zijn dat het kind verwikkeld raakt in een soort machtsstrijd tussen de ouders en de school en het kind is altijd de dupe.
***Daar komen we ook zometeen zeker over te spreken, ik wilde ook graag naar de casus gaan dus de dingen die u aansnijdt komen ook zeker aan bod. - uitleg casus -.***

Casus 1 (Dave):
1. 2. en 3. tezamen.
***Wat is de eerste indruk die u krijgt als u dit zo leest?***Het is een heel wisselend beeld, in eerste instantie denk ik dat het kind niets mankeert. Ik denk dat ouders waarschijnlijk toch, zoals dat vroeger met die Engelse films was (Nanny), het kind doen ontbreken aan reinheid en regelmaat en de grenzen. Hij is 6 jaar, voor sommige kinderen begint de school te vroeg. Ik praat als een vader van 4 kinderen, dus heb ik ook een bepaalde ervaring. Persoonlijk zie ik vaak dat, zeker jongens, veel te snel naar school gaan. Ik vind zelf dat kinderen veel harder moeten werken dan sommige ouders en ze missen daardoor het kind-zijn.
***Als ik het goed begrijp worden ze te snel met verantwoordelijkheden opgezadeld?***De testen beginnen vanaf het vierde levensjaar. Dat vind ik ongelooflijk, ongelooflijk ja. De kinderen krijgen zo snel etiketjes dus wordt theoretisch rekening gehouden met de ontwikkeling van het kind en praktisch niet. Ik voel mij gesterkt met mijn mening omdat ik zie dat de golf van ADHD'ers aan het begin van het schooljaar komt. Ik heb de indruk, ik weet niet of dat waar is, dat de leraren daar extra geld voor krijgen. Het is altijd voor mij frappant, waarom altijd aan het begin van een schooljaar?
***Waar krijgen zij dan precies geld voor?***Waarschijnlijk voor lastige kinderen of kinderen die meer aandacht krijgen. Dus dat is een heel lastige negatieve prikkeling, waarschijnlijk voor scholen. Maar ik weet niet of dat waar is, ik heb een flauw vermoeden. En wat zie ik ook in de loop van de jaren: ze worden steeds ouder. Hoe langer ik werk als huisarts, hoe meer ik die bij die kinderen opeens rond hun 25/26/27 levensjaar geen ADHD meer. Waar zijn ze?
***Dan zijn er opeens geen klachten meer?***Ja. Dus het systeem van scholen is.. ik ben zo ver nu dat eigenlijk -omdat je nu leest in de kranten dat jongens continu slechter presteren dan meisjes- dat eigenlijk het gemengde onderwijs een grote vergissing is. Jongens zouden echt apart moeten zijn, dat gaat niet over seksualiteit maar gewoon omdat zij anders zijn.
***Dus dat je echt weer jongens- en meisjesscholen krijgt?***Ja, ze zijn gewoon anders. Het is niet genoeg om een jongen te verkleden als een meisje en dan denken dat hij zich ontwikkelt als een meisje. Ze zijn gewoon emotioneel anders. Hier zou ik absoluut doorsturen voor een diagnose. Dat is een situatie... ik wil geen gevecht.. maar er klopt iets niet in dit gezin.
***En waar zou u naar door willen verwijzen, is dat ook weer de specialistische zorg?***Ja, voor de diagnose ADHD.
***Want zijn er nog zorgverleners waar u standaard naar doorverwijst, bijvoorbeeld vrijgevestigde kinderpsychologen?***Nee, ik heb alleen één psychiater - niet voor de diagnose ADHD - maar die kinderen hebben inderdaad problemen. <Naam vrijgevestigde kinderpsychiater> is een kinderpsychiater, maar die zou daaronder vallen.
***En waar werkt zij?***In <naam stad>, <naam vrijgevestigde kinderpsychiater>, het is een dokter afkomstig uit Suriname. Wat mij is opgevallen, wat ik in haar waardeer, ze houdt van kinderen. En dat hebben heel weinig mensen die houden van kinderen voor hun beroep. Dat lijkt misschien raar, maar dat ervaar ik (als ik de brief lees over kinderen), dat zijn mensen die eigenlijk niet in de kinderen geïnteresseerd zijn.
***En dat merkt u in de communicatie, als ik het goed begrijp, in de zin van: de brieven die zij dan terugstuurt. Zijn er nog andere aspecten aan dat contact wat heel prettig werkt?***Kwaliteit, die zijn open voor haar. Ouders hebben problemen met haar.
***Oké, wat melden zij dan?***Nou, ze is gek. Maar ik waarschuw de ouders altijd, dat is heel ingrijpend.
***Ik vraag me dan af: aan het einde van deze casus wordt het JGT genoemd, zegt dat u wat?***Ja, maar het probleem met JGT, ik had het afgelopen jaar een paar contacten wegens - dat was theoretisch dat het kind problemen had maar dat ouders in scheiding liggen - dus de ene ouder beschuldigde de andere dat deze de kinderen verwaarloosde. Die ouder is door de school benaderd omdat de kinderen werden verwaarloosd. Vader werkt als vrachtwagenchauffeur en hij vond dit en dat. Er kwam een onderzoek via die vader, die heeft de gemeente benaderd. Vader is niet mijn patiënt en de vrouw en de kinderen wel. De vader beschuldigde de moeder van de kinderen, zijn vrouw. Hier in de praktijk zijn er twee bijeenkomsten georganiseerd.
***Vanuit het JGT?***Ja, de ouders kwamen, drie personen van het JGT, mijn POH-GGZ heeft met de moeder 1x een gesprek per maand gehad en het bleek dat de vader al sinds een half jaar contact heeft met de gemeente en de conclusie na veel gesprekken van die persoon vanuit de gemeente (vanuit de JGT) dat de situatie moeilijk was. Dat is een heel doortastende conclusie na een half jaar *lacht*. Wij zaten hier letterlijk drie uur, ik heb expres onbeperkt tijd gemaakt. Na die twee gesprekken.. de conclusie was dat er een onderzoeksinstelling werd ingeschakeld. Bij het tweede gesprek was de directeur van school aanwezig, die zei dat er helemaal geen signalen bij kinderen... de kinderen functioneren prima *lacht*. Daarna nog 1 of 2 keer. Ik had gewoon geen tijd meer, mijn aanwezigheid was te tijdrovend. Mijn POH-GGZ ging er een aantal keer naartoe en hij zei ook: ze draaien constant rond. Hij is ook gewend vanuit het bedrijfsleven dat er vanaf een bepaald moment knopen worden doorgehakt. Dus dat is niets minder waar, het wordt alleen echt (?). Het zijn allemaal heel aardige dames in de leeftijd van 25 tot 65, die praten met elkaar gewoon gezellig.
 ***U mag casus 2 alvast doorlezen, hoor gezien de tijd.***

Casus 2 (Sanne):
1. 2. en 3. tezamen.
Nou, dit is moeilijk.
***Ja, dit is weer 's anders. Wat is volgens u nu het grootste verschil?***Nou, dat is inderdaad een zwakbegaafd meisje die net aan functioneert. Dat is duidelijk. En waarschijnlijk hebben ouders meer van dit soort kinderen, dus dat gaat waarschijnlijk ook niet zo goed. Waarschijnlijk hebben ze beide ook niet zo'n hoog IQ, dus dat is een heel moeillijke situatie. Het meisje moet inderdaad naar speciaal onderwijs.
***Er speelt meer door elkaar heen, begrijp ik?***Ja, en vaak het probleem is - hier speelt dat niet echt -, maar de ouders ontkennen vaak. ''We zijn toch niet gek'', maar hier lees ik dat niet. We weten dat het meisje laag functioneert.
***En als u dit zo ziet, hoe zou u dit aanpakken?***Het is al bekend dat zij een laag IQ heeft, dus via de gemeente bepaalde voorzieningen regelen om haar in een bepaalde richting te sturen. De vraag is altijd, maar dat is meer wat ik inwendig daarmee vecht (?), wat is goed? Vroeger, en die generatie is misschien allang uitgestoren, kwamen er boerenknechten hier en ze waren net niet zwakbegaafd maar heel laag funcionterend. De boer zorgde voor hen. We hadden hiervoor zelfs een man die vertelde dat hij door zijn moeder, toen hij 12 of 14 jaar was, bij de boer gebracht. Hij heeft zijn hele leven gewerkt als een boerenknecht. Toen hij 65 jaar was, heeft hij pensioen voor hem geregeld en daarna is hij naar een bejaardentehuis gebracht. Op zijn 65 (!) *lacht*, hier op dorp. Ja, maar dat was een jaar of 20 geleden nog zo. Hij vond dat de mooiste jaren, daarna, in dat bejaardentehuis. Hij had zijn kamertje, werd zo nodig verzorgd, had een scootmobiel. Hij had wel astma gekregen door zijn werk (met gooien enzovoort). Maar nu, het kind moet wel geleid worden omdat het meisje graag wil werken. Regelmaat, ze helpt haar moeder. Dat vind ik heel positief. Op een bepaald moment moet zij ergens naartoe gaan verwijzen, beschermd wonen. Ja, ouders hebben ook niet het eeuwige leeftijd. Dus als ouders onderbreken, dan moet een kind opgevangen worden. Ik heb ook een soort tragedies meegemaakt dat ouders hun kind totaal hebben beschermd, ze vielen weg en dan opeens was er een drama. Het kind was totaal niet aangepast aan de sociale maatschappij.
***Dus als ik het goed begrijp zou u in eerste instantie denken aan begeleid wonen?***Ja, met werken ook reinheid en regelmaat, dat is de basis. Op tijd eten, opstaan. Zoals hier ook trouwens staat: ze vindt het leuk, alles moet netjes en op een rijtje. Dat dwangmatige, maar het is prima.
***Interessante punten snijdt u aan. Want ik kan me zo voorstellen dat u voor ouders ook nog zorg zou willen regelen?***Ja, maar dat gaat automatisch. Dat is ook niet altijd goed, omdat ik ook twee gezinnen heb. De ene is bijna klaar, die krijgen voor kinderen die niet zoveel mankeren zgn. PGB. Ik dacht altijd dat ze weinig geld kregen daarvoor, nou dat had ik mis, want ze krijgen zeker 40.000 euro voor die twee kinderen. En die kinderen nu, is een drama, omdat kinderen al volwassen zijn. En de overheid heeft die PGB's vastgesteld. En de hele familie, met grootouders, leven van die centjes. Die kinderen kunnen niks, want ze worden altijd van grootmoeder van die en grootmoeder van die moeder en de tante die bezig zijn op de speelboerderij (?), dus die kinderen kunnen niks. En nu is het een drama omdat ze wel iets moeten doen, ze worden gedwongen door de <naam gemeente>. Maar die moeder is ''woehh'' want het inkomen is weg.
***Ja en de JGT's zijn ook het leven ingeroepen, dus de gemeente zegt van: gezinnen moeten het zelf regelen.***Maar voor mij, in dit concrete geval, heeft onze sociale maatschappij asociaal gehandeld. Dit is asociaal.
***Ja je zou zeggen, het is echt een probleem op grotere (politieke) schaal. Interessante punten, in ieder geval. Ik stel voor dat we naar de derde casus gaan. Deze is iets korter.***Casus 3 (Melany):
1.2. en 3. tezamen
Dit komt heel regelmatig voor, dit is de prijs van de scheiding.
***In het kader van verslavingen, waar u het zojuist over had?***Nou, het kind is totaal losgelaten. Ouders zijn gescheiden en zijn alleen bezig met henzelf, of vooral met henzelf. Ze zien kinderen niet of te weinig. Het is een losgeslagen kind. 1/3e van de gezinnen hier op de straat is gescheiden.
***1/3e? Dat is veel! Dat is ook hoger dan het landelijk gemiddelde.***Nee, het landelijk gemiddelde gaat naar 40%.
***Ongelooflijk.***Ja, en het kind is de dupe. Het is geen veilig nest. Ouders zoeken partners. Ik verbaasde mij vaak. Ik weet niet welke verwachtingen die mensen hebben, omdat als ze zijn gescheiden ze helemaal los gaan. Het is n**** als konijnen, meestal zie ik als zij nieuwe partners hebben, meestal zie ik dat ze genoegen met minder nemen t.o.v. hun eerdere partner. Ik weet niet hoe het is in <naam stad> of in andere steden, ik praat over het dorp. Het kind behandelt zichzelf, angsten, de sigaretten tegen depressie en de marihuana maakt je blij. Als zij als meisje sterk is, komt ze er uit. Als ze niet sterk is blijft ze altijd een soort slachtoffer.
***Ja, want u snijdt terecht aan dat ouders - zeker in zo'n echtscheiding - verschillende belangen hebben - ze vinden allebei wat anders. Hoe gaat u daar mee om, als ze hier op het spreekuur zitten?***Dan kijk ik naar de oudere die zich het meeste om het kind bekommert en dan ga ik die steunen. En zeggen hoe belangrijke hij/zij is, dat een veilig nest belangrijk is, verhalen als ''ik houd van jou'' - hele basale dingen, niks meer. Kinderen hebben betekent: geduld hebben, geduld hebben, geduld hebben - dat is de basis.
***Wat zou u doen in dit geval?***Ja, ik denk dat ik met het meisje zou willen praten. En ik praat met haar als een volwassene, maar ik ben geen psychotherapeut. Dus ik zou wel hulp aan willen bieden bij een psychologe.
***Bij de POH-GGZ?***Ja. En dat is afhankelijk van de moeder of vader die komt. Weet je, de persoon die komt heeft interesse voor zijn kind. Ik probeer hem/haar duidelijk te maken wat belangrijk is. En als ze met iemand willen praten zou ik ze doorsturen.
***Dus stap 1: u zou inventariseren, samen met de ouder die hier op het spreekuur komt en stap 2: de POH-GGZ en stap 3... aan welke hulpverlener zou u dan denken?***Nou, voor het meisje zou ik eventueel met die jongere psychologe of met die oudere man een afspraak in willen plannen. Omdat er dan een soort klik is, jongere mensen vinden sneller een klik met elkaar. Het is niet bedreigend, niet belerend.
***Interessante punten, heel goed. We zijn klaar met de casus. -uitleg wetenschappelijk onderzoek en vraag om tips-.***
Ja, ik denk dat er één contactpersoon moet zijn. Niet zo als bij het JGT die hier kwamen, er kwamen vier personen en ieder van hen had contact over een ander aspect. Vier mensen, vier meningen, omdat dit soort materie vaak afhankelijk is van hoe je persoonlijk naar het leven kijkt. Ik denk dat zelf dat er één begeleidende persoon nodig is en als die persoon het niet weet dan heb je nog die gemeentevergaderingen en ... hebben jullie advies (?) Maar niet iemand sturen waartegen alles weer moet worden verteld, die personen hebben geen tijd om zich te verdiepen in de papieren of in de voorgeschiedenis. Dat wekt bij ouders veel ontevredenheid, dus moet ik vijf keer hetzelfde verhaal vertellen.
***Dus één aanspreekpunt, één contactpersoon?***Ja.
***Dat is inderdaad een goede die ik mee kan nemen. Voor de rest nog op- of aanmerkingen?***Nee, want de dingen hangen van de politiek af. De politiek heeft iets besloten en verder heeft de gemeente daar ook niet zoveel invloed op. Maar, misschien ook zoiets, <naam gemeente> heeft in de aanloop naar die vergaderingen een paar keer bijeenkomsten georganiseerd voor o.a. ons huisartsen. En op een bepaald moment, de lijst van verschillende organisaties met wie de gemeente een contract heeft, voor begeleiding is eigenlijk ontzettend groot. Hoe kan je als gemeente overzicht hebben over de kwaliteit als je overeenkomsten hebt met 30 verschillende hulpverleners? Dat kan niet!
***Op een gegeven moment weet je niet meer wie je moet hebben.***Ja, en er is overlap continu. Kijk, we zijn allemaal verschillend en we zijn allemaal hetzelfde. Dus de problemen herhalen zich met verschillende nuances ook. Beter is om misschien met drie of vier organisaties, desnoods kunstmatig verdelen, jongeren en ouderen.
***Zodat het duidelijk afgebakende gebieden zijn?***Ja.
***Het zijn zeker interessante punten die ik mee ga nemen en uit ga werken. Ik zal ondertussen de opname stopzetten.***EINDE INTERVIEW

**Interview Hylb8**Algemeen:
***Hoe lang bent u al werkzaam als huisarts, als eerste vraag?***Ik geloof bijna 25 jaar.
***En hoe lang werkt u al hier in deze praktijk?***In <naam plaats> meer dan 20 jaar en in deze praktijk (deze praktijk is vrij nieuw onstaan na een dissociatie) sinds 3,5 jaar.

***Hoe vaak komt het nou voor dat u gezinnen...?***Bijna dagelijks.

***Werkt hier een POH-GGZ met wie jullie samenwerken?***Ja.

Casus 1 (Dave):
1. 2. en 3.

Er staat niet bij wat er verder wordt afgesproken, hè?
***Nee, klopt.***
***Wat is de eerste indruk die u krijgt als u deze casus zo leest?***Dat er hier sprake is van een probleem. En zowel thuis als op school, dus op meerdere gebieden. En dat zowel moeder als vader hierbij betrokken zijn, de school, Centrum Jeugd en Gezin en de hulpvraag van de ouders is: ''Wat heeft het kind?'' En het is onduidelijk wat de diagnose van het Jeugd- en Gezinsteam is en waarom ze een orthopedagoog hebben ingeschakeld. En ik ben benieuwd wat nu dus de hulpvraag is van moeder, als JGT er in zit.

***Als u deze casus zo leest, is dat vergelijkaar met...?***... Ja, dit is heel herkenbaar.

***U noemt al een aantal dingen, het verhelderen van de hulpvraag en ''waarom is JGT nu betrokken?'' Hoe vaak komt het voor dat u overlegt met het JGT?***Zelden. Ik verwijs naar het JGT en ouders kunnen zelf contact opnemen en sinds kort (ik denk een week of 6-8) krijgen wij bericht als iemand zich bij het JGT gemeld heeft. Daarvoor was dat dus nooit zo. En verder koppelt het JGT niet terug. Ik weet van heel veel mensen dat ze niet bij het JGT zijn, als er problemen zijn dan neem ik contact op met JGT want die beheren de geldstroom. En ''kom maar even terug als dat problemen oplevert en laat maar even weten hoe het afloopt''. En daarna hoor ik meestal niets meer.

***Wat vindt u daar van?***Nou, dat hoor je denk ik al.
***Haha, ja precies...***Dat vind ik niks. Het is zoals het is, voorlopig.

***Heeft u daar een verklaring voor, dat de terugkoppeling zo verloopt?***De JGT denkt, ten onrechte, dat zij privacy moeten beschermen en geen informatie kunnen delen met de huisarts. Heel soms krijg ik een mailtje of nemen zij contact op - maar dat doen ze meestal per mail - en dan is het heel bijzonder, dan is het per onbeveiligde mail waarin informatie aan mij wordt gevraagd als huisarts waar naam en toenaam, geboortedatum - de hele mikmak - over de gewone mail gaan. Daar ben ik dus ook niet blij mee. En dan stuur ik ze per beveiligde mail (via sleutelnet) een berichtje terug: met ''wat is jullie gerichte vraagstelling en willen jullie alsjeblieft niet via onbeveiligde kanalen allemaal privacygevoelige informatie gaan mailen?' Zo ligt het ongeveer.

***Ja, dus als ik het goed beluister: in de communicatie valt nog wel verbetering te behalen?***Ja, daar valt een hoop winst te behalen.
***Waaronder dus de beveiligde terugkoppeling, de beveiliging van patiëntgegevens.***
Ja.
***En neemt u deel aan bestaande initiatieven om die communicatie te verbeteren?***
Ja, ze zijn er wel druk mee bezig. Vanuit de huisartsengroep <naam gemeente>, en wij hebben vertegenwoordigers voor overleg met Centrum Jeugd en Gezin (want dat is er wel), vandaar dat ze nu ook sinds kort een berichtje sturen wanneer iemand zich daar heeft aangemeld. En er is in overleg met de gemeente, we verdelen de taken, anders zit je meerdere keren per week te vergaderen.

***Ja, dat snap ik. We hebben het nu dan zo over Jeugd- Gezinsteams. Nu richten wij ons ook in de onderzoeksvraag op specialistische zorg. Voorbeeld is <naam specialistische GGZ-instelling> in <naam plaats>. Hoe ervaart u die samenwerking?***
Daar verwijs ik naar en daar krijg ik goede brieven van terug. Maar dat is gewoon het specialisme. Dat werkt voor mij net als een ander specialisme.
***Dat gaat, zoals ik me kan voorstellen, via een doorverwijsbrief.***Ja, via ZorgDomein, alleen moet ik tegenwoordig wel in de gaten houden naar wie ik mag verwijzen om die ouders (voor de vergoeding). Het geld beheert de gemeente, voor een deel.
***U noemt al een aantal interessante punten, waardevol. Ik stel voor dat we naar de tweede casus gaan.***

Casus 2 (Sanne):
1. 2. en 3.

Hebben gedreigd de huisarts in te lichten.
***Klinkt bijna alsof het een soort politie-agent is***.
Haha, ja. Wat grappig. Nou hier speelt naar mijn gevoel heel erg veel. Wat absoluut van belang is is de beneden-gemiddelde intelligentie, een groot gezin, moeder ziek, financiele zorgen, een puber die heel erg -bijna op het dwangmatige af- gestructureerd is, met veel onrust en absoluut last zal hebben van de spanningen. Maar er is natuurlijk ook iets met Sanne.
***Als u dit zo leest, wat zou uw eerste plan zijn? - om het probleem verder in kaart te brengen of qua behandeling?***Dat hangt er een beetje vanaf wat moeder komt doen met Sanne. Maar laten we eens aannemen dat ze zegt: ''het gaat niet zo lekker met Sanne en...'' ja, wat zou ze kunnen vragen? Misschien wil ze wat hulp voor Sanne bij haar uitbarstingen, of zo. Ja, en dan hangt het er heel erg van af hoe Sanne er zelf in zit. Hier zou je kunnen verwijzen naar Jeugd en Gezin en kijken of er wat begeleiding kan komen voor moeder. Hier zou je ook kunnen verwijzen naar Sociaal Domein en er een maatschappelijk werkster op zetten. Je zou ook nog een inventarisatie kunnen laten maken bij POH-GGZ, zou ik hier niet voor kiezen. Maar wat ook nog een belangrijke is: ''school dreigt de huisarts te bellen.'' Dus: waarom? En wat is er op die school dan aan de hand, dus ik zou vragen of de school met mij contact op wil nemen. Omdat ik dan graag hoor wat hun vraag aan mij is.
***Dus als ik het goed begrijp: de informatie-inwinning vindt dus niet alleen plaats in het contact rechtstreeks met ouders maar u zou dan ook graag willen aansporen dat school als extern kanaal fungeert.***
Ja, ik ben wel heel erg benieuwd: wat is dit? Dit is bizar. Een school neemt nooit contact op met een huisarts. Deze regel springt er echt uit: dus waar denkt school aan en waar is school bang voor? En als dat zo is, wil school mij dan bellen? Ik zou dat het prettigste vinden om dat terug te spelen via Sanne en moeder, om de communicatie open te houden. Misschien hebben ze al een idee waar dit over gaat en anders zou ik school bellen, maar niet zonder medeweten van Sanne en haar ouders.
***U noemde zojuist kort de POH-GGZ en dat u deze casus daar niet voor zou kiezen, maar ik vraag me dan af: welke rol de POH-GGZ in (puur diagnostisch, of doet 'ie een deel van de behandeling?)***Nou, we hebben op het ogenblik een waarnemend praktijkondersteuner. Zij doet het zwangerschapsverlof en zij is nog niet heel erg ervaren. Ze doet wel een beetje diagnostiek met: ''is er distress of is er depressie?'' Maar ze verwijst heel snel, of ze doet wat ondersteunende gesprekken. Maar hier is meer nodig, dit is te specialistisch voor haar.
***Kunt u één kernwoord noemen waarvan u zelf zegt: dit verschilt echt van de eerste casus?***Het gezin. Ja, wat speelt er in dit gezin?
***U had het zojuist ook over begeleiding van ouders: in welk opzicht zou u die begeleiding willen geven?***Dat hangt er van af of ik die moet geven of dat zij daar zelf voor open staan, maar hier zijn nog heel veel vragen niet beantwoord. Vader en moeder zitten niet op één lijn, moeder heeft een chronisch probleem maar van osteopenie hoef je geen pijn te krijgen - dus wat speelt er daar? Wat zit er in de relatie? Wat gebeurt er als papa thuis komt? Waarom heeft dat kind driftbuien? Dit is aan alle kanten iets om uit te zoeken.
***Dit is een puzzel waarvan de puzzelstukjes nog gevonden moeten worden?***Ja, het eerste waar ik aan denk is: ''Ach, arme Sanne''. Dus, ja hoe gaan we het voor dit kind goed krijgen. Wat ook opvallend is, trouwens, is: school doet moeilijk dat Sanne mee-helpt, maar het lijkt erop dat Sanne juist het heel prettig vindt omdat ze heel erg structuur zoekt en graag klusjes doet. Dan wordt ze nog gepest op school, nouja: kortom, kortom, kortom: een heleboel.
***Mag ik het zo samenvatten dat dit echt een systeemprobleem is?***Dit vraagt systeemtherapie, absoluut. Ook, trouwens, de andere kinderen die niet genoemd zijn. Hoe gaat het met die andere vier? Het zijn er vijf. Met een enorm leeftijdsverloop: 15 en 1,5.
***Er is veel meer aan de hand bij meerdere personen?***Ja, dus Centrum Jeugd en Gezin want die behoren te weten hoe het met al die kinderen gaat. En die hebben misschien allang melding gekregen, misschien zelfs wel <naam advies- en meldpunt voor huiselijk geweld en kindermishandeling>-meldingen - je hebt geen idee als huisarts. Dat is jammer. Dat is wederom heel jammer.
***Ik begrijp het. Als u verder geen dingen toe te voegen heeft dan zou ik graag naar de volgende casus gaan.***Hier is een boel werk aan de winkel.

Casus (Melany):
1. 2. en 3.

Dit is ook een leuke.
***Haha, daar zijn ze op geselecteerd.***
Haha, ja.
 ***Welke indruk krijgt u als u dit zo leest?***Dit is een vastgelopen puber. Ik zou willen weten hoe die scheiding verlopen is. Het is opmerkelijk dat ze samen komen, dat gebeurt zelden. Maar dus nu heel erg goed, blijkbaar. Maar hoe was dat? Hoe is het gegaan? Dit is een puber waar je behalve aan alcohol ook aan drugs moet denken en aan pilletjes. Blowt ze? Slikt ze? Doet ze de hele mikmak? Die ontspoort en dan is er behoorlijke invloed vanuit de vriendenkring. Nou, dan heeft ze nog net de leeftijd dat ook Centrum Jeugd en Gezin misschien... die vind ik over het algemeen iets beter met jongere kinderen dan met de ouderen.
 ***Waar merkt u dat aan?***In de betrokkenheid, en logisch ook. Die kleintjes zien ze met regelmaat voor controles. Dus dan moet er echt een probleem zijn wil het gemeld worden. En dan, ja, hoe wordt het gemeld? Alleen als ik dus zeg: ''ga in ieder geval bij Centrum Jeugd en Gezin langs''. Tenzij dit bijvoorbeeld hele vermogende ouders zijn. Want die hebben we nog niet aan bod laten komen. Want als ze zeggen: ''heb jij voor mij iemand die dit goed op kan pakken?'' dan verwijs ik ze vrijgevestigd. Daar heb ik een aantal mensen voor in <naam stad> en in <naam stad> die ik en en waarvan ik weet wat hun kwaliteiten zijn. En de ene is goed met pubers terwijl de andere juist met kleuters geweldig is. Plus hun ouders, want dit gaat over systeemtherapie. En dan kies ik op grond daarvan. Want als ik naar Centrum voor Jeugd en Gezin verwijs dan moet je maar afwachten wie ze gecontracteerd hebben dat jaar, dus dat is een soort zwart gat. En als er sprake is van psychiatrie, dat is hier enigzins onduidelijk maar het zou kunnen dat dit een vastgelopen puber is met ADD of ADHD of - noem het maar op. En je wilt toch wat psychiatrische diagnostiek, dan verwijs ik naar <naam specialistische GGZ-instelling> en dan passeer ik Jeugd en Gezin. En dan denk ik: ''Oh, we hebben nu een psychiater nodig'' en <naam specialistische GGZ-instelling> is goed. De twee jeugdpsychiaters die daar zitten zijn prima. Dat is mijn hele spectrum waaruit ik kies van: ''Waar ga ik naar toe?''

***Dat is inderdaad wel interessant, met name dat punt over de vrijgevestigde hulpverleners waar u het zojuist over had.***
Ja, maar die moeten wel betaald worden. Sommige zorgverleners vergoeden het, meestal niet. Hier in <naam plaats> kan ik er niet heel veel gebruik van maken, maar ik doe het wel. Ouders hebben het er vaak wel voor over en ze zijn vaak met een aantal gesprekken sneller en beter geholpen dan wanneer ze eindeloze trajecten ingaan met eerst Centrum Jeugd en Gezin en dan de volgende en dan de volgende - en dan komen ze weer terug en dan gaan ze alsnog naar <naam specialistische GGZ-instelling>.

***Dus u heeft eigenlijk meteen: de patiënt en het gezin op de goede plek?***Precies. Kijk, dat vorige gezin, wat mij betreft als die geld zouden hebben, zouden ze naar <naam stad> zijn gegaan.
***Mag ik vragen hoe die hulpverlener heet?***<Naam vrijgevestigde GGZ-praktijk>.
***En hoe loop die terugkoppeling?***Bij de JGT loopt het heel problematisch. De kans dat zij contact met hen opnemen is heel klein. Wij bellen hen wel 's.
***En hoe gaat het met <naam vrijgevestigde GGZ-praktijk>?***Die kan ik gewoon op haar 06 bellen. En die belt mij. En daar zijn de lijntjes korter, dat werkt dus gewoon heel prettig. En ik hoor van die ouders terug wat zij van haar vinden en als dat niet klikt, dat gebeurt ook wel 's -hoor, dan is het ook meteen klaar. Dan bel ik 'r en dan zeg ik ''joh, dit loopt niet. Ga maar even door naar een ander.''

***En paar hele inzichtelijke punten, nogmaals. De vraag die ik ook al bij casus 1 heb gesteld, die herkende u heel erg vanuit uw praktijk (vanuit uw spreekuur). Hoe zit dat nou met casus 2 en 3.***
2 is de meest bijzondere en 3 is er ééntje die veel vaker voor komt. En 2 is de meest bijzondere omdat die heel erg complex is. Omdat er hier heel erg veel speelt.
***... dat systeem wat u zojuist noemde?***Ja, en inderdaad of je hier moet denken aan: ''is het systeem wel veilig?'' En de invloed van moeder: ''wat is het voor een moeder?'' Bijzonder dat Sanne knieklachten heeft terwijl moeder osteopenie heeft. Kortom: ik zie allemaal van die parallelelen.
***We hebben het zojuist gehad over doorverwijzen naar POH-GGZ, vrijgevestigde hulpverleners en dergelijke. Wat ik me verder nog afvroeg: in hoeverre blijft u zelf de ''hoofdbehandelaar?'' Waar trekt u voor uzelf de grens van: dit moet echt...***Schoenmaker, blijf bij je leest - is mijn motto. Ik ben dokter, ik ben geen psycholoog, ik ben geen pedagoog. Ik heb mensen die dat wel zijn en desgewenst wil ik wel de regiefunctie als huisarts nemen. Net zoals op somatisch gebied, dat je zegt: iemand komt bij vijf specialisten, is daar nou nog iemand die in de gaten houdt wat er eigenlijk gebeurt? Dat vind ik wel mijn taak, als huisarts. Maar ik ga hier geen relatietherapie doen, of ADHD-diagnostiek zelf doen. Ik ga wel het gesprek met die puber aan en ook met dat jongere meisje (Sanne), ik wil die kinderen wel zelf spreken - desnoods zonder ouders. Dat wel, maar dat heb ik nodig om goed te kunnen verwijzen en weten wat er speelt.
***Snap ik, want nodigt u dan...***Hangt er van af. Als ze samen komen vraag ik soms aan die moeder: ''vind je het goed, wil je even wachten want ik wil haar even alleen spreken.'' Of, ik vraag aan die moeder: ''vind je het goed, kom een keertje terug zonder Sanne - want ik wil jou graag alleen spreken.'' Dat hangt er vanaf: of als er maar één van de twee ouders zit, dat je zegt: '' is het een idee om een driegesprek te doen?'' Dat hangt er van af.
***We hebben zo alle casus besproken, we hadden het zojuist ook al een beetje de revue laten passeren: de knelpunten waarvan u zegt: de knelpunten met het JGT waar verbetering valt te behalen. Zijn er nog andere dingen waarvan u zegt van... want dit onderzoek - in het rapport wat uiteindelijk gaat verschijnen zal er uiteindelijk een wetenschappelijk verslag verschijnen. Ik wil daarin ook graag een aantal aanbevelingspunten of tips vermelden om de praktijk te verbeteren. Zijn er nou nog punten waarvan u zegt: ''Die moeten er echt in staan.'' - naast het contact en terugkoppeling?***Nou, wat ik heel erg belangrijk vind is dat je soms absoluut als dokter (als huisarts) aan psychiatrische problematiek denkt. Een depressief kind, een manisch kind, en weet je: het autisme en ADHD- oké, maar dan met name de wat extremere vormen. De kinderen die echt vast lopen, dat je denkt: ''ik wil een psychiatrische beoordeling.'' En dat vind ik, dat zou rechtstreeks van dokter naar dokter moeten - niet via het JGT. En dus zou het goed zijn als ze bij het JGT ook oog hebben voor psychiatrische aandoeningen. Vandaar: ''schoenmaker blijf bij de leest.'' Dat die kinderen niet te lang daar blijven rondcirkelen: bij orthopedagogen en dat zijn wel de uitzonderingen. Er zijn natuurlijk een heleboel kinderen die goed geholpen zijn met een paar gesprekken, maar mijn ervaring - helaas - is, is dat ouders vervolgens hier komen en zeggen: ''Ja, het consultatiebureau - want zo worden ze dan nog steeds genoemd - kan mij niet verder helpen.''
***Oke, dus: als er dus contact met het JGT wordt gezocht, dan zou u ook graag willen zien dat er ook is voor de psychiatrie. Dus dat zo'n kind niet te lang door blijft sudderen...***Het zou fijn zijn als er bij het JGT een kind wordt aangemeld, dat het JGT even zegt van: ''ga bij je huisarts langs om even te kijken of er medisch nog iets is - maar dus ook medisch-psychiatrisch.'' Dat zij het stuk van het gezin doen van adviezen en tips, bedplassen begeleidgen: prima, heel fijn. Maar graag de medische problematiek. En ook dit gezin dat moeder osteopenie heeft en het kind met knieklachten. Een leuke casus want er zit van alles in, eigenlijk, maar dat weet het JGT niet. Daar moet je dus als dokter naar kijken: ''wat gebeurt er daar nou, eigenlijk?'' Welke ziektewinst heeft moeder en hoe komt het dat het kind, behalve pesten op school, ook niet aan de gymlessen meedoet en: ''wat gebeurt hier?''
***Ik ben in ieder geval in dit half uur een hoop wijzer geworden, denk ik. Het blijft heel interessant zien om per interview het plaatje steeds verder ingekleurd te zien worden. Zijn er nog punten waarvan u zegt: ''die moeten nog besproken worden?'' of waarvan u zegt: ''ik heb alles wel gezegd.''***Volgens mij hebben we alles wel aan bod laten komen.
***Wat mij betreft ook, inderdaad.***

EINDE INTERVIEW

**Interview Hxc5r**Algemeen:
***Hoe lang bent u al huisarts in totaal?***Even denken hoor, ik ben afgestudeerd in juli 1984 dus dat is nu bijna 33 jaar.
***Ervaren kracht dus?***Dat mag onderhand wel, ja.
***En hoe lang werkt u al specifiek in deze praktijk?***Nu sinds 1 januari 1985, dus nu ruim 32 jaar. Er zat een half uur tussen waarin je klaar bent en dan moet je een praktijk gaan zoeken, dus dat half jaar heb ik waar genomen en totdat we hier konden starten in deze nieuwbouwijk (was het toen). Ik zit hier al geruime tijd, dus.
***En alvast een beetje toespitsend op het onderwerp waar we het zo over gaan hebben: over de jeugdzorg, natuurlijk. Hoe vaak komt het nou voor dat u kinderen en jongeren op het spreekuur krijgt waarbij u denkt: nou, daar is op psychisch of sociaal vlak wel wat aan te verbeteren?***Nou, dat is niet dagelijks maar dat is wel wekelijks. En daarbij heb je ook wel dat je gebeld kan worden door tegenwoordig het JGT of door een psycholoog of iets dergelijks waar mensen al naartoe zijn gegaan. Dus je hebt buiten direct contact ook nog een aantal contacten over het kind of het gezin, zonder dat je ze op het moment ziet.
***Indirecte tijd, dus?***Indirecte tijd, ja.
***En als ik mag vragen: wat ziet u dan het meeste in de praktijk?***Nou je hebt natuurlijk een groep jongeren die als ze naar school gaan, waarbij er dan door de school een aantal dingen opvallen. Dat kan zijn een verdenking op, veelvoorkomend, ADHD, of kinderen die zich absoluut niet binnen de groep kunnen functioneren; dat er gedacht wordt: zit het wat meer in het autistische spectrum? Dat zijn natuurlijk dingen die voorkomen op het moment dat die kinderen naar school toegaan. Vaak binnen het systeem van het gezin vallen dit soort dingen minder op, of ouders dat wat meer accepteren; totdat ze binnen een groep komen te werken - komen te functioneren.
***Interessant dat u dat zegt, want de casuïstiek gaat daar ook over zo direct. Ik stel voor dat we het huisartsenspreekuur gaan nabootsen. We beginnen bij de eerste casus.***

Casus 1 (Dave):
1. Helder.
***Relatief weinig informatie, maar zo begint zo'n spreekuur kan ik me zo voorstellen. Wat is de eerste indruk die u krijgt als u dit zo leest?***Meestal ken je het gezin wel, dus dan weet je wel een beetje hoe het in elkaar zit: of het een samengesteld gezin is of niet of dat Dave het enige kind is - de jongste, de oudste. Het is toch: dat geeft je een beeld hoe de sociale achtergrond is. Wat ik dan zou doen in dit geval is, hier in <naam plaats> heb je dat dan, het kind verwijzen naar het JGT met de vraagstelling: kunnen jullie nog wat dingen onderzoeken en contact opnemen met de school? En dan evt. kijken wat eruit komt.

***Dus het eerste wat in uw hoofd opplopt zou zijn: JGT mee laten kijken?***Ja. En dat hangt er ook een beetje van af. Als het allemaal dramatisch is, zoals dat nu Dave wordt (?) dan kun je je afvragen of dat nog zinvol is of dat je niet gelijk zou moeten verwijzen om met name te gaan kijken of er inderdaad aanwijzingen zijn voor ADHD.
***Wat ik net al vertelde, er is weinig informatie gegeven op dit moment: wat is de informatie die u op dit moment zou willen vragen aan moeder? Wat zijn dingen waarvan u zegt: die zijn belangrijk!***Nou dit is van school uit, dus wat moeder zelf thuis bemerkt; hoe dat de eerste zes jaar is gegaan - hoe hij op de peuterspeelzaal is geweest. Of hij uberhaupt op de crèche of dagverblijf is geweest. Of het altijd al de Dave is geweest zoals 'ie nu is, of dat er nu ineens een kentering is in het hele gedrag. Dat kan je natuurlijk zien aan een scheiding of iets dergelijks, of overlijden van opa en oma of iets dergelijks - dat zijn dingen die ik dan met name zou willen weten.
***Dus zoeken naar een knik in het verhaal?***Of er een knik in het verhaal zit of dat moeder zegt: nou hij is altijd zo. Nou ja, dan weet je dan dat hij zo is, of dan kan je wat minder naar allerlei andere achtergronden gaan kijken.
***Dat wat betreft het eerste stukje, dan stel ik voor dat u het volgende stukje doorleest.***

2. Dan ben je als huisarts snel uitgesproken, zal ik maar zeggen. Er is mogelijk een neuroloog een probleem (?), die toevallen of wat hij dan heeft. En natuurlijk een belaste familie met moeder met ADHD. En natuurlijk het gedrag wat hij vertoont, daar zou ik als huisarts natuurlijk niet zoveel mee kunnen doen. Dat betekent dat ik 'm nog 's gerichter zou willen verwijzen naar de kinderneuroloog, aan de ene kant, en aan de andere kant moet hij getest worden als hij geen ADHD heeft.
***Dus met name het stukje van de kinderneurologie is een reden om het beleid aan te vullen?***Nou kijk, er is iets neurologisch - het is niet normaal wat er gebeurt. Je wilt graag weten wat speelt daar een rol bij. Dat hij op 6-jarige leeftijd nog bedplast, dat kan. - KORTE ONDERBREKING -. Ik zou van de kinderneuroloog willen weten wat het kind nou eigenlijk heeft, is dat een vorm van epilepsie / zijn dat absences? Aan de andere kant zou ik 'm toch willen laten verwijzen om hem te laten testen of dat al dan niet ADHD is. Je hebt hier de DAWBA-test, daar kan je ook nog zien of het alleen ADHD is of dat het een autistische spectrumstoornis zou kunnen zijn. Dat zou ik wel willen doen, ja.
***Duidelijk verhaal in ieder geval.***

3. Dan heb je natuurlijk wel een probleem. Nou ja, dan heb je een probleem dat met de hele hervorming van de wet / zorgwet in de gemeentes en al die dingen dat je toch tegen wachtlijsten aan het oplopen bent.
***Dat is ook exact waar het onderzoek over gaat, u slaat de spijker op de kop***. ***U noemde zojuist al dat het JGT door uw hoofd ging. Hoe gaat die samenwerking?***Nou die samenwerking is op zich goed, maar het samenstellen van het JGT zoals het drie jaar geleden bedacht is dat blijkt in de praktijk dat niet elke JGT dezelfde expertise heeft. Wij zijn zelf, dat maakt mijn positie wat makkelijker, ADHD is iets waar ik wat mee heb. Drie jaar geleden hebben wij geprobeerd om een ADHD-straat van de grond af te krijgen omdat wij vinden dat die kinderen veel te veel door het hele land moeten (?) om een diagnose te stellen. Vaak twijfelen we aan de diagnose en vaak twijfelen aan de manier waarop er behandeld wordt. Wat we doen, in samenwerking met de JGT, de JGT's de DAWBA zouden doen. Maar dat bleek in de praktijk niet mogelijk te zijn, omdat niet elk JGT de expertise heeft en dan zou je een GZ-psycholoog moeten hebben die daar een klap op zou moeten geven. Nou die waren ook niet allemaal voorhanden, niet genoeg in begroot, dus uiteindelijk stierf ons plan een vroege dood. En nu ben ik als huisarts verbonden aan wat vorig jaar december is opgestart van <naam specialistische GGZ-instelling> en <naam specialistische GGZ-instelling>. En daar ben ik als huisarts, omdat het nog steeds mijn interesse heeft, verbonden. En kijk, daar werkt het gewoon wel goed, maar het is een pilot van <naam regio> en daar is te weinig geld in gestoken. En daar lopen nu ook de wachtlijsten alweer op.
***Dat is inderdaad één van de aanleidingen van dit onderzoek om dit in kaart te brengen.***
Ja, dus ik heb 1 mei al gesproken met diegene die erover gaat in <naam regio> en dat is een ambtenaar die over de centen gaat. Er komt donderdag in het <naam krant> een artikel over wachtlijsten en het hele gebeuren en daar heb ik een interview ook over gegeven. Dus dit is wel mijn stokpaardje, maar dat maakt niet uit want jij komt voor hele andere dingen. Nou weet je, hier loop je dus tegen aan en dan moet je ook veel druk gaan zetten op allerlei organisaties zodat die jongeren op tijd gezien moeten gaan worden.
***Nou in die zin is het juist interessant voor mijn onderzoek <uitleg promotietraject>. Alle informatie is welkom.***
Nou, sowieso dat er samenwerking is tussen <naam specialistische GGZ-instelling> en <naam specialistische GGZ-instelling> is al uniek, dat is echt voor de helft <naam specialistische GGZ-instelling> en voor de helft <naam specialistische GGZ-instelling> - nou die vochten elkaar de tent uit wie het slimste en het beste was. En, weet je, er wordt op een goede manier diagnose gesteld, iedereen moet een lijst invullen. En als je de gespecialiseerde DAWBA-deskundige die dat doen, is het multipathologie dan moet het naar de specialistische GGZ; is het mono (is het alleen ADHD) dan komt het bij het team terecht. Die hebben dan een groep voor de pubers, groepadviezen voor de ouders waar ze kunnen komen, er wordt gekeken of er evt. medicatie moet worden gegeven of niet. En als die kinderen ingesteld zijn op medicatie komen ze weer terug naar de eigen huisarts die dat dan weer verder gaat begeleiden. En zo zou het moeten.
***Zo zou het ideale pad zijn?***Zo zou het ideale pad zijn, en daar werken we nog steeds aan.
***Er is nog veel te doen, haha.***Ja, en dat heeft met name te maken met geld.
***Dat hoor je vaker natuurlijk, ook in andere takken van de geneeskunde maar met name nu in de jeugdzorg... Stel ik voor dat we naar casus 2 gaan.***

Casus 2 (Sanne):
1. 2. en 3. tezamen.
Dat is een lastig probleem.
***Heel ander verhaal, inderdaad.*** ***Wat is voor u het kernverschil met die eerste casus?***Enerzijds is het een benedengemiddelde intelligentie, die gaat naar een praktijkschool. En je hebt ook het idee dat het in dat gezin niet goed geregeld is, onderling. Dus dat het mogelijk een sociaal zwak gezin zou kunnen zijn, waarbij moeder vanwege haar osteopenie (is natuurlijk ook wel een heel raar ziektebeeld) in de Ziektewet zit. En dan zie je heel vaak dat er allerlei mechanismen gaan lopen die, nou ja je ziet: problemen met school, meisje gymt niet - nou iemand van 14 jaar dat is natuurlijk raar dat 'ie niet kan gymmen.
***Zou je niet verwachten...***Zou je niet verwachten, en dan krijg je natuurlijk dat dit een toenemend probleem gaat worden. Ze gaat niet naar school, buikpijn. Ik heb toevallig nu twee jongens van 14 die niet meer naar school gaan. En dat is niet echt een party om die weer naar school te krijgen. En dat is wel echt ingewikkeld. Dat is veel ingewikkelder dan casus 1. In casus 1 heb je vaak dat vader en moeder alles willen doen voor het kind. Hier willen ze dat ook wel, maar ze staan niet op één lijn en moeder heeft waarschijnlijk psychische problematiek. En het zal waarschijnlijk ook een kind zijn wat toch wel wat psychische problemen heeft.
***Zou ik het zo mogen samenvatten dat het eigenlijk multiproblematiek is?***Ja.
***Waar zou dit het beste thuishoren, wat zou uw plan zijn?***Nou, ik zou hier. Ik vind het een lastige. Kijk, in principe gaat het natuurlijk om het kind. Dus je moet kijken hoe kan je die Sanne weer gelukkig maken zodat ze weer fijn naar school kan gaan. Als 14-jarige moet je gewoon een leuke tijd hebben. Dus ik zou willen voorstellen om haar naar de algemene kinderpsycholoog te sturen om met haar te inventariseren: wat is er allemaal aan de hand en kijken hoe je daar achter zou kunnen komen hoe het nu werkelijk in het gezin aan de gang is. Vaak vertellen moeder en vader wel een verhaal waarin ze zich allemaal mooier voordoen dan dat het in het gezin rondgaat. Het is natuurlijk, maar zo ging het vroeger natuurlijk altijd, de bedoeling dat de oudste voor de jongste moet gaan zorgen. Maar dat is natuurlijk lastig voor een kind van 14.
***Ik kan me ook zo voorstellen dat dat de eigen ontwikkeling ook in de weg gaat staan.***Dat staat in de weg, en dan wordt je ook nog op school niet meer volledig geaccepteerd want dan wordt je gepest om wat voor reden dan ook. Nou dan wordt die school natuurlijk een steeds grotere drempel. Dan is buikpijn een soort ongrijpbaar probleem waarom je niet naar school hoeft te gaan. Het is heel lastig om daar te zeggen van: je hebt een buikpijn of je hebt wel buikpijn.
***U noemt het woord al: school. Die speelt in deze casus ook een belangrijke rol en die trekken aan de bel. Komt het natuurlijk ook nog wel 's voor dat u contact opneemt met school?***Nou wat je natuurlijk kijkt, want op die middelbare school heb je natuurlijk ook zo'n <naam schoolteam>, nou je kunt kijken of zij contact hebben met één van de vertrouwenspersonen op school. Nou in de beginperiode wordt dit vaak door school herkend en meestal is de schooldecaan van de onderbouw, die hebben meestal dat hij/zij de vertrouwenspersoon is. En dan moet met die gekeken worden die met haar contact kan krijgen of met haar in gesprek kan komen. Als dat lukt, dan heb je daar een ingang. Als dat niet lukt, omdat Sanne of omdat moeder dat niet wil, dan moet je dat even uit de schoolperiode trekken en dan zou je de kinderpsycholoog kunnen inschakelen. En als die dan wat verder is, dan kan je weer terugkoppelen aan school. En je moet kijken bij school of je dat verzuim zou kunnen aanpakken. En daar hebben de JGT's tegenwoordig ook wel ervaring mee. Die hebben een speciaal team die regionaal werkt om schoolverzuim aan te pakken.
***Als ik het goed begrijpt, het JGT zou hierin ook...***Die zou hierin een rol kunnen spelen. Maar in dit geval moet gekeken worden naar wat school maximaal kan doen en aan de andere kant moet je kijken hoe je Sanne kan ondersteunen in dit hele proces - en dat kan de kinderpsycholoog doen.
***En is dat dan een vrijgevestige kinderpsycholoog?***We hebben hier veel contacten met <vrijgevestigde GGZ-praktijk>, dat werkt in <naam plaats> en je hebt in <naam plaats> een vrijgevestigde kinderpsycholoog. Hier in <naam plaats> hebben we dat niet.
***Misschien is dit een gemene vraag hoor, maar de samenwerking zoals u die met het JGT heeft, hoe verhoudt die zich tot de samenwerking die u heeft met de vrijgevestigde hulpverlener? Zitten daar verschillen tussen?***Eh, dat weet ik eigenlijk niet. Ik weet het niet. Ik denk dat die wat minder is dan dat wij er mee hebben. Dat kan ik niet bewijzen.
***Duidelijk, wat betreft casus 2. Dan stel ik voor dat we naar casus 3 gaan.***Casus 3 (Melany):
1.2. en 3. tezamen
Je hebt makkelijke casussen gekozen.
***Haha, ja ze zijn overigens opgesteld in samenwerking met een orthopedagoog.***Dit is helaas een veelvoorkomend probleem.
***Het meest voorkomende ook van de drie?***Nou ik denk dat ze alledrie wel voorkomen, je hebt heel veel opgebroken gezinnen. Helaas en heel veel met jongere kinderen, deze is dan niet meer zo jong, maar in die kwetsbare levensfase is het een groot probleem. Die vader en moeder gaan natuurlijk niet voor niets uit elkaar. Daar zit natuurlijk allerlei redenen in (?) waarom ze uit elkaar gaan. Dan zie je dat die pubers vaak ook misbruik maken van de situatie, dus overal hun voordeel proberen te halen. Die proberen ze een beetje tegen elkaar uit te spelen en daar een eigen gang in te gaan. Als je 15 bent wil je het liefst niet gecontroleerd worden. En als zijn al meer volwassen is dan 15 jaar, dan ontstaat dit. Op zich is ze een slim meisje die, als ze er zin in heeft ook wel goede cijfers houdt en als ze er geen zin in heeft en het niet doet dan is het gewoon helemaal niks.
***Daar snijdt u ook een belangrijk punt aan. Ik vraag me dan af hoe dat dan gaat in uw spreekkamer, dat ouders tegelijk komen en er is dan toch een belangenverstrengeling. Hoe pakt u zo'n consult aan?***Ik probeer in ieder geval, degene waar het om draait is Melany. Dat zeg ik dan ook altijd. Het is goed dat je komt, maar het gaat mij om Melany. Het kan ook betekenen dat ik het soms met de een of met de ander niet eens zou kunnen zijn. Dus dat probeer ik wel vanaf het begin duidelijk te maken, om te voorkomen dat ze je als een soort bondgenoot overal bij betrekken. Ze komen de eerste keer samen en daarna en dan wordt je apart elke keer wel weer bestookt. Omdat het dan bij vader niet goed gaat en de andere keer dat het dan bij moeder niet goed gaat. Dus daar moet je wel vanaf het begin een beetje je weg in zien te vinden. En ik hoor dan die vader en moeder aan en probeer dan een beetje te inventariseren. Als het een school is, meestal een school in de buurt, kijken of ik daar contact mee (?) en dan wil ik gewoon Melany spreken.
***Maar als ik het goed begrijp, dat gemeenschappelijke uitgangspunt bereiken bij beide ouders: Melany staat op één. Dat probeert u te bewerkstelligen.***Ja, bij ons, mijn collega en ik hebben vaker problemen met kindermishandeling en dat soort dingen. Je moet altijd duidelijk maken naar die ouders dat het kind op nummer 1 staat, daar gaat het gewoon om. Die heeft nog een leven voor zich en hoe minder beschadigd ze door die puberteit komt, hoe beter. Het is een slim meisje die straks ergens gewoon op een veel lager niveau terecht komt en dat ze er spijt van heeft dat ze dat niet volledig benut heeft - en dat is gewoon zonde. Dus ik zou in ieder geval willen uitnodigen om haar een keer apart te zien.
***En als ik het zo goed beluister is het dan met name iets wat u zelf zou willen...***In eerste instantie zou ik van haar willen weten: hoe zit het nou? Zou ze daar hulp voor willen helpen? Je zou via die ouders hulp kunnen aanbieden, maar als zij dat niet ziet zitten dan gebeurt het gewoon niet. Dan maken die ouders een afspraak bij de kinderpsycholoog, ja als ze 15 jaar zijn en ze heben daar geen zin in, dan gaan ze gewoon niet.
***Ja, het is natuurlijk een halve volwassene, een volwasssene in wording.***Ja, zeker. En wat je dan vaak ziet is dat die kinderen van gescheiden ouders, zeker in die leeftijd, wat eerder volwassen zijn dan - niet allemaal - door alles wat ze meegemaakt hebben.
***Daar ben ik persoonlijk wel benieuwd naar: hoe denkt u dat dat komt?***Nou, die ouders gaan uit elkaar. Vanuit het veilige huis waar ze eerst zaten moeten ze dan om de week naar een ander huis toe. Nou, soms blijft het ouderlijk huis intact maar soms worden het twee nieuwe huizen dus je moet ook nog je plek vinden. En met een beetje pech zit je ook nog in twee dorpen of steden. Dus dat betekent dat je aan alle kanten je vrienden en sociale contacten verliest. En één van die twee krijgt een nieuwe partner, dus die zal ook wel weer z'n invloed willen hebben. Dus dat maakt dat die kinderen toch wel eerder volwassen worden.
***Er wordt eigenlijk een beroep gedaan op hun copingmechanisme?***Ja.
***Wat mij betreft waren dat alle vragen wat betreft de casuïstiek.***Algemeen:
***-Uitleg artikel en streven naar aanbevelingspunten/tips-***
Nou, de jeugzorg zit bij de gemeente. Daar is voor gekozen dus dat is gewoon goed, dat moet zich ontwikkelen. Ik denk dat het heel erg belangrijk is dat de expertise in de JGT's, dat dat gewoon echt heel goed gemonitoord moet worden. Want <naam van een coöperatieve vereniging van 20 regionale organisaties, die zich inzetten voor mensen met een beperking en hun netwerk>, <naam specialistische GGZ-instelling>, <naam specialistische GGZ-instelling> moeten overal mensen leveren en ik vraag me af of de goede expertisemensen daar terecht zijn gekomen. Want ze worden betaald, ze krijgen geld vanuit <naam regio> om die mensen te leveren. Daar zou ik eigenlijk wel een toetsing op willen hebben, of er voldoende kwaliteit is. Met name kwaliteit waar je dit soort dingen mee kunt doen. Als je het goedkoper wilt hebben straks, dan moet je zorgen dat je binnen een buurt of gemeente heel veel expertise in huis hebt. En dat is de gedachtegang geweest achter het JGT, dus je moet iemand hebben die in ieder geval ervaring heeft voor ADHD of ADD of iemand met alle vormen van autisme die in ieder geval een soort begininventarisatie zou kunnen doen, waarmee je voorkomt dat mensen onnodig naar de specialistische GGZ gaan en veel meer de expertise binnen de gemeente zou kunnen houden. Want er wordt naar mijn mening nog veel te weinig op gestuurd. Kijk, de JGT's worden volgestouwd met allerlei mensen van allerlei organisaties, maar de vraag is of er iemand is die duidelijk gaat omschrijven waar het JGT aan moet voldoen. En ik heb het gevoel dat dat gewoon iets is waar veel te weinig op gestuurd wordt. Omdat je expertise in huis hebt, dan kunnen de scholen er veel beter op sturen. Want scholen weten vaak ook niet wat ze ermee moeten.
***Toch dat je op één of andere manier die kwaliteit kunt waarborgen.***
Ja, er is een organisatie opgesteld, dat zijn de JGT's. Daar moet iemand die ervaring heeft met scheidingen. Er moet iemand inzitten die ervaringen heeft met ADD en ADHD en met vormen van autisme en voor de oudere pubers met verslavingen van drank en drugs.
***Ja, dus dan zou je een soort universele standaard moeten hebben waarin de meest voorkomende problemen, dat daar de goede expertise voor is.***Ja, en dat die ook weten waar ze naar toe moeten.
***Hele goede invalshoek, die ga ik zeker meenemen. Ik zal de opname nu stoppen.***EINDE INTERVIEW

**Interview Hn7iz**Algemeen:
***Naam?***<Naam huisarts>.

***Hoe lang ben je al werkzaam als huisarts?***Als huisarts sinds maart 2014.

***En ook meteen hier begonnen met werken?***Ja.

***En hoe vaak in de week behandel of zie je per week gezinnen met psychosociale problematiek?***Wat is je definitie van psychosociale problematiek is dan van belang. Ik denk dat er maar heel weinig mensen echt specifiek voor psychosociale problematiek hier komen, maar je ziet ook heel veel kinderen met buikpijn of met ADHD of ''school zegt'' en dat is wel meerdere keren per week. Dat zullen ze zelfs niet altijd als psychosociale problematiek benoemen.

***Dus ze komen met een andere klacht...***
... Ja en dat je dan denkt aan: is er iets anders aan de hand?

Casus 1 (Dave):
1. ***Wat is de eerste indruk die je hebt, stel je zou dit op je spreekuur tegenkomen?***Een deel zou ik vragen en een deel gedachten. Mijn eerste gedachten zijn: is het op school getest? Heeft school al wat gedaan er aan? Is patiënt bekend met dergelijke problematiek, in de voorgeschiedenis of in de familie (is het een terugkerend fenomeen)? Dat soort dingen vraag ik me dan af. Twee is: we gaan altijd na of het alleen op school speelt, zijn er problemen thuis, zijn er problemen op de sportvereniging - dat soort zaken. Dat soort dingen wil je weten en ervaren de ouders zelf ook een probleem? Soms zie je wel 's dat school problemen ziet waar de ouders dat niet zien, dus at probeer je een beetje uit te zoeken.

***Beperkt zich dat dan tot het consult zelf: dat je vraagt aan ouders/verzorgers: wat vindt school er van? Of ga je er actief achteraan (naar school toe)?***
Nee, de ouders en kind zijn op dat moment doorgevend voor de situatie.
***Die zijn leidend, zeg maar?***
Ja.

2. ***Met de informatie die gegeven is, heb je al een beeld van wat je hiermee zou willen doen?***
Bepaalde dingen zijn nog niet helemaal duidelijk, bijvoorbeeld: thuis maakt hij zijn schoolwerk in een paar minuten af - is dat goed? Doet hij dat op goed niveau? Dat is een beetje de vraag, als dat zo is is dat geruststellend. En die toevallen (dat uit het niets wit worden en gaan overgeven), daar is nog niet zoveel uit gekomen denk ik, maar dan wil ik dat ook wel even weten.
***Hoe zou je dat aanpakken, zeg maar?***Nou, waarschijnlijk is er een brief van de kinderneuroloog, dus daar zou ik even naar kijken. Er zal wel voorwerk gedaan zijn, hij zal wel niet voor niets naar de kinderneuroloog gaan of een bekend verhaal zijn. Het is niet dat ik zelf nou enorm aan epilepsie zou denken, maar er speelt meer schijnbaar. Wat ik niet zie in hoeverre ze hem kennen. Ouders herkennen zijn eigen gedrag in Dave. En het wordt mij ook niet zo helder, hij stoort kinderen wel in zijn gedrag, maar hoe hij sociaal functioneert. Dus: heeft hij vriendjes, hoe is hij op school, is er pestgedrag? Dat soort dingen.
***Als ik het echt ook over punt 1 genomen zou mogen samenvatten, zou je een globaal willen schetsen van wat er aan de hand met Dave en wat is de opinie van...***Ja. Kijken we nou meer naar een gedragsprobleem, een leerprobleem of praten we hier over psychosociaal probleem? Het één heeft met het andere te maken, ik denk dat dat een valse scheiding is, maar gedragsproblematiek (ADHD of autisme), kijken we meer naar een leerprobleem (hij kan op school misschien niet zo goed mee, dat soort dingen) en of is er sprake van dat hij gepest wordt of dat er thuis problemen zijn. Daar wil je dan naartoe.
***Stel: uit het consult blijkt dat ouders niet zo happig er op zijn informatie te geven. Je merkt dat de informatie die je wilt verkrijgen via ouders niet zo goed lukt. Hoe zou je het dan aanpakken?***Dan stuur ik ze al vrij snel naar de POH-GGZ. De POH-GGZ zoekt wel actief contact met de Remedial Teacher of met de school. Die hebben allerlei vragenlijsten die mensen dan thuis kunnen invullen. Dat is dan de volgende stap.
***Dus je laat de POH-GGZ echt voor de diagnostiek meekijken?***Ja, die laat ik 'm in kaart brengen, zeg maar.
Grofweg gezegd, als huisarts hier moet ik een grove screening doen: is dit iemand die naar de JGZ zou moeten, is het iemand die naar een psycholoog zou moeten, of naar het JGT of naar een opvoedbureau (bijv. bedplassen, misschien moet je daar gewoon eens langs) of is het iets wat binnen een heel beeld past - is het een groter probleem, dan moet je naar het <naam specialistische GGZ-instelling> of weet ik wat, of is het gewoon onduidelijk en dan is de POH-GGZ die het dan in kaart brengt.
***Oke, precies, dus jullie hebben echt een soort van stappenplan hiervoor?***Ja.

3.
Dat is in die zin intrigerend, want dan ben je natuurlijk benieuwd wat er uit die vragenlijst komt.
***Je hebt een duidelijk beeld geschetst van wat jullie als huisarts hier gewend zijn om dit soort problematiek te tackelen. Wat ik me verder afvroeg: je hebt het over JGT en over gespecialiseerde zorg <naam specialistische GGZ-instelling> - dat past ook in onze onderzoeksvraag: hoe gaat het contact met de JGT danwel met <naam specialistische GGZ-instelling> of andere gespecialiseerde zorginstanties?***We zijn een beetje aan het opstarten. Toevallig hebben we hier een soort pilot gedraaid waarin ze hier ook spreekuren samen meedoen. Dat is niet een heel groot succes gebleken, maar we hebben er wel een poging toe gedaan. In ieder geval is daar ook via de mail een stuk makkelijk om contact te hebben en telefonisch zo nodig maar je merkt wel dat ze een beperkt aantal dagen werken. Ik werk zelf 4 dagen, maar zij 2 of 3 dagen. Dan merk je al heel snel dat het lastig is dat je 1 of 2 keer probeert en dan verzandt het weer, dan spreek je elkaar niet. Dat is een aandachtspuntje.
***En waar liepen jullie tegen aan tijdens die pilot?***
De problematiek die je voor je krijgt, dat mensen niet helemaal met het beeld van 'nou dat is echt een JGT-probleem'. Ik denk dat wij echt anders werken dan JGT en dat de patiënt met een ander beeld voor ons zit. Het JGT gaat toch nog wel meer uit van in principe normaal gedrag, en dat moeten we een beetje bijsturen dus dat komt wel weer goed, terwijl de patiënt bij ons komt met de opmerking: het gaat echt niet goed. En op het moment dat ze openstaan voor (?) dan zie je dat het best wel ver weg is. Bij deze (casus) ook hè, dat je al bij het begin zegt dat iemand bij het JGT is gekomen dan (?) 'Oh da's mooi, dan zal ik straks 's met haar overleggen'. Maar dan weet je natuurlijk niet wie het is, en dan.. Maar dat komt eigenlijk niet voor.
***Dat is wel een interessant punt wat je daar aansnijdt, het klinkt bijna alsof het JGT een minder ernstig beeld heeft van de zaak dan de urgentie die de patiënt hier presenteert.***Ja, als ze hier zijn. Het JGT zit ook in de nulde lijn, meer. Dus ja, dan is het ook logisch dat ze wat normaler beeld hebben. Ze zullen ook niet alles aanpakken. Ze doen geen psychiatrische problematiek, direct, maar meer de omgeving dan. Er zitten bij ons veel meer: is dit autisme of is dit niet ADHD? Als ik aan ADHD denk, denk ik niet aan het JGT. Ik denk: ''ja misschien zitten er wel orthopedagogen, die kunnen die vragenlijst ook doen maar die hebben ook wel betere dingen te doen. Het is niet echt hun taakgebied. Op school is het zo, volgens mij heb je op school begeleidingsdiensten die dingen doen (middelbare school), maar ze hebben ook volgens mij budgetten voor de basisschool. Dat is weer een andere club dan het JGT. Daar krijgen we ook niet precies een vinger op.
***Je had het erover dat je contact hebt met het JGT in de vorm van mail, hoorde ik zojuist. Hoe gaat bijvoorbeeld die terugkoppeling?***Wij vinden het wel fijn als we weten dat ze daar bij zitten. Dat zijn ze ook wel van plan, begreep ik. Sinds we dat afgesproken hebben heb ik ook geen één patiënt meer gehad die bij hun is, dus misschien loopt dat wel dus dat weet ik niet. Pas hebben we een overleg gehad met <naam orthopedagoog>. In die zin, omdat ik <naam orthopedagoog> ken is het ook wel makkelijk.
***De lijntjes zijn kort, hoor ik (haha)?***Ja, al merk ik dat het bij anderen zo is dat het ingewikkelder is, maar er is wel wat meer goodwill in de zin van: we hebben ook wel wat aan elkaar.
***...we hebben elkaar nodig...***Ja. Je krijgt snel parallele paden. Tot nu toe was het contact veel vaker dat ouders ontevreden waren over het JGT en dan kwamen ze weer bij ons en dan wilden ze toch naar het <naam specialistische GGZ-instelling> o.i.d. Maar, zij zitten ook in die club. Dus de huisarts was dan een sluiproute om toch je ding voor elkaar te krijgen. Dat is altijd wel een beetje een risico: dat mag wel, maar dat is wel een probleem soms.

Casus 2 (Sanne):
1. 2 en 3. Deze casus komt mij bekend voor, die is namelijk besproken op die bijeenkomst (met <naam orthopedagoog>). Dit is wel echt een JGT-ding, natuurlijk. Want hier ga je natuurlijk richting Kristal-problematiek, lagere intelligentie. Ouders met beperkte capaciteiten, dat zal wel echt heel erg zijn. En dat heeft allerlei consequenties die Sanne niet allemaal zelf kan oplossen omdat ze beperkt is en het gaat er ook om hoe we met elkaar afspraken maken. Hiervan zou ik zeggen: deze zou ik aanbevelen omdat deze al die domeinen in zich heeft.

***Je noemt al heel kort moeder met osteopenie. De casus die zich nu zo schetst, is dat dan meer patiëntspecifiek of zeg je 'dit is meer een systeemprobleem?'***Een systeemprobleem, natuurlijk. Een kind is dan natuurlijk een uitgangspunt, dat is de alarmbel. Die wordt misschien overvraagd. Die is de oudste, misschien moet ze wel allerlei dingen doen die voor die andere kinderen helemaal niet lukken. Of je zegt: hier zit de GGZ <naam kinderpsycholoog> die doet ook altijd het systeem erbij pakken, dat zou een andere optie zijn omdat te proberen. Het IQ is toch wel echt laag, dus Kristal, die zit ook in dat team dus dat is makkelijk.
***Want voor mijn begrip, wat doet Kristal dan precies?***Kristal is voor mensen met een IQ onder de 80, die doen de begeleiding. Dan moet je wel een IQ test hebben gehad.
***Je hebt het over dat het een systeemprobleem is. Welke zorg zou je dan voor ouders bijvoorbeeld willen bieden? Zou je daar wat mee willen doen of zeg je van: 'nou, ik wel met name nu Sanne begeleiding bieden?'***In onze praktijk zou ik het benaderen van als een soort pappen en nat houden. Het zijn mensen die regelmatig langs komen. Dat mag. Die probeer je dan een beetje te steunen. Die komen met alle kinderen dan af en toe eens langs. En zo probeer je ze een beetje in de gaten te houden maar ik zou ook niet zo goed weten hoe... ze laten zich vaak ook niet zo heel makkelijk helpen. Ja, vertrouwen houden en langs laten komen.

Casus 3 (Melany):
1. 2. en 3.
Dit is een dramatische vraag, natuurlijk.
***In welk opzicht verschilt de problematiek in deze casus van de voorgaande casuïstiek?***
Ja, dat er gescheiden ouders zijn, boze buien. Botsingen, er wordt gedronken op 15-jarige leeftijd dus er zijn wel wat veiligheidsaspecten die hier een rol spelen. Hoe ontaardt dit niet?
De vorige casus is ook iemand die op haar niveau zit en met haar frustraties. Bij deze dame heb ik het gevoel dat bij deze dame er meer uit was gekomen als er meer leiding was gekomen in haar leven.
***Door wie precies (lijn)?***Door ouders, als zij misschien in een gezin was opgegroeid waar haar ouders niet gescheiden waren, waar vader niet zo boos reageert had zij in een betere structuur een heel stuk makkelijker gefunctioneerd.
***Dus als ik het zo mag samenvatten: is het ook hier een systeemprobleem?***Ja.
***En zou je plan van aanpak verschillen bij deze casus ten opzichte van die andere casuïstiek?***Ja, je vraagt wel veel informatie - die krijg je niet altijd, trouwens. Op zich zou je hier al snel naar de POH-GGZ sturen, die heeft het meeste netwerk hiervoor. Als ik naar mezelf kijk is het natuurlijk wel een beetje lastig omdat je van school wel de informatie krijgt, daarom zeg ik dat is vrij bijzonder want die krijg je vaak niet (van de mensen zelf). Waarschijnlijk als ik dit zo zie is er vast wel iets ondernomen, kan me niet voorstellen dat hier niet met een schoolbegeleidingsdienst of met een JGT al niet contacten zijn geweest. Daar zou ik zelf een beetje op voortborduren. Ik zie geen somatische problematiek, dus het is niet zo dat ik zeg: die moet nu naar die dokter toe. Ik zie wel een begin van GGZ-problematiek (alcoholgebruik, onrust, piekeren) en als je weinig voortgang ziet, dan zou ik toch hier individueel voor Melany hulp inroepen.
***En in welke vorm zou je die hulp inroepen?***In eerste instantie POH-GGZ omdat die ook makkelijk met allebei de ouders het gesprek kan aangaan en ook de problematiek in kaart kan brengen. En eventueel via hen dan naar een psycholoog ofzo. Je hebt bij de <public health service> curssen ''Omgaan met scheiding'', enzo. Mijn ervaring is dat mensen daar niet echt op zitten te wachten, omdat die problemen daar al zijn en ze denken dan dat ze daar hun vuile was moeten buitenzetten.
***Zou je dan voorstellen om ouders daar heen te laten gaan?***Ja, hoe gaan we daar nou op een goede manier een plannetje van maken.

Algemeen:
***Zijn er nog dingen die echt niet ter sprake zijn gekomen?***
Nou kijk, wat wij, ik heb naar aanleiding van de pilot die we hier hebben gedaan met het JGT het met een JGT-collega over gehad: Ik denk omdat bij ons de POH-GGZ redelijk functioneert blijft het toch wel de meest logische plek waar het contact plaats vindt. Wij zien mensen 20 of 30 minuten, maximaal in twee-drie keer. Ik heb dan wel snel het gevoel: nu moeten we toch wel richting gaan krijgen en als dat nog te weinig is dan moet je naar de POH-GGZ. Vaak is de JGT ook niet zo: 'je hebt een ingegroeide teennagel, ga naar het JGT', vaak is het een ingewikkeldere context waarvan je zegt: 'nou het JGT is aangwezen' (blijkt ook wel dat je een aantal stappen hebt). Dus komt dat bij ons wat minder vaak voor omdat ze al bij de POH-GGZ, die heeft ook ... met school en wordt er ook duidelijk gezegd: 'Oh hier speelt dit, hier speelt dat.' Ik denk dat dat wel een rol speelt waarom wij zeggen: 'Oh ja, dat zou wel een goede zijn om nu met het JGT te overleggen'' en daarbij komt ook nog dat: Ik had laatst iemand dat ging ook niet goed op school, er was pestgedrag en dan denk ik ook wel 's van: 'Is het JGT hier nou bij betrokken, of niet? En dan weten mensen zelf ook niet zo goed of ze nou wel of niet zijn betrokken. Dan denk ik ook wel 's van: 'Ik kan niet tegen mensen zeggen, ik ga het met het JGT bespreken. Nee, daar moeten ze eerst toestemming voor geven. En als zij dan zeggen: 'Ik weet niet wat het is.' Daar zit ook wel 's een lastig punt. Daarom is het heel belangrijk dat je weet dat het JGT is betrokken. 'Oh ik weet dat het JGT betrokken is, vind je het goed als ik overleg met hen?' Dat lijkt mij een ideale entree.
***Als ik het goed begrijp noem je daar dus een verbeterpunt of in ieder geval een punt waar winst in is te behalen. Zijn er nog andere punten waarvan je zegt :'Nou, dat kan beter'?*** ***Bijvoorbeeld in het contact met gespecialiseerde zorg?***Oh, <naam specialistische GGZ-instelling> enzo?
***Ja.***
Nou, normale problematiek. Lange wachttijd, maar ik vind dat <naam specialistische GGZ-instelling> heel laagdrempelig benaderbaar is voor overleg. Als je wil kan je altijd overleggen en word je vriendelijk bejegend. Alleen, als je iemand wilt insturen is dat vaak een heel lang traject. Maar, dat weet je. Dat is gewoon zo. Nee, op zich vind ik dat wel prima.

EINDE INTERVIEW

**Interview Htrb1**Algemeen:
***Naam?***
<Naam huisarts>.

***Hoe lang bent u al huisarts?***Vanaf '94. Dus 22 jaar.

***En hoe lang al in deze praktijk?***
21 jaar. Één jaar ben ik waarnemend huisarts geweest.

***Alvast toespitsend op het onderwerp waar we het zo over gaan hebben: hoe vaak komt het nou voor dat u gezinnen danwel kinderen/jongeren binnen de context van een gezin ziet met psychosociale problematiek?***Ja, dat vind ik heel lastig. Ik denk dat je het niet zo labelt, dus het is echt de film afdraaien om te kijken hoe vaak dat voorkomt. Ééns in de twee weken? Het is een gokje, hoor. Als je vraagt: gaat er dan altijd een verwijzing uit? Nee.

***En is er hier een POH-GGZ waarmee u samenwerkt?***Ja.
***En ziet zij ook kinderen en jongeren?***Jongeren wel, maar het is niet haar specifieke aandachtsveld. Ik zou wel een ouder met een kind kunnen verwijzen om voorbereidend werk te doen en om het probleem wat beter in kaart te brengen, maar het is geen POH-GGZ jeugd.

***En hoe vaak komt het nou voor dat u met hem of haar om de tafel gaat zitten om dit soort problematiek te bespreken?***
Speciaal jeugd? Niet. Na de koffie hebben we altijd overleg, de autorisaties doornemen van de assistentes en daar zit zij ook vaak bij en dan blijft ze nog even hangen om één of twee casussen door te spreken. Maar als ik dan terugdenk van: hoe vaak is dat jeugd? Nee, niks. Kijk, de meest gesteld vraag is toch: heeft mijn kind ADHD? En die moet je eigenlijk gelijk verwijzen, daar zit weinig tussenweg. En als het echt problematiek is die het hele gezin betreft, dan verwijs je zelf naar het JGT, als het eenvoudige problematiek is dan verwijs ik ze eerst naar het Centrum voor Jeugd en Gezin (soort opvoedvragen). Of je dat dan labelt als psychosociale problematiek, dat vind ik een beetje lastig. En JGT kan ook psychiatrische problematiek in zitten, ergens daar zit wel het scheidsvlak maar ik vind dat heel moeilijk om die lijn te trekken: wat is psychosociaal en wat is psychiatrie - dat is ook mijn expertise niet.
***En kan de POH-GGZ daar nog een aanvullende rol in spelen, met betrekking tot dat onderscheid?***Nou, je hebt het over jeugd. Met volwassenen kan ze heel goed uit de voeten. Jeugd is toch een aparte tak van sport en dan denk ik: het JGT en het CJG dat is al eerste lijn - zo wil <naam regio> dat apart gepositioneerd hebben, dus dat is als ik het naar de POH-GGZ zou verwijzen dan is dat meer van hetzelfde. Nee, dan passeer ik echt de POH-GGZ, zo is zij niet bedoeld, zij is meer voor de volwassene. Ze zou het wel willen, hoor. We hebben de mini-anamnese over ADHD, wat <naam huisarts> in <naam plaats> heeft ontwikkeld. Dat is een richtlijn, zeg maar, als ouders komen met de vraag: heeft mijn kind ADHD? Dan schijnt dat 2/3e inderdaad zwaar genoeg is om doorverwezen te worden en in behandeling komt, maar 1/3e loopt eigenlijk niet vast maar is er wel die vraag. En men zegt dat de POH-GGZ met een begeleid vragenlijstje dat zelf zou kunnen doen. Ze heeft haar commitment gegeven dat ze dat gaat doen, maar het is eigenlijk niet voorgevallen. En mijn ervaring is dat mensen hier toch zitten met de vraag: ja, heeft mijn kind het nou? Ik vraag me af of de POH-GGZ dan voldoende gewicht in de schaal legt om dat te kunnen zeggen: ''ja, je kind heeft het'' of ''nee, het heeft het niet.'' Dus dan zit je toch aan een verwijzing vast. Dus eigenlijk dat lijstje, daar komt in de praktijk niet veel van terecht. Ouders poneren zo duidelijk die vraag: heeft mijn kind ADHD? Nou dan moet je naar een instelling die dat test en dan krijgen ze een ''ja'' of een ''nee''. Meestal is dat natuurlijk een ja, maar goed. Dus daar voegt de POH-GGZ weinig aan toe, ja.

***Nou we komen er zometeen ook zeker over te spreken, ik heb er ook wat aanvullende vragen over.***
Maar dat is ook geen psychosociaal probleem he, dat is een psychiatrische diagnose van: ''heeft mijn kind dat wel of niet?''
***Dus daar zit het onderscheid ook?***Ja.
***Oké, dat wat betreft de algemene vragen. Dan gaan we nu over op de casussen.***

Casus 1 (Dave):
1. Autisme of ADHD. Ja, en wat wil je van mij weten?
***De eerste vraag die ik altijd stel is: er is natuurlijk weinig informatie gegeven maar wat is nu de eerste indruk die u krijgt?***Geen, nou ja ik ga vragen stellen. Wil je weten wat voor vragen?
***Ja (haha).***Ik wil weten of 'ie vast loopt op school, gaan zijn cijfers achteruit. Hoe oud is 'ie? 6 jaar? Nou ja, komen de vaardigheden op gang of lijkt dat al te stagneren? Hij zit al in groep 4 waarschijnlijk. Wordt moeder gek van hem? Leidt het tot confrontaties? Nare situaties? Is er nog een kind dat misschien minder aandacht krijgt omdat Dave negatieve aandacht naar zich toetrekt? Dat soort dingen. Heeft Dave vriendjes? Hoe zit hij sociaal in zijn vel? Dat zijn eigenlijk ook vragen die op dat ADHD-lijstje van <naam huisarts> voorkomen. Op drie gebieden: school, thuis, sociaal. Sport, zit 'ie op sport? Hoe gaat dat? Als hij verliest, gaat hij dan door het lint? Kan hij tegen zijn verlies? Dus zo probeert ik een inschatting te maken. En kijken wat de vraag is van moeder. School denkt aan ADHD, ja wat denkt moeder? Heeft moeder een vraag?
***Ja, dus echt de hulpvraag verduidelijken?***
Ja.
***Is dit een herkenbare casus?***Dit is wel een veel voorkomende vraag.
***Lees gerust het tweede gedeelte ook alvast door.***2. Moeder komt met vader en dan komt dit verhaal.
***Ja dus er wordt nu wat meer duidelijk over de situatie, hoe het nou precies met Dave gaat. Zijn er nou al elementen uit dit verhaal waarvan u zegt: dat doet mijn beleid het meeste vormen?***Ja, ik wil nog wel eventjes weten hoe ouders op hem reageren. Zit het moeder tot hier, omdat ze er slecht mee om kan gaan? Of zit het vader tot hier, tot wat voor gedrag leidt het bij ouders en inderdaad bij broertjes of zusjes want dat zou richting aangeven richting het JGT, maar als de vraag is: ''wat mankeert Dave?'' - dan gaat 'ie dus richting de GGZ, specialistiche GGZ want er zit een duidelijke ADHD-vraag bij. Ik heb net een hele discussie met JGT erover gehad, ik had weer geprobeerd om een lichte ADHD-situatie bij het JGT onder te brengen - ze willen het niet hebben. Ze kunnen het niet, dus hup specialistische GGZ.
***Dus weer de hulpvraag die ...***...ja, want ze willen weten is het ADHD, zit het in de familie? Dat lijkt wel die kant op te gaan, ook het verhaal doet me er wel aan denken. Nou, dan moet er een ja of een nee uitkomen en dan daarna ga je met begeleiding aan de slag bij wie dan ook (?).
***Ja, want we hebben het een deel over JGT gehad, lees de rest van de casus ook maar even door.***Ja, dus de school heeft JGT er al bij gehaald?
***Ja. Want u gaf net al aan dat er een gesprek was met het JGT, wat dan toevallig net is gebeurd. Hoe gaat die samenwerking voor de rest? Is ook een belangrijk onderdeel van onze hoofdvraag.***Ja, wat zal ik daar 's van zeggen? Ik zie dat ze heel erg hun best doen om contact te leggen met de huisartsen, we hebben een bijeenkomst waarin het hele JGT er zat en waarin er voorgesteld werd. Ja, en dan is de belangstelling vanuit de huisartsen toch heel erg laag.
***Hoe komt dat, denkt u?***Tijdgebrek, andere zaken. Ja, je moet je spreekuur vrijplannen, dat komt er allemaal bij. Ja en het is nu de tweede keer dat er wat onduidelijkheid is of ik nou naar hun kan verwijzen (en dat is dus twee keer dat ik naar hun probeer te verwijzen, maar dat dat dan niet kan). En dat vind ik jammer, zij proberen zich te positioneren en ik heb steeds begrepen van ''ja dan verwijzen wij ook door.'' Want wij als huisartsen mogen eigenlijk niet meer verwijzen, dat wil de wethouder. Nou, dan probeer ik het weer naar het JGT. Maar die zegt: ''nee hoor, zeker bij ADHD: no way!''
***Ja, dus als ik het goed begrijp: als huisarts wordt het niet helemaal duidelijk van waar...***... Ja, we willen best wel doorverwijzen naar het JGT, maar dan moeten ze het ook oppakken en als dat niet hun piece of cake is, dan mogen ze het wat mij betreft doorverwijzen naar hun instelling - want er zitten mensen van <naam specialistische GGZ-instelling>. Ja, wat voegt het dan toe als ik dat dan doe? Tuurlijk, ik vind het fijn, dan schrijf ik het verhaal en dan krijg ik terugkoppeling en van het JGT is het een black box. Dat zou een argument zijn dat ik het doe, maar dat is niet hun argument. Hun argument is: ''we hebben die expertise niet in huis.''
***Iets wat ik veel zie in andere interviews is de vraag van: in hoeverre is de specifieke huisarts bekend met het JGT? Hoe zit dat bijvoorbeeld hier in <naam gemeente>?*** ***Hoe hebben zij zich op de kaart gezet?***De coördinator is alle huisartsen langs geweest om kennis te maken. Dat is op zich een prima manier, want dan is het gewoon twee afspraken, 20 minuten en dan kun je dus heel gericht praten. Maar misschien n.a.v. mijn vraag -dat weet ik niet meer- dat ik inderdaad van de expertise wil weten. Zijn we een keer daar geweest, daar zat het hele rondje, maar dat gaat ook het ene oor in en het andere oor uit. Dat beklijft nog niet zo erg. Dus ze doen wel veel om zich bekend te maken, maar het jammere is dat ADHD het meeste voorkomt en dat doen ze juist niet. Er zit gewoon een scheef ding. Iedereen kan naar het JGT verwijzen, net als in deze casus: heeft school al die rol opgepakt. Dus dan is het een beetje zoeken naar je plaats: wat kan ik dan nog toevoegen en wat moet noodzakelijkerwijs door mij uitgevoerd worden? Nou zo'n verwijzing van mij naar de s-GGZ, maar ja het ligt natuurlijk ook voor de hand dat als ze bij het JGT zijn dat die JGT die stap neemt.
***En welke rol speelt de huisarts idealiter: school kan bijv. al doorverwijzen naar het JGT, maar welke rol zou de huisarts daar nou idealiter in spelen in dat hele proces?***Nou ja, we zijn natuurlijk wel gezinsarts - dus dat komt ook iedere keer ter sprake, dat wij graag verslaglegging willen vanuit het JGT wat er gebeurt. Als je Pietje hebt bijv., dat er een melding staat dat hij bij het JGT zit. Als je dan moeder spreekt en je zit toevallig dan even bij de kinderen te kijken, dat je kan zeggen: ''Goh, Pietje, JGT; hoe gaat dat nou thuis?'' Dat je dan langs je neus zegt dat je uitvoering kan geven aan je taak als gezinsarts? Nou dat gebeurt dus niet, in het gunstige geval krijgen we een berichtje terug dat iemand zich aangemeld heeft bij het JGT maar we krijgen geen medische informatie. Dus het ondermijnt onze taak als gezinsarts enorm, dat vind ik echt zonde en ik denk dat dat niet de wens van de patiënt is. Die vindt het prima als de huisarts de informatie verzamelt.
***Ja, want ik kan me ook zo voorstellen dat de patiënt ook terugkomt en dan verwacht van de huisarts dat die de informatie heeft.***Ja, over het algemeen verwacht de patiënt dat wel. In dit geval.. je stuurt ze eigenlijk... het is een black box. Ze proberen zelf, maar dat lukt nog niet altijd, als we echt iemand actief verwijzen, ook een briefje mee te geven zodat duidelijk is dat 'ie verwezen is en ik probeer zelf twee maanden later eens een telefoontje te plegen om actief de vinger aan de pols te houden. Maar dat is bijna geen doen, wij zijn responsief in de zin. We acteren alleen als er een vraag is. Pro-actief, zo zit ons systeem niet in elkaar, dus dat vraag van ons weer een omwenteling en als dat dan via dat systeem van het JGT niet ondersteunt wordt, dan denk ik ook van: nou, laat dan maar zitten. Daar word ik moedeloos van. Het moet in het systeem passen en als iemand naar het JGT is verwezen vanuit school, dan weet ik daar ook niets van.
***Oké, interessante punten in ieder geval. We hebben het nu over het JGT, maar komt het ook voor dat u doorverwijst naar vrijgevestigde hulpverleners in de buurt?***Ja.
***En welke zijn dat dan zoal?***Er is een kinderpsychiatriekliniek (poli) die ADHD test. Dat vooral. Er is een kinderpsycholoog waar we ook wel eens naar verwijzen. Ja, en daar komen dan de brieven van terug dus dat is op zich heel prettig.
***Ja want wat is voor de rest als u die samenwerking nou tussen u en die vrijgevestigde hulpverlener nou vergelijkt met de samenwerking tussen u en het JGT: wat zijn de grootste verschillen?***Dat is de terugkoppeling en dat als je belt, dan weet je dat die persoon gezien is door diegene die je aan de lijn hebt. En bij het JGT gaat dat over verschillende schijven, dus dan moet je aan die patiënt vragen: goh, met wie spreek je bij het JGT? Oké, nou dan ga je bellen naar het JGT: goh, ik wil even overleggen met die en die - met toestemming van de patiënt -. Nou, dan ben je een week verder totdat je die persoon gesproken hebt. Met een vrijgevestigde is dat één op één, dus dat werkt veel makkelijker. Het moet allemaal snel-snel. Er zijn korte lijntjes (met die vrijgevestigde hulpverlener).
***Zou dat dan helpen als u als huisarts ook rechtstreeks iemand kent vanuit het JGT? Iemand die diezelfde korte lijntjes kan bieden, bijvoorbeeld?***
Ja, ze zijn bezig met - misschien heb ik die hier wel liggen - een lijstje met 06-nummers maken. Ze willen ontzettend graag hoor. Ik weet niet of ik die hier al heb, maar er ging een lijstjerond met e-mailadressen en 06-nummers. Ze willen op zich wel.
***Er zit wel beweging in, dus?***Ja, er zit wel beweging in. Maar goed, de insteek blijft anders. De insteek blijft dat de ouder en het kind dossierhouder zijn en dat iedereen daar naar toe kan verwijzen, dus die huisarts heeft die centrale plek niet meer. Die moet bevochten worden, zeg maar (als je dat al wilt), of je zegt: ''het systeem verandert, oké. Ik hoor het wel als ze me nodig hebben.'' dan krijg je meer zo'n houding.
***Dus enerzijds ben je dan ofwel ketenspeler, ofwel spin in het web?***Ja en die spin in het web ben je dan dus niet meer, je bent evt. ketenspeler. Maar ook dan, ketenzorg (GGZ) gaat ook altijd met verwijsbrieven en terugverwijsinformatie, dus in die zin heeft het JGT dat heel anders vormgegeven met een ander uitgangspunt.

***Interessante punten, in ieder geval. Gaan we naar casus 2, alweer***.

Casus 2 (Sanne):
1. 2. en 3. tezamen.
***Hele andere casus, natuurlijk. Wat is volgens u nou het kernverschil, kernwoorden ten op zichte van de eerste casus? Wat is nou anders?***Ja, de vraag hier van de ouders is: ''wat is er aan de hand met Dave?'' en wat is de vraag hier? Hier zie ik geen vraag. Hier komt niet een hulpvraag uit, dus de vraag is hoe jij op de hoogte komt van de situatie. Want als moeder komt met Sanne, dan zal er ook een hulpvraag zijn.
***Die wordt alleen niet al te duidelijk geponeerd?***Nee, die wordt niet duidelijk. Het is echt niet duidelijk wat moeders vraag is. Of heeft Sanne een vraag? Of heeft school een vraag? Ze wil graag niet naar school? Is ze door school gestuurd omdat ze teveel verzuim heeft?
***Ja dus vanuit verschillende partijen kan de hulpvraag komen? Of meerdere partijen?***Ja.
***Hoe zou u dit aanpakken?***Haha, ja dat hangt van de hulpvraag af.
***Wat zijn de dingen die u verder wilt weten om het probleem verder te verduidelijken?***
Ik zou misschien vooral met Sanne willen praten. ''Omdat school zich niet houdt aan de afspraken die zijn gemaakt.'' Ja, is er ooit een diagnose bij Sanne gesteld of rommelt ze maar zo door? Een benedengemiddelde intelligentie, driftbuien. Nou ja, inderdaad, de casus heet ''veiligheid'' dus een kindje van 1.5 als die slachtoffer wordt van de driftbuien van een 14-jarige, dan kan dat pittig uitpakken. Dus daar zou ik wat over willen weten van moeder. En die moeder is nu boos. Wat doet moeder in haar boosheid en op wie is ze nog meer boos? Benedengemiddelde intelligentie: is er ooit een intelligentietest gedaan? Want als dat niet het geval is dan zal het de moeite waard zijn omdat inderdaad te laten testen, want dan kan ze extra begeleiding krijgen. Dit is wel iets voor het JGT, denk ik, omdat uit te zoeken. Dit is wel, er zitten verschillende factoren: aan welk touwtje ga je trekken?
***Dus omdat het een soort multiprobleem is, zou het bij het JGT thuis horen?***
Ja, klein huis, financiële zorgen. Ook het toekomstperspectief, hè, wat voor kant gaat Sanne op? En weet je, het rare van die casus is dat zo'n gezin allang ergens in de picture is. Dus het is bedacht voor het papier, maar zo'n casus is allang ergens op de basisschool ontspoord en die hebben een on-off relatie met het JGT waarschijnlijk of met...
***<Naam specialistische GGZ-instelling>?***... ja, precies. Maar dat weet je natuurlijk niet. Anders zou ik haar inderdaad naar het JGT verwijzen om eens te kijken van welke factoren er nou meespelen en hoe het hele gezin ondersteund kan worden.
***Dus ook voor ouders en evt. voor broertjes en zusjes?***Ja, vijf kinderen. En als je dan driftbuien moet managen met van die kleine kinderen eromheen.
***Er staat nog een belangrijke zin: dat ouders niet altijd op één lijn zitten qua opvoeding. Stel het komt nou voor dat vader het helemaal niet eens is dat ze naar het JGT wordt dooverwezen en moeder is daar bijvoorbeeld groot voorstander van. Hoe gaat u daar nou mee om?***Ik zou vragen wat vader zijn aarzeling is, want vaak kun je die kou uit de lucht halen als je zegt: het kan er alleen maar beter op worden, maar misschien slaat hij deze of genen wel en is hij bang dat dat daar naar boven komt. En wil hij dat niet, dan snap ik dat wel. En dat is ook, begreep ik van die bijeenkomst met JGT, iets waarover gedacht wordt. Je komt er voor hulp, maar ook het gedwongen kader hangt er aan vast. Dus ja, dat kan wel een aarzeling zijn. Als je dat weet, als dat eenmaal bekend is: toen hadden we zorg en dat ging niet goed, toen ineens kreeg ik een ondertoezichtstelling onder m'n neus.
***... dat kan allemaal meespelen ...***... Ja, als vader dat weet dan denkt 'ie van: nou maar even niet. Maar, hoe je dat uiteindelijk vlot trekt? Kijk, moeder heeft natuurlijk een zelfbeslissingsrecht in die zin, dat ze eerst zelf er heen gaat en de situatie bespreekt. Het is niet charmant, maar het kan allemaal wel.
***Hoe pakt u dat bijvoorbeeld aan: stel, moeder en vader komen dan met Sanne. Is het dan ook zo dat u het consult in delen knipt. Dat u zegt van: ''moeder, blijf nog maar even in de wachtkamer wachten.'' of, hoe gaat zoiets?***Nou, als ze het eerste consult met z'n drieeën een afspraak hebben, dan zitten ze hier met z'n drieeën. Dan is het een beetje heen en weer praten, een beetje sfeer proeven. En als er dan een vraag bij mij komt: ze laat het achterste van haar tong niet zien, dan probeer ik Sanne alleen een keer te krijgen. Van: ''joh, kom je samen met moeder of vader maar dan wil ik je wel een keertje alleen hier hebben en dat moeder in de wachtkamer blijft, of zo.'' Ja, en op zich hebben ouders niet echt een probleem mee. Ze voelen zich niet bedreigd ofzo, heb ik het idee. Op zich is dat, zeker als je zegt vanaf 12 jaar is dat redelijk gebruikelijk dat kinderen zelf komen. Dat zijn toch wel kleine dingen die heel vertrouwelijk zijn.
***Ja en misschien dat zo'n eerste gezamenlijk consult ook gebruikt kan worden om vertrouwen te bouwen om vervolgens afzonderlijk af te spreken.***Ja, en ze hebben die afspraak gemaakt dus dan voeg ik me daar in en daarna heb je gerede argumenten om te zeggen: ''moeder, wil je nog een keer komen om het verhaal van jouw kant te belichten, of ...?''
***Duidelijke en interessante punten, wederom. Gaan we alweer naar de laatste casus.***

Casus 3 (Melany):
1.2. en 3. tezamen

Melany komt met beide ouders, maar dan gaat ze dit niet vertellen - kan ik je vertellen. Dit is echt een verhaal wat ze vertelt, zonder ouders.
***Dat heb ik vaker gehoord, hoor. Dat de volgorde van consulten...***... ja.
***Eigenlijk dezelfde vraag als bij casus 2: waarin verschilt in uw opinie deze casus t.o.v. die andere casus?***
De vraag is of vader en moeder een probleem hebben, of alleen Melany een probleem heeft. Kijk als gecommuniceerd is door school dat ze Melany te los laten, is een beetje de vraag hoe ze daar op hebben gereageerd: hebben ze dat ter harte genomen en willen ze daar iets mee maar ontbreekt het ze aan de vaardigheden? Dan zou je dat kunnen ondersteunen. Maar anders, als je vader en moeder nog steeds zo ziet, dan zit Melany er tussen in en dan moet Melany ondersteund worden, vooral.
***En ondersteuning in welke vorm?***Het zal op zich wel bij het JGT ondergebracht kunnen worden. Want ze hebben wel iemand die speciaal voor jongeren/adolescenten goed is, dus afgeleide (?) kinderen in de puberteit. Ze is natuurlijk 15, dus je moet wel bijsturen wil ze straks een beetje goed de volwassenheid ingaan. Ik zou dan voor het JGT kiezen, omdat dan toch ook de ouders erbij gehaald worden. Kijk, een vrijgevestigde kinderpsycholoog, daar is net teveel gezinsproblematiek voor. Die kan natuurlijk wel een goede vertrouwenspersoon zijn voor Melany, want ik denk dat zij wel een vertrouwenspersoon nodig heeft en die niet noodzakelijkerwijs met haar ouders gaat praten. Dus echt voor haar. Die leeftijd, met dit soort puberteitsproblematiek maar ook somberheid, denk ik van - ik gun haar een vertrouwenspersoon. En of dat dan binnen het JGT is of...
***Dat is inderdaad mijn volgende vraag...***Nou het JGT heeft meerdere disciplines in huis, dus je zou ouders voor opvoedondersteuning kunnen aanmelden, maar een apart iemand voor Melany. Misschien zou ik even overleggen van: joh, dit is de situatie. Ik ben niet zoveel van het overleggen want dat kost ook weer enorm veel tijd, in die zin, maar als je denkt van: ik wil wel dat ze goed terecht komt.
***Dan eventueel een tweesporenbeleid, dat de kinderpsycholoog ook mee kijkt?***Ja.
***En, speelt hier ergens nog de POH-GGZ een rol?***Oh oké! Dat zou inderdaad nog kunnen. Dat is een andere combinatie dan, of je stuurt het hele gezin naar het JGT of je zegt van: nou, sowieso zou de POH-GGZ in eerste instantie wel de ouders kunnen spreken of moeder en Melany kunnen spreken. En inventariseren wat er allemaal speelt en checken of ze niet te somber is, zeg maar, of je het niet een depressie moet noemen. Ik kan me voorstellen dat het te pittig is voor de POH-GGZ, maar dan heeft ze wel twee of drie gesprekken gehad met een goede inventarisatie en dan weet je ook beter waar de behandelruimte zit. Als vader en moeder wel een probleem hebben maar die willen niet, dan houdt het ook op.
***En hoe zat dat overigens met casus 2? Kwam daar POH-GGZ nog in uw gedachtengang voor?***Nou, wat ik zeg: bij die jeugd zit het niet echt in m'n hoofd, dan zit het JGT in mijn hoofd.
***Ik geloof dat ik zo de belangrijkste dingen de revue wel heb laten passeren. Ik weet niet of u nog dingen wilde aansnijden?***Nou het verschilt ook wel hè, want dit is een meisje (casus 2) dat lage intelligentie heeft, dus dat is toch wel weer andere problematiek. Dit lijkt meer, onder andere, op gewone puberteitsproblematiek die wat uitvergroot is door allerlei redenen. Dus hier zou de POH-GGZ wel wat meer mee kunnen dan met het meisje uit casus 2, hoewel ze allebei ongeveer 15 zijn. Dat is een aparte tak van sport, lage intelligentie. Ik schat in dat met dit grietje wel te praten valt, maar dat zal moeten blijken.

Algemeen:
***We zijn aan het einde gekomen van de casus - uitleg artikel en streven naar aanbevelingspunten-. Wat zijn volgens u nou aanbevelingspunten die daar echt in moeten komen?***Want je wilt het JGT beter positioneren?
***Ja we richten ons in de eerste plaats op de samenwerking met het JGT en in de tweede plaats ook op de specialistische GGZ***.
Ja, sowieso een lijst van: dit zijn de medewerkers van het JGT; wat is hun expertise, hoe kunnen we die bereiken? Welke dagen werken ze? Onder welk telefoonnummer zijn ze dan te bereiken? Dus dat ik gericht een advies kan vragen, maar als er een week over heen gaat voordat ik de juiste persoon te pakken heb, dat vind ik lastig. Dat demotiveert me. Dat, ik zou eigenlijk willen dat er andersom geredeneerd wordt: er gaat een bericht naar de huisarts, tenzij... maar dat zit lastig, dat is ook een ICT-probleem want zij hebben een heel ander systeem. Maar goed, er moet toch een briefje uit gedestilleerd kunnen worden dat vanuit <beveiligd correspondentiesysteem> onze kant op kan komen. Dat zijn de belangrijkste wensen.
***Dat zijn punten die niet mogen missen in dat lijstje?***Ja. En ik vind dat eigenlijk dat ze, dan zeg ik iets wat tegen mijn eigen belang in gaat: Ik vind eigenlijk dat ze gewoon geen nee mogen verkopen. Ze zijn gewoon geen goede handelaren, hè: terugverwijzen van ''doet u de verwijzing maar''. Dat vind ik zo slap. Maar ja, ze doen het wel. Dus, laat ze dat dan maar anders aanpakken, dat zij gewoon dat kind verwijzen naar de s-GGZ. In eerste instantie toen het systeem opgezet werd, was het idee dat alleen de JGT zou mogen verwijzen naar de s-GGZ. Nou, door een lobby van de huisartsen is er in gekomen dat wij toch ook blijvend mogen verwijzen naar de s-GGZ. En nou, dit is de omgekeerde wereld: dat ze terugverwijzen omdat wij moeten verwijzen naar de s-GGZ. Dat voelt-niet-goed! Laat ze dan zeggen van: oké, nou ja, je had het ook zelf kunnen doen - maar ik verwijs wel. En dan is via nascholing of via een nieuwsbrief daar duidelijkheid over geven. En gooi mijn eigen glazen in, want ik realiseer me: als ik zelf verwijs naar de s-GGZ, dan krijg ik een net briefje terug van wat er gebeurt is. En bij hun niet, dan is het een black box. Maar ik wil best kosten besparen en ik denk dat door naar het JGT te verwijzen dat ik kosten bespaar. En dat zij dan nog een zeef hebben van: moet dit wel verwezen worden? Maar ja, dat wordt niet bewaarheid.
***Goed punt, inderdaad. Dus dat toch ergens iets in die keten niet helemaal loopt zoals het bedoeld was om te lopen?***Ja.

EINDE INTERVIEW

**Interview Hp2fi**Algemeen:
***Hoe lang bent u al huisarts?***Een jaar of 10. Ja, 10 jaar.
***En hoe lang werkt u al hier, in deze praktijk?***10 jaar. En het is nu sinds 8 jaar mijn praktijk.
***En voor mijn begrip: van wie was het voor die tijd?***
<Naam huisarts>, dat was mijn voorganger.
***Alvast een beetje toespitsend op het onderwerp waar we het natuurlijk zometeen over gaan hebben: hoe vaak komt het nou voor dat u psychosociale problematiek op het spreekuur ziet (binnen gezinnen)?***
Van kinderen of volwassenen?
***Van kinderen en jongeren***.
Minimaal wekelijks, soms dagelijks.
***Redelijk frequent dus.***Ja.
***En wat voor soort problematiek moet ik dan aan denken?***Dat kan zijn opvoedingsproblemen, dat je ziet dat mensen heel vaak ziek zijn en daardoor heel vaak van school zijn waarbij ik dan denk: goh, die ouders houden die kinderen veel te vaak thuis, maar ook ouders waarbij de kinderen zo vaak ziek zijn dat op een gegeven moment denkt van: nou, dit is gewoon niet meer normaal terwijl er nooit wat onder ligt. Dat je denkt van: dit is meer een probleem van de ouder dan van het kind. Maar ook dingen als: anorexia, relatieprobleem van de ouders, schoolverzuim, heel soms drugs (dat kom je hier niet zo veel tegen); alcohol dat kom ik relatief vaak tegen - er wordt veel gedronken op het platteland, onzekerheid, ADHD kom ik relatief veel tegen (dat vind ik altijd een lastig probleem); nou dat zijn zo'n beetje de dingen waar je veel tegen aan loopt.
***Ja. U noemde het al een beetje, op het platteland wordt veel gedronken. In welke optiek verschilt de problematiek naar uw mening die u hier ziet t.o.v. een huisarts in de stad?***
Ik weet niet of de huisartsen in de stad het minder hebben, daar heb ik gewoon geen ervaring mee, maar ik zie dat de jeugd hier heel veel drinkt. Ik denk dat het ook gewoon verveling is, van die zuiphokken waar ze gaan indrinken en ik weet niet of het ergens anders beter is. Maar het is hier wel een probleem, dat jongeren vanaf 16 al beginnen te drinken - ook al mag het dan eigenlijk niet - maar ik heb ook echt wel 's meegemaakt dat ik tegen een jongen zei van: ''je moet echt minder gaan drinken want je lever gaat er van kapot.'' ''Ja, ja, ja, dat is dan jammer.'' Dat z'n moeder erbij zat en zei van: ''Ja, ik kan hem toch ook niet thuis houden.''
***Ik kan me voorstellen dat dat als huisarts zijnde even achter de oren krabben is.***Dat is moeilijk, ja.
***Hebben jullie hier voor de rest een POH-GGZ met wie jullie ...***Ja.
***En komt het vaak voor dat kinderen of jongeren...***
Jongeren eigenlijk niet, dat zou wel kunnen want ze heeft wel ervaring met jongeren. Maar ik stuur kinderen meestal naar het Centrum Jeugd en Gezin. Ik vind dat wel een heel prettige instantie omdat ze ook dingen door kunnen pakken, door kunnen sturen en omdat het vaak een complexer probleem is. Dat het niet alleen de kinderen zijn, maar bijna altijd systeemproblemen zijn met de ouders er bij. Als het met één kind niet lekker loopt dan lopen er vaak meerdere kinderen niet lekker. Dus ik vind een POH-GGZ, dat kan je bij een heel duidelijk alleenstaand probleem wel doen maar mijn ervaring is dat het bijna nooit alleen het kind is. En dan vind ik het lastig om een POH-GGZ daarvoor in te schakelen, ik denk dat het Centrum voor Jeugd en Gezin beter is. En als het om kleine dingetjes gaat: slaapproblemen, eetproblemen. Op jonge leeftijd ervaren ouders dat als grote problemen maar als huisarts vind ik het niet hele grote problemen, dat hoort gewoon bij Centrum Jeugd en Gezin.
***... omdat het Centrum voor Jeugd en Gezin het systeem ook in acht neemt?***Ja, het is altijd een interactie tussen ouders en kinderen.
***Ja, en naar welk Centrum voor Jeugd en Gezin verwijst u het meeste naar toe?***Dat van <naam plaats>. Eigenlijk alleen naar <naam plaats>, want mijn hele populatie is <naam gemeente>. <Naam plaats>, <naam plaats>, <naam plaats> is allemaal <naam gemeente>.
***Ik stel nu heel wat vragen die oorspronkelijk eigenlijk later in het interview naar voren komen maar we hebben het er nu toch over: u noemt dat het prettig is om met het CJG samen te werken, wat spreekt u vooral zo aan in in het contact?***Vooral het feit dat ze alles doen, dat ze één zorgverlener per gezin doen, dat ze van kleine zorgjes tot grote zorgen kunnen oppaken. En ook als er meer nodig is dat ze de wegen weten waar ze heen moeten sturen voor de echte grote problematiek.
***Het is toch een relatief nieuw initatief, natuurlijk.***
Ja, ze zijn vanuit <naam gemeente> vroeg begonnen en ze hebben ook heel goed op de kaart gezet: in de zin van de artsen goed geïnformeerd en duidelijk hebben gemaakt wat de voordelen zijn. Dus ik denk dat ze het hier goed hebben aangepakt.
***Want, kunt u enkele initatieven noemen die zij hebben ondernomen om het...***... nou, niet eens initiatieven - zou ik niet weten - maar gewoon van het feit dat ze vanaf het begin naar ons toe hebben gecommuniceerd van: dit gaat er komen, dit gaan we starten en dit kunnen we. Dat is gewoon heel prettig, en vooral ook: wat er ook is, stuur maar naar ons. En dat is gewoon heel handig, want we hebben zoveel instanties waar we heen kunnen sturen dat het gewoon heel prettig is dat er gewoon één instantie is waarvan je weet: daar stuur ik ze heen en dan komt het wel goed en anders hoor ik het wel van.
***Ja. Dus een soort spin in het web?***Ja.
***En hoe gaat voor de rest het contact in de zin van de terugkoppeling die jullie ontvangen?***Dat kan beter. Ik vind dat er weinig terugkoppeling komt, ik vind het ook moeilijk om ze te bereiken dus daar ben ik niet supertevreden over. Ik heb het gevoel dat als mensen er eenmaal bij zitten dat het wel loopt, maar het erbij komen wil nog wel 's moeizaam gaan: dat mensen lang moeten wachten, dat ze niemand bereiken, dat ze niet teruggebeld worden. Mijn ervaring is ook dat het lang duurt voordat ik contact heb, dus daar ben ik wat minder tevreden over.
***Hoe zou dat komen, denkt u?***Ja, weet ik niet. Weet ik echt niet.
***Interessante punten in ieder geval. Een goede start van het interview (haha). Dan stel ik voor om aan de casuïstiek te beginnnen***.

Casus 1:
1. Wat is je vraag?
***Wat is de eerste indruk die u krijgt als u dit zo leest?***Ja, mijn eerste indruk is van: hoe is moeder? Is moeder ongestructureerd en is het ''tutedetuut'' of is het een hele reëele moeder? Ik wil eerst weten hoe moeder is want heel vaak moeders die roepen dat hun kind ADHD heeft, dat zijn vaak moeders die - mijn ervaring is - dat het moeder zijn die het zelf niet aan kunnen en die heel weinig structuur kunnen bieden. Ik denk dat ik maar eerst 's even ga kijken hoe moeder in elkaar zit.
***En hoe zou u dat aanpakken?***Ja, meestal weet je het wel een beetje. Je hebt die ouders al een aantal keer gezien met snotneuzen, oorontsteking, keelontsteking en dat soort dingen. En je kent moeder ook als patiënt want de ouders zitten over het algemeen ook bij mij. Meestal heb ik wel een idee van hoe moeder is en als ik moeder niet ken dan is het in ieder geval geen onzekere of pieperige moeder en dan zal ik eerder denken van: wat gaan we hier verder mee doen?
***Oké. En wat denkt u dan bijvoorbeeld van school, want ze komt op advies van school?***Dat vind ik wel een punt, al moet ik zeggen: school willen ook nog wel 's heel makkelijk roepen dat iets ADHD is, hoor. Dat neem ik met een korreltje zout, maar ik vind wel dat als alleen moeder het zegt en op school gaat het prima. Dan.. het zal wel meevallen als school het ook zegt.. ik neem het wat serieuzer als mensen - zowel ouders als school - zeggen dat het ADHD is. Als alleen ouders het zeggen of alleen school dan zeg ik van: goh, ga nog 's met elkaar om de tafel waarom kind thuis het gedrag wel vertoont en niet op school en omgekeerd. En als ze het allebei zeggen: ja dat is... ADHD, trouwens, dat is iets wat ik vaker niet naar het Centrum voor Jeugd en Gezin stuur, dat stuur ik naar de GGZ want ik vind dat ADHD vaak een supermoeilijke diagnose is - juist omdat het zo vaag is en zo een (?)curve met een afkappunt: ''daar wel en daar niet.'' Dat kan gewoon niet, eigenlijk vind ik het een rotdiagnose dus ik doe behandeling van ADHD echt niet zelf bij kinderen, tenzij ze al uit de puberteit zijn en al een vaste hoeveelheid medicatie gebruiken want ik vind dat dat helemaal niet bij de huisarts hoort. Ik denk dat er dat er echt een overbehandeling van ADHD is en misschien zelfs een onderbehandeling van degenen die - nou dat was vroeger meer, denk ik - dus die zou ik echt naar de GGZ sturen.
***Dat is inderdaad een interessant punt, onze onderzoeksvraag richt zich op én JGT's én op specialistische zorg. Eigenlijk dezelfde vraag als die ik bij de JGT's stelde: hoe ervaart u die samenwerking?***
Ik vond hem heel goed totdat de bezuinigingen ingezet werden en ik vind hem nu minder goed. De wachttijden zijn bizar lang, de onderzoeken worden onnauwkeurig uitgevoerd, ik heb regelmatig dat het enige onderzoek zegt: ''het is ADHD'' en dan klopt er iets niet, of ouders zijn het er niet mee eens, of het is geen ADHD en dan stuur ik het naar een andere instantie en dan komt er een andere diagnose uit. Dus ik ben de laatste jaren echt heel ontevereden met de GGZ, daar gaat het heel ongelukkig mee. Heel jammer.
***Ja, vervelend, inderdaad. Wat zouden mogelijkheden kunnen zijn om dat te verbeteren?***
Ik denk dat de GGZ hier niets aan kan doen, ik denk dat de GGZ is wegbezuinigd en dat alle goede mensen die expertise hebben opgebouwd, die zijn weg of die zijn ontslagen, die zijn doorgegaan naar een andere functie waar ze ... Dus dat betekent dat expertise weg is, ik denk dat dat 10 jaar duurt voordat dat weer terug is. Ik denk dat dat voorlopig niet meer goed komt. Misschien dat Centrum voor Jeugd en Gezin daar een stukje in over kan nemen, maar als het echt gaat om de echte psychiatrie denk ik dat er de komende tijd geen oplossing voor komt. Daar ben ik heel negatief over.
***Want u heeft het over lange wachttijd en de vraag over terugkoppeling: hoe gaat dat dan bijvoorbeeld?***Terugkoppeling gaat prima, dus als ze iets doen.. het vervolg wordt wel goed teruggekoppeld, maar ik vind vaak dat kinderen snel worden teruggestuurd met weinig hulpmiddelen en veel medicatie en weinig andere handvaten. Dat is eigenlijk heel jammer.
***Dat is iets wat u vervelend vindt?***Ja, zeker.

***Stel ik voor dat we alweer een consult verder gaan. Lees het gerust door.***

2. en 3.
Oké.
***Doet dit uw idee van het probleem of van een mogelijke oorzaak van het probleem veranderen?***Eigenlijk stuur ik ADHD, als mensen er echt heel volhardend in zijn - als ik uitleg dat het vaak een kwestie is van structuur aanbrengen - en dan heb je zo'n mooi filmpje van thuisarts.nl met een video erbij; als ze dan blijven volharden van: ''het gaat niet, het is ADHD.'' dan stuur ik ze eigenlijk altijd naar de GGZ. Dus dat zou ik hier ook doen.
***Geen verandering dus?***Nee.
***Ja en dat stukje van de kinderneuroloog, zou dat nog uw gedachten doen veranderen?***Ja, ik denk dan eerder aan een beperking, bijvoorbeeld. Misschien heef 'ie qua ontwikkeling een achterstand en daar kun je ook bij overvraging dit soort klachten krijgen maar dan heb ik zoiets van: dat moet of de kinderneuroloog of de GGZ uitzoeken, daar kan ik niet zoveel mee. Dat gaat mijn pet te boven.
***Dan stel ik voor dat we weer een consult verder gaan. In deze casus is dan nog niet besloten om door te verwijzen naar de specialistische zorg. Denkt u dat het JGT hier nog wat mee zou kunnen?***Dat denk ik niet, ik denk dat dit zo'n zwaar geval is: ik zou 'm naar de GGZ sturen. Zijn er veel huisartsen die dat wel met Jeugd en Gezin doen?
***Misschien is dat goed om dat aan het einde van het interview te vertellen om ''bias'' te voorkomen (haha).***Ja, precies.

Casus 2:
1. en 2.
Ja, nou als ik dit zo lees dan denk ik van: moeder zal waarschijnlijk ook benedengemiddelde intelligentie zijn - dat zijn vaak families, het is een beetje erfelijk. Ehm, last van uitbarstingen heeft vaak ook met opvoeding te maken dus dit is bij voorbaat iets wat ik zou sturen naar Centrum Jeugd en Gezin.
***Moeder speelt ook een rol in deze.. heeft u de rest overigens ook al gelezen?***Nee.. *leest*. Heel herkenbare casus.
***Ehm, moeder speelt een belangrijke rol in deze casus. Zou u nog zorg voor haar willen regelen? Of ondersteuning voor haar willen bieden?***Dat zou ik eigenlijk ook naar Jeugd en Gezin willen.. die geven ook begeleiding van hoe je met kinderen om moet gaan en hoe je de opvoeding op je neemt. Maar dat komt ook vanuit Jeugd en Gezin zelf, dus als moeder zou zeggen: ik kan het niet meer aan en niet persé zou komen met Sanne, zou ik haar ook naar Centrum voor Jeugd en Gezin sturen. Ik zou hierbij als moeder, als moeder het allemaal teveel wordt, en CJG niet genoeg is zou ik ook nog aanbieden dat ze naar de POH-GGZ kan, voor begeleiding hoe ze zelf met de situatie om kan gaan.
***Oké, lees gerust nr. 3 ook alvast door.***

3. Oké, probleem.
***Meer een algemene vraag wat betreft deze casus: kunt u een kernwoord of kernwoorden noemen waarvan u zegt: daarin verschilt deze casus ten opzichte van die eerste? Wat uw beleid doet veranderen?***Dit is veel meer een interactieprobleem. Casus 1 komt veel meer vanuit dat jochie en casus 2 - als Sanne andere ouders had met veel meer bagage - zou het helemaal geen probleem zijn met Sanne. Dus hier is echt begeleiding nodig, terwijl bij dat andere kind echt gekeken moet worden wat hij persoonlijk nodig heeft. En hierbij moet echt gekeken worden: wat moet er in dat gezin gebeuren om dat gezin stabiel te houden en om Sanne te begeleiden? En daar moet je ouders voor begeleiden om dat op te pakken.
***En als ik het goed begrijp: dit is dus meer een systeemprobleem dus kan het beter naar CJG?***
Ja, precies.
***Oké, dan gaan we alweer naar casus 3***. ***Weer een andere situatie.***

Casus 3:
1. 2. en 3.
Zelfs die naam (haha), typisch.. niet deze naam maar alles wat eindigt op van die ''Y-en'', dat is vaak laag economische status.
***Haha, herkenbaar.***
Schuurfeest, ja hoor.
***Ik moest ook al denken aan deze casus toen u het zojuist vertelde.***
Dit is een heel herkenbare casus, eigenlijk. En wat is je vraag?
***Ja, eigenlijk hetzelfde als bij die vorige casus. Waarin verschilt deze casus t.o.v. die andere twee?***Dit is late problematiek, dit is een kind dat zich aan het losmaken is in de puberteit. En, dan kun je wel met het systeem veel doen - en dat moet ook -. Maar het is veel lastiger omdat ze zich juist probeert te onttrekken aan het systeem, dus dan moet je ook het systeem leren dat ze aan de ene kant regels moeten stellen en aan de andere kant haar op eigen benen moeten leren laten gaan. Ik zou hier ook Centrum voor Jeugd en Gezin weer invoeren, maar alleen mijn ervaring is dat mensen dat niet willen want dat vinden ze voor kleinere kinderen en wat doen ze nou helemaal? Dus dit is veel moeilijker. En, Melany heeft natuurlijk hier ook zelf veel meer bij in te brengen, want bij die vorige casus: dat is in principe een meisje dat qua leeftijd in de puberteit zit maar wat veel meer nog een kindje is. Dit is echt een vrouw die onderweg is naar de volwassenheid, dus die gaat niet zomaar akkoord met naar Centrum voor Jeugd en Gezin gaan en de vorige die komt wel mee. Dus dit is wel een lastige.  ***Zou je ook kunnen stellen dat, juist vanwege haar leeftijd, dat ze een grotere rol speelt in haar eigen problematiek?***Ja zeker, maar dat zal ze ook nemen. Die laat zich niet zomaar van alles opleggen.
***Oke, nou deze casus gaat het er ook over - ouders zijn gescheiden dus die komen over het algemeen niet overeen qua gedachtegang.***Dat is ook nog een probleem, als de vader wel behandeling wil en moeder niet - of omgekeerd. Dat heb ik meegemaakt, heel moeilijk.
***Wat doet u daar mee?***Ja, niks. Dan meld ik bij vader dat ik het er niet mee eens ben en dan houdt het op. Het is heel vervelend. En als ik denk dat het de spuigaten uitloopt, dan meld ik het bij <naam advies- en meldpunt voor huiselijk geweld en kindermishandeling>. Maar dat heb ik best wel vaak gedaan, maar die kunnen ook niet zoveel doen. Die willen ook in principe dat Centrum voor Jeugd en Gezin aan de gang gaat, maar die kunnen dan bijvoorbeeld met vader praten van: waarom doe je dat niet?
***Is dat iets wat je dan ook in de spreekkamer al noemt t.o.v. vader? Stel vader blijft op de rem trappen.***Wat het probleem vaak is dat vader na zo'n echtscheiding dan al ergens anders zit. Dus dan zit 'ie bij een andere dokter en dan komt 'ie niet op het spreekuur. Mijn ervaring is dat het dan heel lastig is om vader te bereiken. Als 'ie op het spreekuur komt, dan is het probleem er niet. Dan zeg je van: goh, ik vind dat u niet goed bezig bent, ik vind het een soort kindermishandeling als u uw kind hier niet heen laat gaan want het gaat niet goed met 'r. Dan gaan ouders wel 's overstag, maar als vader dan in een hele andere praktijk zit, dan is het moeilijker - ik zou het op zich wel benoemen, maar niet over de telefoon.

***U had nog een vraag die ik aan het einde van het interview kon beantwoorden, wat was 'm ook alweer?***Dat ging over mijn collega's, casus 1, of ze die naar GGZ sturen of naar Centrum voor Jeugd en Gezin.
***Ja, dat wisselt heel erg. Afhankelijk van of ze ook een POH-GGZ in de praktijk hebben en ja de ene huisarts heeft ook wat meer affiniteit maar ook wat meer kennis over de JGT - juist omdat het ook een relatief nieuw iets is - dus ja, dat wisselt heel erg maar dat is ook wel heel erg leuk om te zien om per interview die antwoorden in kaart te brengen en zo vult de puzzel zich in.***
Ja.
***Ik kom dus natuurlijk aan het einde van dit onderzoek tot een wetenschappelijk artikel en we hopen dit ook internationaal te publiceren - althans, dat is wel de insteek. Daarbij hoop ik ook tot een aantal aanbevelingen te komen, bijvoorbeeld voor de praktijk van de huisarts maar ook bijvoorbeeld voor Centrum voor Jeugd en Gezin en de specialistische zorg. Zijn er nou nog punten waarvan u zegt: ''die moeten er echt in of die kunnen niet missen?''***Het is een beetje een klein punt, maar wat ik net zei over dat ADHD. Ik weet niet of je daar iets mee kan, hoor, maar ik vind het echt onjuist dat ADHD - want dat heeft nu een standaard voor de huisarts, dat betekent dat wat we in principe moeten gaan doen; en ik ben het er echt niet mee eens, ik vind dat dat zo'n beladen diagnose is die zoveel consequenties voor de toekomst heeft; en ik zou het er mee eens zijn op het moment dat wij als huisarts voldoende begeleiding zouden kunnen geven om gezinnnen te structureren. Maar dat hebben we niet. Dus, de diagnose stellen en het vervolgens niet kunnen begeleiden betekent in feite dat je al die kinderen veroordeelt tot medicijnen en dat vind ik persoonlijk echt niet goed. Ik zou het heel fijn vinden als er een instantie is, als het CJG zou kunnen - dat zou geweldig zijn-, dat er echt goed naar wordt gekeken naar dat ze in eerste instantie vooral kijken naar begeleiding in structuur en kijken of je die ADHD of ADHD-trekken dat je die op een niet-medicamenteuze manier kunt begeleiden en als dat echt niet gaat dat je dan bijvoorbeeld een psychiater in consult neemt of desnoods via de huisartsen doet als je er goede protocollen voor hebt maar dat je echt alles geprobeerd hebt. Nu is het echt vragen om al die kinderen medicatie te geven en dat vind ik echt zonde.
***Ja, want je hoort ook wel 's dat ADHD een soort ''hypediagnose'' is. Is dat iets wat je beaamt?***Het is niet zozeer een hypediagnose is, maar ik denk dat het komt door veranderingen in de maatschappij. We hadden vroeger veel minder multitasking en kinderen en volwassenen moeten nu van alle kanten multitasken. En ik denk dat je, als je dat doet, dat je dan meer in een ontwikkeling richting ADHD gaat. En als je op dat moment ouders hebt die aan alle kanten van alles moeten doen en daardoor en daardoor ook hun eigen structuur kwijt zijn, dan denk ik dat je ook die kinderen niet meer die structuur kunt bieden dus ik denk dat er een oorzaak is voor die ADHD-toename en ik denk niet dat dat komt door kleurstoffen of genetische afwijkingen in de populatie of... ik denk dat het een cultureel iets is. Maar ik denk wel dat het bestaat en ik denk ook, doordat het een cultureel iets is, dat je het ook moet kunnen oplossen. Maar goed, daar is natuurlijk weinig onderozek naar dus dit is puur mijn natte vinger gokwerk.
***Je zou bijna zeggen dat de oplossing dan in de politiek zou zitten.***
Ja, maar die politici die leven ook zo dus ik denk niet dat je de maatschappij die de ADHD veroorzaakt dat je dat kunt oplossen, maar ik denk wel dat je de kinderen die daar gevoelig voor zijn - dat je die door heel goed te structureren en te begeleiden op het rechte pad kan houden. Want vroeger had je rust, reinheid, regelmaat - nou, hartstikke ouderwets: alleen al het zinnetje, daar krijg je kotsneigingen van. Maar ik denk wel dat die kinderen die richting de ADHD gaan, dat die er heel veel baat bij hebben. Maar dat doen ouders niet meer, dat kan niet meer. Je hebt van alle kanten heb je internet open staan, je hebt telefoons die aan alle kanten piepen. En ik doe er net zo hard aan mee.
***Ja, leerzaam. Interessant wat we zo dit half uur hebben besproken. Nogmaals, ik ga hier een wetenschappelijk artikel van maken en de deelnemende huisartsen die krijgen daar ook een seintje van. Wat ik ook altijd doe, en dat heb ik ook beloofd per mail: is een klein presentje. Misschien leuk voor in de spreekkamer of misschien voor thuis. Kijk, staat een beetje symbool voor de richting in de zorg en in de jeugdzorg. Nou, het kan zo zijn dat je binnenkort een mailtje ontvangt met de telefonische vragenlijst. Nou, die ga ik opstellen aan de hand van deze interviews - ga ik een aantal gemeenschappelijke vragen opstellen en dan hoop ik ook de betrouwbaarheid van de gegevens die ik heb verkregen door de interviews in de regio te kunnen verstevigen. Dat gebeurt uiteraard allemaal anoniem, natuurlijk. Daar mag je nog steeds aan meedoen, je hebt natuurlijk nu ook al hele waardevolle bijdrage geleverd.***Nou, dank je wel.
***Dat was het wat mij betreft. Zijn er nog dingen onbesproken gebleven?***Nee hoor.
***Dan ga ik 'm (de geluidsopname) even stopzetten.***

EINDE INTERVIEW

**Interview H0eum**Algemeen:
***Hoe lang bent u al huisarts?***Ik ben vanaf eind '85 huisarts, dat is alweer 31 jaar - iets langer, denk ik.
***En hoe lang werkt u al in deze praktijk?***31 jaar.
***Alvast toespitsend op het onderwerp waar we het zo over gaan hebben: hoe vaak komt het nou per week, per maand voor dat u op uw spreekuur gezinnen met psychosociale problematiek ziet?***
Nou, dat kan ik niet gelijk voor de geest houden. Psychosociale problemen, dat is iets van 2-3 per maand - zo ongeveer. Ik denk dat het meer is dan een oorontsteking en dat er dan dus wat anders achter zit. Het is niet zo veel. Ik denk 2 per maand, ja. Misschien wel 1 per maand. Het kan zijn dat mijn collega op vrijdag er ook nog eentje uitpikt.
***Wat ziet u dan, bijvoorbeeld?***Het kan zijn problemen op school. Het kan zijn, vorige week had ik iemand: een zus komt hier binnen met trauma's - omdat haar broer op haar ingeslagen heeft, dat schiet me te binnen. Andere zaken kan zijn via school: mogelijke incest bij een meisje, dat heb ik twee maanden geleden gehad. Daarvoor pestgedrag door een kind op school. Nja, dan stopt het eigenlijk wel. Dus het is niet zo veel: het is misschien wel eens in de twee maanden is dat je echt zo'n psychosociaal probleem hebt. Tenminste, dat je er echt in moet duiken en moet nagaan met de begeleiders op school: oh ja, nou moeder komt hier met kind - altijd keelpijn, moeder wordt er gek van, kind naar de KNO-arts; daar komt niets uit. Nou, dan vraag je hoe het op school. Dan hoor je op school dat er sprake is van agressie, nou dat is dan 4.
***Het komt niet zoveel voor, maar dus wel redelijk gevarieerde problematiek?***
Nee, het komt niet supervaak voor. Meestal worden psychosociale dingen door school opgepikt of door andere instanties.
***Werkt hier voor de rest een POH-GGZ waarmee u samenwerkt?***
Ja, er komen soms kinderen (middelbare scholieren, meer) met identiteitsproblemen, depressies, en dan blijkt vaak ook dat het psychosociaal is, dat - laten we zeggen - het kind wordt geplaagd door broers; oftewel vernederd of gepest en schoolresultaten vallen tegen, willen stoppen met de studie (gymnasium of MAVO), dus dat komt ook voor.
***En welke rol neemt de POH-GGZ hier in? Is dat met name diagnostisch, of is dat ook een stuk behandeling? Hoe gaat dat?***Dat is meer begeleiding.
***Dat waren de algemene vragen.***

Casus 1 (Dave):
1. ***Wat is de eerste indruk die u krijgt, als u dit zo leest?***Het kind kan moeilijk met frustraties omgaan, dus dan zou je eigenlijk moeten vragen: wat is hier nu aan de hand? Zit/is (?) de thuissituatie van veel frustratie en problemen, kwaad als het niet gaat zoals hij wil: is hij driftig? Zit het in de familie? Had vader of moeder dat ook, vroeger? Autisme of ADHD: komt dat in de familie voor, kan. Is het een jongen die altijd wel heel kieskeurig was, of het kind was altijd heel kieskeurig met eten bijvoorbeeld. Of heeft hij specifieke hobby's: alleen maar autootjes verzamelen of poppen. ADHD, ja het zijn wel tegenstrijdige dingen, maar het komt wel 's gezamenlijk voor. Dus ja, wat je in zo'n situatie zou doen is.. ik denk niet dat ik het gelijk aan de POH-GGZ zou sturen. Ik zou aan de hand van de informatie die ik krijg zou ik denken: nou ja, misschien toch inderdaad een kinderpsycholoog zou ik naar toe sturen met: speelt dat nu, is dat nou ADHD of is dat autisme? En er komt wel vaker naar voren of het wel of niet is en ik heb één of twee kinderpsychologen waar ik wel 's naar toe stuur.
***En welke zijn, dat voor mijn begrip?***<Naam vrijgevestigde kinderpsycholoog, daar stuur ik wel 's naar toe en er is er nog ééntje, maar ik kan niet op de naam komen. <Naam vrijgevestigde kinderpsycholoog> op de <naam straat>, dat zijn denk ik de twee dingen waar ik naar toe zou kunnen sturen. Als het echt autisme is, waarvan ik denk: er is echt sprake van autisme, dan is het toch <naam specialistische GGZ-instelling>.
***In <naam plaats>?***
Ja. ADHD wordt <naam specialistische GGZ-instelling>, of <naam specialistische GGZ-instelling> - dat zijn twee grote organisaties. Of, als je in <naam plaats> woont is het bij de kinderpoli.
***Deze vraag: autisme al dan niet ADHD, is dat iets wat u vaak te horen krijgt qua hulpvraag waar ouders mee komen?***ADHD wel, want daar ergeren ouders zich nog wel 's aan. Autisme komt niet zo vaak voor, omdat stel dat ouders het zelf hebben - dan herkennen ze het zelf niet eens. Dan weten ze niet en dan vinden ze het ook moeilijk om te accepteren. Autisme, daar is minder bekendheid over.
***Stel ik voor dat we een stapje verder gaan***.

2. Ja, je zou denken: dit jong gehoorzaamt niet. Die moet gewoon gestraft worden en de vraag daarna komt eigenlijk van mensen die vroeger zo'n gedrag hadden, vaak toch wel behoorlijk in het gareel moesten worden gehouden. Die vader zegt dan: ja, aan z'n oren mankeert niets, wat betekent dat als zo'n man dat noemt? Heeft die vader, behalve dan dat 'ie misschien aan z'n oren trekt. Je moet 's luisteren, speelt daar misschien nog wat meer mee: in de opvoeding? Is vader bijvoorbeeld handtastelijk of geeft hij klap of iets dergelijks? Dat zou nog wel kunnen, dat die Dave moeilijk in het gehoorzame pad te krijgen is. Nou goed, aan de andere kant denk ik -als ik dit zo lees-: zou het kind nog slimmer kunnen zijn dan dat mensen denken? En je moeten afvragen: heeft hij inderdaad ADHD en speelt er misschien Asperger een rol - dat hij toch slimmer is dan wij denken? En dat hij ondergestimuleerd wordt op school, dat ze dat helemaal niet zien: gewoon een lastige jongen. Bedplassen en dergelijke, dat kan wel bij deze leeftijd passen dus dat is niet zo bijzonder. Daar zou je nog een plaswekker voor kunnen geven of zo'n soort advies. Nou, dat is een beetje wat er aan te doen is op dit moment. Ja, kinderpsycholoog is nog niet benoemd, zie ik al, wel de kinderneuroloog. Ja, dat kan geen kwaad om dat na te laten kijken maar toevallen, uit het niets wit worden en overgeven - ja, voor hetzelfde geld denken we: ja, heeft hij uberhaupt wel iets gegeten? Is hij gewoon niet te moe? Geeft hij over omdat hij te moe is, dat het kind wegtrekt omdat hij eigenlijk over zijn grenzen gaat in zijn activiteiten? Ja, dat zou ik zo maar een beetje bekijken.
***Ja, want u noemde ook de vraag welke rol vader speelt in deze problematiek: misschien handtastelijkheid, en dergelijke. Hoe zou u die informatie boven tafel willen krijgen? Ze zitten dan voor u, moeder en vader: is dat dan iets wat u alleen met moeder bespreekt of meteen met beiden?***Nee, moeder komt nu met vader dus dan kan je zeggen: nou, als ik ze hoor is het zo dat hij eigenlijk niet (?) aan tafel, maar ja: vroeger, de opa's en oma's; dus de ouders van de mensen die voor me zitten, dan werd je erbij gehaald en dan kreeg je een draai om je oren. Maar is hij ook zo dat hij behoorlijk bij de les gehouden moet worden met bijvoorbeeld - dat je zegt - ik moet hem aan z'n oren trekken of een klap om z'n oren geven, of iets anders. Dat hij dat nodig heeft. Dan komt er wel uit: nou ja, dat was in onze tijd zo maar dat doen we nu met ons gezin niet, zoiets dergelijks. Of: ''Dokter, van ja, nee hoor zo ver gaan we niet.'' Maar ik moet die vraag wel stellen, want zo is het soms ook inderdaad zo. Sommige mensen schamen zich ervoor, maar je moet er wel naar vragen. Niet als verwijt, maar meer van: nou ja, ik begrijp dat u erg uw best doet om hem bij de les te krijgen. Het is wel uw zoon en je hebt wel het beste met hem voor.
***Duidelijk verhaal, inderdaad. Dan stel ik voor dat we naar punt drie gaan. Daarna is de casus ook afgelopen.***

3. De situatie is onhoudbaar geworden. Nou ja, goed, daar kan ik niet zoveel aan doen - deze situatie. Dan denk ik: er zijn vragenlijsten afgenomen op school, en thuis over aandachtsproblemen: autistisch of sociaal probleemgedrag. Ja, dan is een beetje de vraag: ze komt later nu wel met Dave, dus Dave is nu mee. Dus dan zou je kunnen zeggen: ''beste Dave, ik hoor het verhaal van je moeder. Er zijn allemaal testen afgenomen.'' - dat zou voor mij de eerste vraag aan moeder zijn: ''Wat wilt u op dit moment?'' Ik hoor dat er een heleboel mensen inmiddels aan zijn gaan trekken: Jeugd en Gezin, orthopedagoog. Nou ja, dan denk ik bij mezelf: hoe vindt moeder dat het zelf loopt? Heeft zij vertrouwen in het JGT, dat ze bij mij komt? Moet ik met hun overleggen? Ja, wat vindt Dave er zelf van, van: waarom is hij onhoudbaar geworden? Wat kan hij er zelf van vertellen. Misschien zegt hij wel van dat hij gewoon gepest wordt omdat hij allerlei opmerkingen plaatst die te slim zijn - noem maar op. Ja, heeft hij de neiging om terug te pesten - kan ook nog een rol spelen. Verder kom ik ook eigenlijk niet.
***We zien hier al het woordje ''JGT'' staan, is dat iets waar u met regelmaat in uw eigen praktijk mee samenwerkt?***
Weinig, ik moet zeggen sinds de verandering van het gebeuren (?), ik denk dat het misschien 4x per jaar is - dat ik overleg, dat ik moet bellen of dat er een overleg is.
***En hoe ervaart u die samenwerking, als 'ie er is?***Het punt is dat je niet weet waar wie per regio zit, dus dat is elke keer weer opzoeken. Via internet kom je erachter dat daar Jeugd- en Gezinsteam zit en dat je daar die moet bellen. Op een gegeven moment weet je het van een bepaalde regio wel iets beter, maar scholen houden zich niet aan regio's dus sowieso ligt dat ook weer anders. Maar mensen wonen of verhuizen ook weer anders. Ik ben meer een familie-huisarts dan een wijkhuisarts, dus dat is misschien ook wel een nadeel in dit opzicht: hoewel ik me dat afvraag.
***Voor mijn begrip: waar zit het verschil met name in?***Met families heb je de hele familie, heb je dan: opa, oma - de hele familie ken je. Terwijl, als wijkhuisarts dan ben je gewoon voor een tijdje de huisarts van een gezin, dan verhuizen ze buiten die wijk en dan komt er weer een ander gezin voor in de plaats. Dus je hebt wat meer long-life vervolg en dan krijg je dus ook weer dat je denkt: ''oke, die lijkt op die oom en dat ging toen zo en zo.''
***De context heeft u in kaart?***Ja. En het Jeugd- en Gezinsteam, ja die orthopedagoog - misschien dat mijn collega's dat wel beter weten - maar die orthopedagogen wisselen ook per zoveel tijd. Dus dan is bij Jeugd- en Gezinsteam de vraag van: hoe continu is dat nou? Daar weet ik te weinig van.
***Als ik het goed begrijp: u werkt met name dan samen met de kinderpsychologe waar u het zojuist over had?***Ja. Al die jaren heb ik meer houvast gekregen aan kinderpsychologen. Je weet wie ze zijn, ze komen langs, als je ze belt heb je een kort lijntje, het blijft stabiel - ook al is het 10 jaar -, Jeugd- en Gezinsteam, ik moet zeggen: ik zou het adres niet eens weten en ik zou het gezicht niet eens weten. Het is dat er een Jeugd- en Gezinsteam is, bijv. in <naam stad> op het <naam plein>: daar zit een Jeugd- en Gezinsteam, waar nog steeds een consultatiebureau zit. Nou, dat is het enige waar ik dan van weet.
***Dus met name de onbekendheid met het Jeugd- Gezinsteam dat maakt...***... ja.

Casus 2 (Sanne):
1. 2. en 3. tezamen.

Ja, bij Sanne. Tenminste, als ik het eerste stukje lees. Woede-uitbarstingen, tenminste: ze is de oudste, komt er misschien toch teveel druk op dat kind. Omdat ze een voorbeeld moet zijn of omdat ze verantwoordelijkheden krijgt en dan is de vraag of ze niet op haar tenen loopt: 14 jaar, in de puberteit, dus daar kunnen een heleboel dingen een rol spelen. Dus ja, deze stuur ik dus ook naar een kinderpsycholoog of naar een psycholoog die bij <naam specialistische GGZ-instelling> gewerkt heeft omdat haar intelligentie wat lager ligt - om te kijken hoe ze om kan gaan met die driftbuien maar ook om in kaart te brengen van: nou, speelt daar geen autisme in? Maar goed, dat komt meer in de volgende vragen naar voren, als moeder later met Sanne terugkomt: van nou daar komen toch die rituelen erg naar voren. De vraag is ook: ze heeft last van haar knieeën. Is ze ondertussen veel te zwaar geworden, dat ze eetbuien heeft of heeft ze eetbuiten passend bij een meisje die in de groei zit? Ze wordt ook gepest dus dat speelt natuurlijk ook wel een grote rol, daar moet met school overlegd worden. Dus dat koppel ik dan vaak terug naar moeder: heb het er met school over want pesten (1 op de 10 kinderen wordt gepest, dus dan heb je er één te pakken), maar dan maak ik er een punt van (?): heb het er over met school hoe dat dan zit. De vraag is ook een beetje: ze heeft een vast ochtendritueel, vaste tijden, past dat bij autisme of past dat gewoon bij een soort dwangmatig karakter wat zich gaat ontwikkelen en komt dat bij familieleden voor?
***Dat zou u willen vragen?***Ja, dat zou ik willen vragen. Nou dan komt moeder later met Sanne, aan het einde. Ja, dan is de vraag ook een beetje: moeder heeft heel veel lichamelijke klachten waarbij ze Sanne in moet schakelen. In welke mate is er ruimte dat moeder als het ware daar nog aan gaat werken, evt. bij de fysiotherapeut of bij een psychosomatische fysiotherapeut om er mee om te gaan? Want ze noemt dan zelf osteopenie, maar dat zal het niet wezen - het zal waarschijnlijk meer fibromyalgie wezen, waarbij ze deze klachten heeft. Pijn in de handen, dat kan artrose zijn. Dat kind vindt het verder wel leuk om thuis te werken, alleen de vraag is: hoe vinden die anderen dat? Dat zusje, want dat oudste zusje moet dan zorgen voor de jongste en voor de andere kinderen. Is dat niet al wat parentificatie, is dat niet al van dat je een kind teveel verantwoordelijkheden geeft - waardoor het niet aan de eigen ontwikkeling toe komt? Nou, daarin moet je natuurlijk ook gaan denken: dan is ze natuurlijk niet zo intelligent. Maar daarin kan ze te vroeg de vader-moederrol overnemen, waardoor het kind niet toe komt aan haar eigen ontwikkeling en zelfs daarbij ook gepest wordt. Dus dan krijg je dat je een soort inhaalslag krijgt. Nou, daar zou mijn vrouw dus wel wat mee kunnen als POH-GGZ, om met die moeder eens te praten om dat meer plek te geven. Moeder zal waarschijnlijk ook wel wat minder intelligent kunnen zijn, maar dan nog zal mijn vrouw een paar goede gesprekken met die moeder kunnen hebben want die moet volgens mij ook wel inzien dat het ergens niet goed gaat. Nou, dat zijn zo'n beetje de dingen.. dus moeder een gesprek bij de POH aanbieden, moeder een gesprek met school aanbieden i.v.m. pesten en dan evt. Sanne zelf nog een gesprek hebben met een kinderpsycholoog en die dan wat ervaring heeft met lage intelligentie.
***U beantwoordt al impliciet een boel vragen die ik had willen stellen, dus dat is heel goed. Ik had nog wel twee overige vragen hierover. U zegt een aantal interessante dingen, maar wat is nou volgens u het voornaamste verschil wat betreft deze casus t.o.v. de eerste casus? Wat is het kernwoord, denkt u?***Één van de kernwoorden zou de intelligentie kunnen zijn en het andere verschil is ofwel: Sanne is erg gestructureerd en die andere heeft geen structuur. Dat zijn de twee belangrijkste dingen.
***En is het voor de rest een herkenbare casus, dit?***Ja, dit komt wel vaker voor, ja.
***U heeft al een aantal opties genoemd wat betreft beleid, maar ik begrijp dat u ook zorg voor ouders - naast Sanne en misschien voor broertjes en zusjes - zou willen arangeren?***Nou, het begint bij moeder. Moeder is verantwoordelijkheid voor de veiligheid van die kinderen - staat er zo mooi boven. En dan: kan moeder het überhaupt wel aan, vijf kinderen? Want vijf kinderen, ja hoe gaat het met de andere kinderen? Hoe gaat dat, eigenlijk?
***U snijdt een aantal interessante punten aan. Ik stel voor dat we naar de volgende casus gaan.***
Casus 3:
1. 2. en 3.

Dat is natuurlijk een kind wat inderdaad knel zat, door een scheiding. Al wel begonnen toen ze 8 jaar was, is ze bij moeder gezeten - denk ik -, om en om zitten ze dan.
***Ja klopt.***
Ja, dus vanaf het 8e jaar. Dat kind heeft natuurlijk allerlei ruzies meegemaakt toen ouders bij elkaar waren: vader met boze buien en zoals ik het lees, deze casus, dan zouden er een paar dingen kunnen gebeuren; dat ik of moeder met de POH-GGZ laat praten, of Melany. En ik denk, dat zou allebei los kunnen, want het kind is losgeslagen maar het lijkt erop alsof ze geen stuur meer heeft in zichzelf. Ze zit natuurlijk ook nog in de puberteit, moet ook nog haar identiteit zien terug te vinden en zoekt dit dan vooral terug in het contact met jongeren - maar dit hoort bij de leeftijd. Ze heeft nergens een motivatie voor, maar in principe als je ziet dat ze goed kan leren en goede cijfers haalt, ja dan heeft ze wel het koppie om te leren maar ze heeft geen enkele motivatie ervoor. Onrechtvaardigheid is echt een punt waar ze boos van wordt, ze kan hard zijn maar dat heeft te maken heben met de gevolgen van de scheiding die ze in haar jeugd heeft meegemaakt. Dan slaat ze ook nog door dat ze veel drinkt in de schuur (nou ja, alcoholisme), dan is de volgende vraag: van alcohol, en dan vrijen; hoe doen we dat met anticonceptie? Want de geschiedenis herhaalt zich dan vaak weer, dat ze dan in verwachting raakt en abortus krijgt en dan zegt: ''zie je wel, ik deug helemaal niet; ik kan er helemaal niets van.'' En dat kan nog steeds een reactie zijn op die scheiding, op: ''Vonden ouders mij dan niet de moeite waard om bij elkaar te blijven?'' - bijvoorbeeld. Wat is er toen gebeurd? Heeft daar Jeugd- en Gezin bij gezeten, bijvoorbeeld, of <naam advies- en meldpunt voor huiselijk geweld en kindermishandeling>? Maar ja, in eerste instantie denk ik gewoon: je kan daar een inventarisatie van doen, maar ik denk dat het gesprek van Melany met de POH-GGZ zou wat meer rust kunnen geven. En de POH-GGZ is contextueel opgeleid, waarbij ze eigenlijk meer aandacht geeft aan: wat is de betekenis geweest van die scheiding en ook zoals Melany er nu in zit? Nou, mocht dat niet lukken dan kom je wel uit op een aantal andere instanties, zoals die kinderpsycholoog of soms - als ze heel erg gaan drinken, inderdaad - een schooldecaan of een schoolverpleegkundige; daar heb ik ook wel 's wat contacten mee gehad, dat ze elke week een gesprek had met een verpleegkundige op school. Dat zou ook nog kunnen. Nou ja, Melany is 15 jaar dus ze heeft nog een eigen verantwoordelijkheid in dit gebeuren - ook wettelijk - dus die moet er wel in mee betrokken worden. Ik zit zelf te denken aan: Melany een keer gesprek hebben (Melany die komt met beide ouders), dus je zou kunnen uitleggen dat communicatie tussen de drie heel moeizaam loopt en dat we zorg hebben over Melany, maar dat Melany er eerst met zichzelf moet uit zien te komen en dat Melany een gesprek met mijn POH-GGZ kan hebben.
***Zoals u kunt lezen is er een scheiding geweest, moeder en vader kunnen nogal niet overeen komen qua ideeën. Wat doet u nou als vader zegt: ''nou er is helemaal geen probleem!'' of die vindt dat er een ander beleid nodig is dan moeder, bijvoorbeeld. Hoe gaat u daar mee om in uw spreekkamer?***
Ja, Melany is 15 jaar dus in zoverre heeft zij zeggenschap in het hele gebeuren. Dus tenzij er echt grote dingen zijn waarbij het kind levensgevaar loopt, zoals niet insturen bij blinde darmontsteking of levensgevaarlijk gedrag, dan heb je als ouders relatief niet zoveel zeggenschap. Maar dat zou ik even uit moeten zoeken hoe ver dat dan ligt. Stel, die vader zegt: ik vind dat mijn kind opgenomen moet worden in een internaat of - hoe zal ik het zeggen - een pleeggezin, bijvoorbeeld; want ik vertrouw mijn vrouw niet. Ja, dan kom je toch bij kinderbeschermingsinstanties uit, zeker als het kind heel vaak drinkt in de schuur en dronken is. Ja, dat is wel een item waarvan je denkt: ''ja, dat kan niet.'' Dan beschadig je d'r zodanig dat je eigenlijk moet denken van: ''dit gaat niet goed.'' dus daar moet wel een andere instantie bij. Dat zal ik uit moeten zoeken, ik weet niet hoe dat zit. Want kijk, de vraag is: in welke mate is er geluisterd naar Melany zelf? Ja, dat zou ik van haar zelf willen weten zonder dat anderen erbij zijn. En dat laat ik dan liever aan de POH-GGZ over om gewoon te kijken van: waar hebben we het nu over? Zit er nog ergens een greintje motivatie in en waardoor wordt het geblokkeerd? Wat zou ze eigenlijk zelf willen horen van haar ouders?
***Dus u zou een aparte afspraak inplannen voor Melany?***
Ja.
***U schetst een duidelijk beeld van uw gedachtengang en wat u zelf zou willen doen, niet alleen bij deze casus maar ook bij alle andere casus. Eigenlijk dezelfde vraag als die ik bij casus 2 ook al stelde: wat is nou het kernwoord of de kernwoorden waarin deze casus nou verschilt wat betreft uw gedachtegang, maar ook uw beleid, ten opzichte van de andere casus?***Ja, de leeftijd springt er uit. Ze is alweer een stuk verder, daarnaast komt eruit dat dit gescheiden ouders zijn. Die anderen hebben ouders die nog op hun kind letten, zorgen hebben. Dit zijn ook twee ouders die ruzie hebben, verschil van inzicht - behoorlijk - en hier heb je ook een kind die dingen doet die bedreigend zijn: zoals dronken zijn. Dus het is een soort destructief kind aan het worden. Ja dronkenschap, voor hetzelfde geld zijn er andere destructieve dingen. Dat zie je bij die anderen nog niet, die zijn nog constructief: die willen nog wel wat doen.
***Dus ook meer de verantwoordelijkheid van Melany in de problematiek?***Ja, Melany is echt destructief bezig.

***Ik ben in ieder geval in dit half uur een hoop wijzer geworden. Wat ik me verder nog afvroeg: we richten ons in de onderzoeksvraag ook op de samenwerking van huisartsen met JGT's danwel met specialistische zorg. We hebben heel kort net <naam specialistische GGZ-instelling> (<naam plaats>) genoemd. Hoe ervaart u die samenwerking als u daar nou mee in contact staat?***Dat is eigenlijk papieren samenwerking. Je stuurt iemand in en na een half jaar worden ze opgeroepen, of na 3 maanden. Dus die tussentijd moet je zien te overbruggen en dan krijg je een verslag terug. Het is eigenlijk net als een specialist in het ziekenhuis: je stuurt iemand in voor - laten we zeggen - darmkanker en na een half jaar krijg je bericht wat er allemaal gebeurd is. En ondertussen hoop je nog met een patiënt te bellen van hoe het gaat, is de vraag. Laten we zeggen: is die casus net zo erg als darmkanker? Maar soms zijn de gevolgen wel levenslang, dus dan denk je bij jezelf: heb je tijd als huisarts om tussentijds door feeling te houden met het gezin? Dat delegeer je dan aan de POH-GGZ.

***Oké, zodoende. En waarin vindt u die samenwerking nou verschillen met specialistische GGZ t.o.v. de JGT (daar heeft u natuurlijk niet heel veel contact mee, maar als die er is)?***
Nou, je hoopt eigenlijk dat JGT het snel oppakt; maar dat is niet altijd zo. Je hebt ook wachtlijsten, weliswaar korter en bij JGT weet ik ook niet hoe het met de privacy gaat - maar dit misschien... tegenwoordig heb je ook het Sociaal Wijkteam en daarvan is ook de vraag van hoe gaat het met privacy wat betreft medische gegevens? Als ik iemand doorstuur naar het sociaal wijkteam krijg ik ook nooit een berichtje terug, bijna niet. Dus ja, daar zijn nog wel vragen over van: hoe professioneel gaat dat nou? Is dat gewoon een actie van de regering om iets er in te stampen? Hoe zit dat?
***Allerlaatste vraag en dan laat ik u weer over aan uw drukke spreekuur, misschien (haha): ik wil toewerken naar een wetenschappelijk artikel. Dat hopen we ook wellicht internationaal te publiceren en we willen daarin ook toewerken naar aanbevelingspunten wat betreft samenwerking met JGT's maar ook met specialistische zorg; aanbevelingspunten voor in de huisartsenpraktijk, zeg maar. Wat zijn dingen waarvan u zegt: ''die moeten er echt in?'' of zijn er nog bepaalde aandachtspunten waarvan u zegt: ''dat moeten jullie meenemen.''?***Nou, zoals je al gemerkt hebt: als je mensen kent, zijn de lijntjes korter. En ik denk dat de korte lijntjes uitblinken in menselijkheid en wat je ziet in deze tijd dat het allemaal digitaal loopt. Versleuteld digitaal versturen, noem maar op. En ik denk dat de winst erin zou zitten doordat mensen elkaar meer laten zien. Dat kan zijn: JGT heeft natuurlijk ook te maken met scholen, met consultatiebureau's; dus als huisarts vraag ik me af: in welke mate zijn wij toeleverancier (?) van hun of een *core business* van hun? Kijk, ziekenhuizen zijn afhankelijk van huisartsen en weten dat ze zich moeten profileren. In welke maten kunnen ze profileren: hebben ze daar geld voor, budget voor, tijd voor? In welke mate is het uitgekleed? Als je kijkt, ik heb nog niet geGoogled hoor, maar als je Googled op Jeugd- en Gezinsteam; dan zie je een gezicht bij een persoon. Dan zie je wat hij doet, wat z'n specialiteiten zijn. Zijn de JGT's dat ze zich professionaliseren dat ze nieuwsbrieven naar huisartsen toesturen, ik noem maar iets. Vroeger, toen ik net begon, ging ik kennis maken met het *home team*. Dan wist je: dat was in een bepaalde wijk. Alleen, daar kwam beklad (?) in door bezuinigingen, noem maar op. Nou, nu komt het weer terug met <naam initiatief voor ouderenzorg> dat je weer in de wijk probeert bij elkaar te komen rond ouderen. Nou goed, dat zijn van die golfbewegingen in de historie die je ziet en gaan (?). Ja, wat een aanbeveling zou kunnen zijn zou zijn: meer profilering van de JGT en meer de boer op om het te melden. Op een gegeven moment, als huisarts houd je het - denk ik - de lijntjes waar je continuïteit hebt het langste vol. Dus daar gaat tenminste tijd in zitten. Als je als huisarts steeds moet inspelen op veranderingen, dat is natuurlijk heel leuk en soms moet je wel - dat kan niet anders - maar het blijkt dat dingen die continuïteit hebben, blijven langer bestaan; hebben meer waarde in zich omdat ze lang lopen. En dat is het voordeel van die kinderpsychologen: die zitten - net als huisartsen - heel lang in de wijk, voordeel van wijkverpleegkundigen die bijvoorbeeld ook heel lang in de wijk zitten die ken je dan en dat werkt. Maar als je elk jaar andere personeelsleden hebt, andere mensen, met andere insteek. Ja, als huisarts ben je wel flexibel, je kan heel snel ingrijpen, je weet: digitaal weet je al die adressen wel te vinden, maar nog is de vraag: werkt dat nou? ***Ja. Dus als ik het mag samenvatten: persoonlijk contact (het gezicht bij de naam en welke diensten ze kunnen leveren) en de continuïteit (de tijd die er overheen moet gaan)***.
Ja, kijk als je bijvoorbeeld naar <naam specialistische GGZ-instelling> stuurt - GGZ-instellingen -, <naam specialistische GGZ-instelling>, <naam specialistische GGZ-instelling>; dat kun je digitaal melden maar dat heb ik al die jaren heb ik daar nooit persoonlijk contact mee gehad. Als het dus echt professioneel is, dan lukt dat gewoon niet. Kijk, specialisten die heb ik allemaal wel een hand gegeven en ik weet hoe ze eruit zien en wat ze zijn. Maar, die zitten er ook al jaren, dus dat is een beetje het verschillende met groepen als <naam specialistische GGZ-instelling>, <naam specialistische GGZ-instelling> en andere psycholooggroepen: die verschuiven.
***Waardevolle punten, in ieder geval. Dan zal ik nu de opname stoppen.***

EINDE INTERVIEW
