# Supplementary material for: General practitioners’ everyday clinical decision-making on psychosocial problems of children and youth in the Netherlands
Source: PLoS One. 2022 Dec 28;17(12):e0278314. doi: 10.1371/journal.pone.0278314 (PMC9797081; doi:10.1371/journal.pone.0278314)

**Code Families**

______________________________________________________________________

HU: Analyse interviews

File: [C:\Users\Lennard\Documents\Onderzoek HAJGZJGT\Fase 1 interviews\Analyse interviews.hpr7]

Edited by: Lennard van Venrooij

Date/Time:2022-10-10 20:10:10

______________________________________________________________________

**Code Family: Beleidsplan**

Created: 2017-03-07 23:11:14 (Super)

Codes (12): [Communicatie andere hulpverlener(s)/derde(n) over te volgen beleid] [Doorverwijzen (basis-GGZ)] [Doorverwijzen (Jeugd- & Gezinsteam)] [Doorverwijzen (JGZ)] [Doorverwijzen (orthopedagoog)] [Doorverwijzen (overig)] [Doorverwijzen (specialistische GGZ)] [Eigen verantwoordelijkheid patiënt of ouders/verzorgers] [Realiseren gemeenschappelijke hulpvraag] [Volgorde van handelen] [Zelf behandelen] [Zorg voor ouders/verzorgers/broers of zussen]

Quotation(s): 223

______________________________________________________________________

**Code Family: Kennis**

Created: 2017-03-07 23:12:15 (Super)

Codes (6): [(Wettelijke) regelingen] [Bestaande iniatieven] [Geldstromen] [Jeugd- & Gezinsteam] [JGZ] [Specialistische GGZ]

Quotation(s): 46

______________________________________________________________________

**Code Family: Overig**

Created: 2017-03-07 23:16:48 (Super)

Codes (2): [Demografische gegevens / algemene informatie (niet op casuïstiek gericht)] [Herhalen informatie vraag / casuïstiek]

Quotation(s): 101

______________________________________________________________________

**Code Family: Probleemvorming**

Created: 2017-03-07 23:15:59 (Super)

Codes (13): [(Mogelijke) diagnose / verklaring probleem] [Consulteren (POH-GGZ)] [Ernst probleem] [Evaluatie voorgaand beleid] [Feitelijkheden] [Hulpvraag] [Manier van informatie inwinnen] [Observatie] [Opinie ouders/verzorgers] [Opinie overige personen] [Opinie patiënt] [Opinie school] [Vragenlijsten]

Quotation(s): 432

______________________________________________________________________

**Code Family: Samenwerken met andere zorgverleners**

Created: 2017-03-07 23:16:30 (Super)

Codes (3): [Ervaringen] [Ideeën ter verbetering] [Voorkeuren]

Quotation(s): 173

 **Codes divided by family**


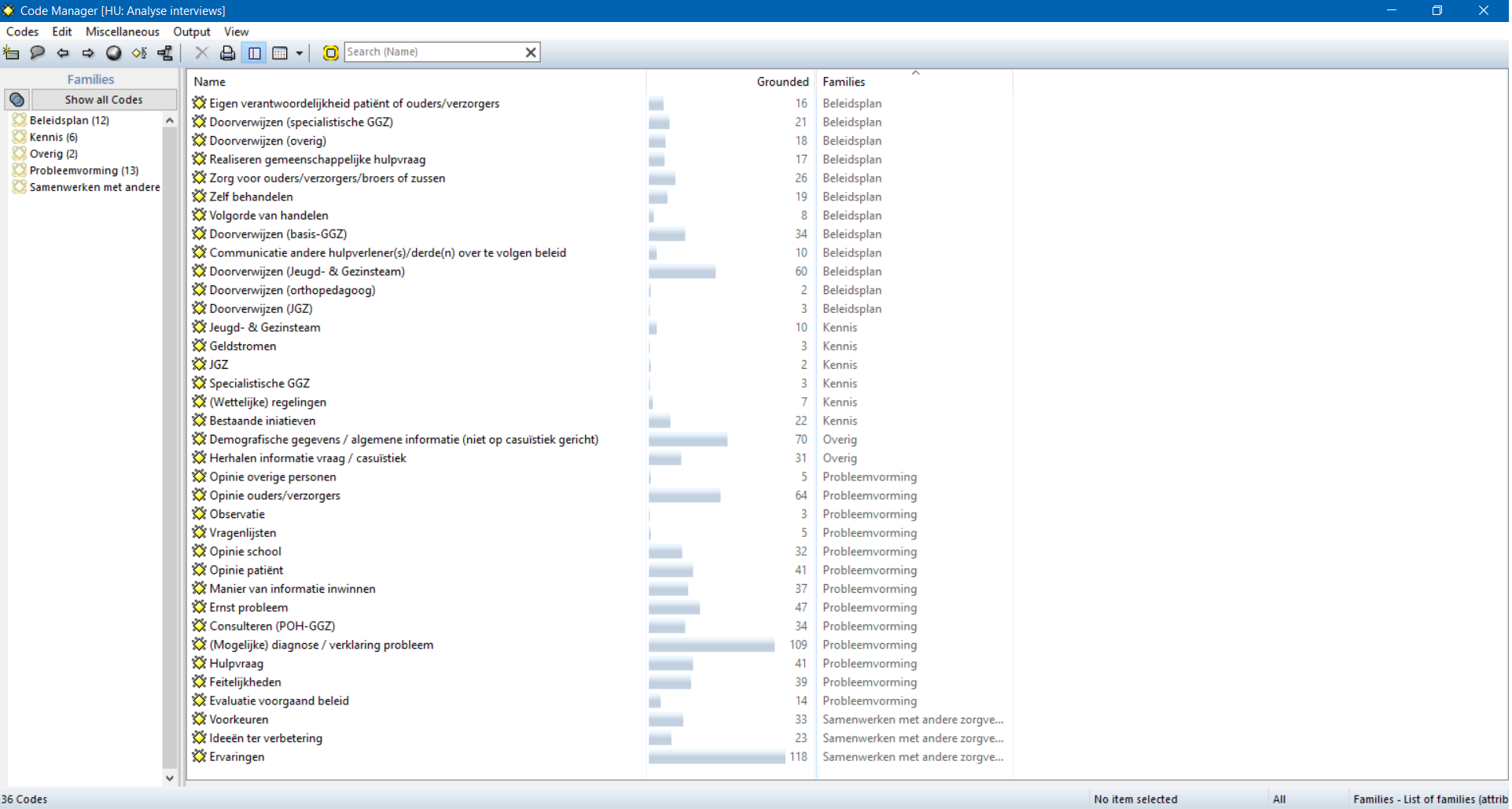

Supplement: S3 File — (DOCX) [file pone.0278314.s005.docx]
