# Supplementary figures and images for: General practitioners’ everyday clinical decision-making on psychosocial problems of children and youth in the Netherlands
Source: PLoS One. 2022 Dec 28;17(12):e0278314. doi: 10.1371/journal.pone.0278314 (PMC9797081; doi:10.1371/journal.pone.0278314)

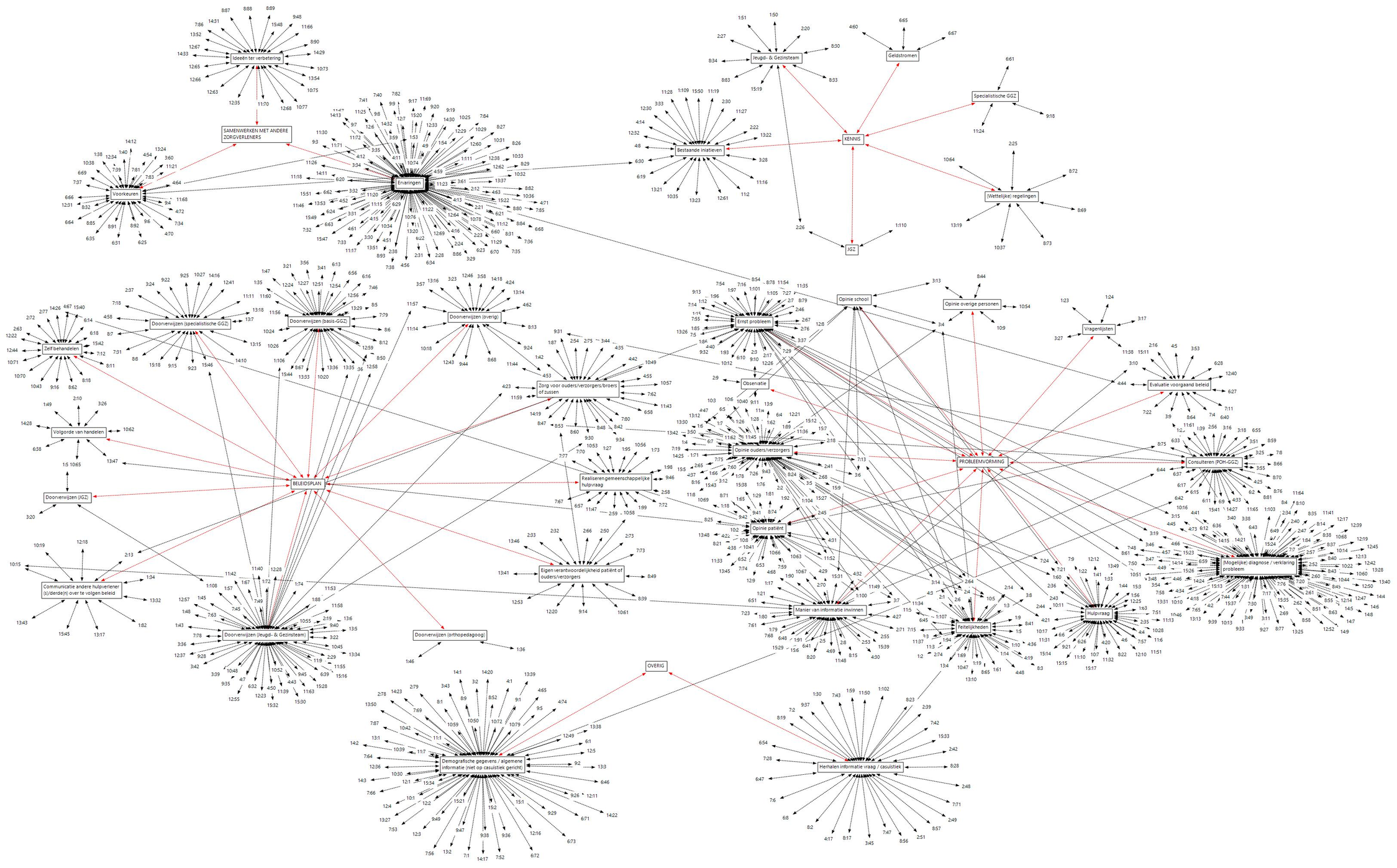

Supplement: S1 Fig — (TIF) [file pone.0278314.s006.tif]
